# A Genome-Wide RNAi Screen to Dissect Centriole Duplication and Centrosome Maturation in *Drosophila*

**DOI:** 10.1371/journal.pbio.0060224

**Published:** 2008-09-16

**Authors:** Jeroen Dobbelaere, Filipe Josué, Saskia Suijkerbuijk, Buzz Baum, Nicolas Tapon, Jordan Raff

**Affiliations:** 1 The Gurdon Institute, Cambridge, United Kingdom; 2 Apoptosis and Proliferation Control Laboratory, Cancer Research UK, London Research Institute, London, United Kingdom; 3 Research Institute Medical Research Council (MRC) Laboratory of Molecular Cell Biology, University College London (UCL), London, United Kingdom; Adolf-Butenandt-Institut, Germany

## Abstract

Centrosomes comprise a pair of centrioles surrounded by an amorphous pericentriolar material (PCM). Here, we have performed a microscopy-based genome-wide RNA interference (RNAi) screen in *Drosophila* cells to identify proteins required for centriole duplication and mitotic PCM recruitment. We analysed 92% of the *Drosophila* genome (13,059 genes) and identified 32 genes involved in centrosome function. An extensive series of secondary screens classified these genes into four categories: (1) nine are required for centriole duplication, (2) 11 are required for centrosome maturation, (3) nine are required for both functions, and (4) three genes regulate centrosome separation. These 32 hits include several new centrosomal components, some of which have human homologs. In addition, we find that the individual depletion of only two proteins, Polo and Centrosomin (Cnn) can completely block centrosome maturation. Cnn is phosphorylated during mitosis in a Polo-dependent manner, suggesting that the Polo-dependent phosphorylation of Cnn initiates centrosome maturation in flies.

## Author Summary

A major goal of the cell cycle is to accurately separate the duplicated chromosomes between two daughter cells. To achieve this, a pair of centrosomes organise a bipolar spindle made of microtubules; the chromosomes line up on the spindle and are then separated to the two spindle poles. Centrosomes are also required for the formation of cilia and flagella, which are present in many eukaryotic cells; centrosome dysfunction is a common feature of many human cancers and several neurological disorders, whereas mutations in genes that affect cilia function give rise to several human diseases. Here, we perform a genome-wide screen using RNA interference to try to identify all of the proteins required for centrosome function in the model organism Drosophila melanogaster (a fruitfly). We identified all 16 of the centrosomal proteins that were already known to be required for centrosome function in *Drosophila*, as well as 16 new centrosomal components or regulators. We confirmed the centrosomal location of several of the components and performed some analysis of their functions. We believe we are approaching a complete inventory of the proteins required for centrosome function in flies.

## Abstract 3

An RNAi screen identifies 16 new centrosomal components or regulators in*Drosophila*, and molecular dissection of their function addresses the role of Polo kinase in the maturation of pericentriolar material.

## Introduction

In most cells, the centrosome functions as the major microtubule (MT) organising centre (MTOC), and, as such, it has been implicated in organising many cellular processes, including vesicle transport, cell polarity, cell migration, and cell division [1,2]. There is also evidence that centrosomes have essential roles within the cell that are independent of their ability to organise MTs [3,4]. Indeed, many key regulators of cellular physiology, such as those required for cell cycle progression, cell signalling, and DNA damage response pathways, are concentrated at centrosomes, suggesting that the centrosome functions as a scaffold where many regulators meet and coordinate their response to various events in the life of the cell [5].

Centrosomes consist of a centriole pair surrounded by pericentriolar material (PCM). At the end of mitosis, the two centrioles disengage to allow duplication in the next cell cycle [6]. Subsequently, new centrioles are formed perpendicular to the mother centrioles in S-phase. As cells enter mitosis, the centrioles recruit PCM (a process termed centrosome maturation), and many MT nucleation and anchoring factors concentrate at the centrosomes as they form the poles of the mitotic spindle [5]. In addition to their function in organising the centrosome, centrioles also form the basal bodies present at the base of cilia and flagella, and cilia have been shown to have a variety of essential functions in development [7].

Centrosome amplification is a common feature of many cancers, and this has been linked to genetic instability, which is widely believed to be an important driver of tumourigenesis [8–12]. Furthermore, mutations in several human centrosomal proteins cause primary autosomal microcephaly, in which patients are born with small brains [13,14]. The reason for this phenotype is unclear, but it is postulated that centrosomes play a particularly important role during the asymmetric cell division of neural stem cells [15], and this is certainly the case in flies [16]. Finally, defects in cilia function have been identified as the cause of several human syndromes such as Bardet-Biedl syndrome (BBS) and Kartagener's syndrome, which lead to relatively pleiotropic defects during the development of affected individuals [17,18].

Although more than one hundred proteins are concentrated at centrosomes [5,19], it is unclear how these proteins are assembled into a functional unit, and how many of these proteins are actually required for centrosome function. Traditional genetic screens and genome-wide RNA interference (RNAi) screens in the early Caenorhabditis elegans embryo have identified just four proteins that are essential for centriole duplication (ZYG-1, SAS-4, SAS-5, and SAS-6), three that are essential for the recruitment of the PCM to the centrioles during mitosis (SPD-5, Protein Phosphatase-4 [PP-4], and the Aurora A kinase [AIR1]), and one that appears to have a role in both processes (SPD-2) [20–26]. Thus, a surprisingly small number of proteins appear to be essential for these “core” centrosomal functions in worms. Experiments in other systems, however, have identified many additional proteins that appear to have a role in centrosome maturation and/or centriole duplication ([5] and references therein; [27–35]). As the initial genome-wide screens in worms were not specifically designed to identify proteins required for centrosome function, it remains unclear how many proteins are required for the key functions of centriole duplication and centrosome maturation.

Here, we have performed a genome-wide RNAi screen in *Drosophila* tissue culture cells (S2R+) designed to identify proteins required for centriole duplication and centrosome maturation. After an extensive series of localisation studies and secondary screens, we have identified just 32 proteins that are required for these core centrosomal processes. Importantly, this screen successfully identified every *Drosophila* protein that had previously been implicated in centriole duplication and/or centrosome maturation, as well as several new factors, some of which have been implicated in centrosome function in other systems, and some of which are novel proteins that we confirm are components of the centrosome. Thus, we believe we are approaching a near-complete inventory of proteins required for these processes in flies. Finally, we noticed that only the depletion of either Polo kinase or Centrosomin (Cnn) could completely suppress centrosome maturation, indicating that they are major players in this process. We show that Cnn is phosphorylated exclusively during mitosis in a manner that is dependent on Polo kinase, and that these two proteins are codependent for their localisation at centrosomes. This suggests that the Polo-dependent phosphorylation of Cnn plays a crucial part in initiating centrosome maturation in flies.

## Results

### A Genome-Wide RNAi Screen for Proteins Required to Recruit Cnn to Mitotic Centrosomes

**Figure 1:**
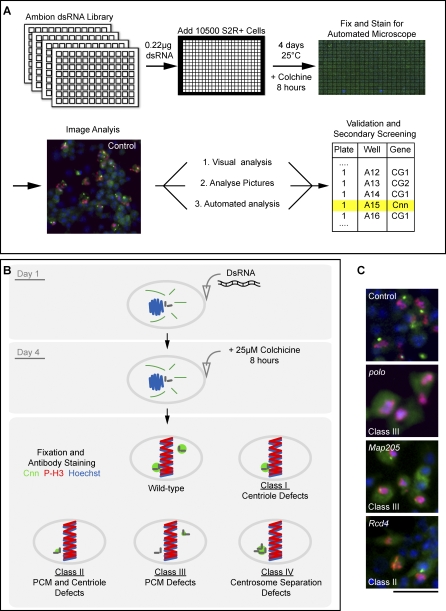
A Genome-Wide RNAi Screen for Centrosome Defects (A) A summary of the genome-wide RNAi screen. A dsRNA library was dispensed in 384-well plates suitable for high-throughput microscopy to a final concentration of 0.22 μg of dsRNA per well. Approximately 10.5 × 10^3^ S2R+ cells were aliquoted to each well and incubated for 4 d. Eight hours prior to fixation and cell staining, 25 μM colchicine was added to arrest cells in mitosis. Plates were manually analysed on a microscope, and pictures were then automatically acquired from four fields per well. Pictures were analysed manually and automatically using CellProfiler. Genes were selected for secondary screening if scored as “hits” with two of the three screening methods. (B) A schematic overview of the screening setup and expected phenotypes. The PCM is depicted in green, mitotic DNA in red, and DNA in blue. Centrioles, in grey, were not stained in the primary screen, but since the PCM is only nucleated around the centrioles [16], centriole duplication defects would be detected in this screen. (C) Examples of automated pictures from control-, polo-, Map205-, and Rcd4 (CG17295)-depleted wells. Scale bar represents 15 μm.

We devised a microscopy-based screen to search for proteins required for centriole duplication and centrosome maturation (Figure 1A and 1B). We used a library of double-stranded RNAs (dsRNAs) targeted against 13,059 individual genes (approximately 92% of all predicted protein-coding genes in Drosophila melanogaster) to deplete individual proteins in S2R+ cells. Treated cells were grown for 4 d in 384-well plates, then incubated with colchicine to depolymerise the MTs and arrest cells in mitosis for 8 h prior to fixation. The colchicine treatment increased the number of mitotic cells to facilitate the analysis, but did not interfere with centrosome maturation, which occurs robustly even in the absence of centrosomal MTs (Figure 1C). Cells were then fixed and processed for immunofluorescence microscopy with antibodies raised against phospho-histone H3 (p-H3) to identify mitotic cells and Cnn to label the PCM. The colchicine arrest often prevented proper centrosome separation and resulted in a mix of mitotic cells with one or two centrosomes (1.2–1.5 centrosomes per mitotic cell on average—see Materials and Methods).

We used anti-Cnn antibodies in our screen because Cnn is a very robust PCM marker, but also because Cnn appears to be a very general centrosome maturation factor: in its absence, the centrosomal recruitment of every other PCM component that has been tested is severely compromised during mitosis [36–38]. Thus, proteins that cause defects in the mitotic recruitment of Cnn to centrosomes are also likely to be general recruitment factors that are required for the proper recruitment of many other PCM components. Moreover, we reasoned that this screen would also identify proteins that are required for centriole duplication, as the PCM only assembles on the centriole scaffold in flies (Figure 1C) [16]. Thus, a reduction in centriole numbers would lead to fewer Cnn dots being observed and would therefore be detected in our screen.

In S2R+ cells, anti-Cnn antibodies only label centrosomes during mitosis (Figure 1C), as is true in many *Drosophila* cells in vivo [39,40]. The number of Cnn dots per mitotic cell was used as our readout in the primary screen. We quantified the number of centrosomes per mitotic cell after the depletion of individual proteins in three different ways (Figure 1A). First, each well of RNAi-treated cells was examined manually on a fluorescence microscope. Second, digital images of four fields of cells (typically containing more than 50 mitotic cells/field) from each well were acquired automatically and analysed manually. Third, these digital images were used to automatically count the number of centrosomes in each mitotic cell using CellProfiler [41] (see Materials and Methods). All of these analyses were performed “blind.” In this way, we identified 119 genes whose depletion significantly decreased or increased the average number of centrosomes per mitotic cell (Figure 1C and Table S1).

### Validation and Functional Screening to Differentiate between Proteins Required for Centriole Duplication and Centrosome Maturation

We performed an extensive series of secondary screens with 79 of these initial 119 hits. We used several criteria to exclude 40 genes that we thought less likely to be of interest for further analysis (see Table S1 and Materials and Methods), although we cannot exclude the possibility that some of these genes play a role in centriole duplication and/or centrosome maturation. We synthesised new, nonoverlapping, dsRNAs against these 79 genes (Table S3), and repeated the screen in both 384-well and 96-well formats with a 20× objective, but this time we examined the centrosomal localisation of Cnn, γ-tubulin, and DSpd-2 in both colchicine-, and noncolchicine-treated cells. All experiments were performed in triplicate to ensure the robustness of our screening procedures. Only 39 of the 79 genes tested were confirmed as positive hits after this analysis (Table S1). These 39 genes were then further tested in a set of functional assays that were specifically designed to distinguish whether individual proteins were required for centriole duplication, centrosome maturation, or both. We analysed the depletion of these 39 proteins in 24-well plates with a 63× objective using markers to detect centrioles (DSas-4), PCM (Cnn, DSpd-2, and γ-tubulin), and mitotic spindles (α-tubulin).

**Figure 2:**
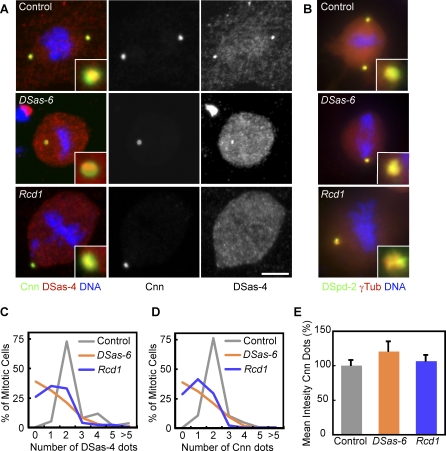
Genes Involved in Centriole Duplication (Class I) (A) S2R+ cells treated with dsRNA against GFP (control), DSas-6, and Rcd1 (CG8233) were stained with Hoechst (DNA, blue), DSas-4 (a centriole marker, red), and Cnn (a PCM marker, green). Inset shows a 4× magnified view. (B) Recruitment of DSpd-2 (green) and γ-tubulin (red) after dsRNA treatment for Control, DSas-6, and Rcd1. DNA is shown in blue, and inset shows a 4× magnified view. (C and D) Analysis of centriole (C) and centrosome (D) numbers in mitotic cells after RNAi treatment. More than 30 mitotic cells were counted in two independent experiments. (E) Analysis of PCM size in mitotic cells after RNAi treatment. The graph represents the mean intensity of PCM staining (Cnn) from three independent experiments, each analysing more than 20 centrosomes. Error bars represent the SE. Note how the number of centrioles and centrosomes per cell is reduced (C and D), whereas the amount of PCM recruited to the remaining centrioles is not affected (E) after DSas-6 and Rcd1 depletion. Scale bar in (A and B) represents 5 μm.

**Figure 3:**
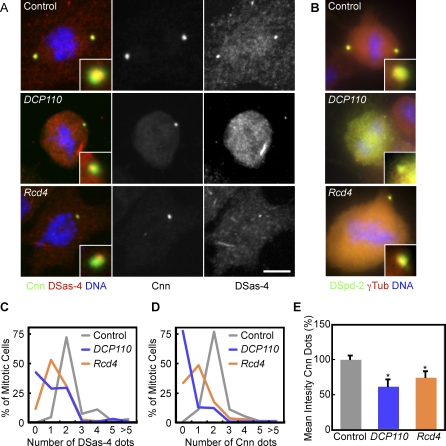
Genes Involved in Both Centriole Duplication and PCM Recruitment (Class II) (A) S2R+ cells treated with dsRNA against GFP (control), DCP110, and Rcd4 (CG17295) were stained with Hoechst (DNA, blue), DSas-4 (a centriole marker, red), and Cnn (a PCM marker, green). Inset shows a 4× magnified view. (B) Recruitment of DSpd-2 (green) and γ-tubulin (red) after dsRNA treatment for control, DCP110, and Rcd4. DNA is shown in blue, and inset shows a 4× magnified view. (C and D) Analysis of centriole (C) and centrosome (D) numbers in mitotic cells after RNAi treatment. More than 30 mitotic cells were counted in three independent experiments. (E) Analysis of PCM size in mitotic cells after RNAi treatment. The graph represents the mean intensity of PCM staining (Cnn) from three independent experiments, each analysing more than 20 centrosomes. Error bars represent the SE; an asterisk (*) indicates *p* ≤ 0.05 compared to control. Note how the number of centrioles and centrosomes per cell is reduced (C and D), and the amount of PCM recruited to the remaining centrioles is also reduced (E) after DCP110 and Rcd4 depletion. Scale bar in (A and B) represents 5 μm.

**Figure 4:**
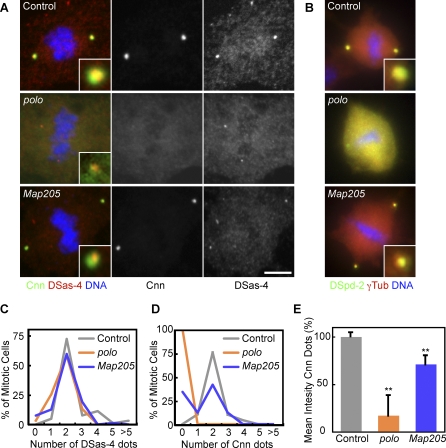
Genes Involved in PCM Recruitment (Class III) (A) S2R+ cells treated with dsRNA against GFP (control), polo, and Map205 were stained with Hoechst (DNA, blue), DSas-4 (a centriole marker, red), and Cnn (a PCM marker, green). Inset shows a 4× magnified view. (B) Recruitment of DSpd-2 (green) and γ-tubulin (red) after dsRNA treatment for control, polo, and Map205. DNA is shown in blue, and inset shows a 4× magnified view. (C and D) Analysis of centriole (C) and centrosome (D) numbers in mitotic cells after RNAi treatment. More than 30 mitotic cells were counted in two independent experiments. (E) Analysis of PCM size in mitotic cells after RNAi treatment. The graph represents the mean intensity of PCM staining (Cnn) from three independent experiments, each analysing more than 20 centrosomes. Error bars represent the SE; a single asterisk (*) or double asterisks (**) indicate *p* ≤ 0.05 or *p* ≤ 0.01 compared to control, respectively. Note how the number of centrioles per cell is not dramatically perturbed (C), but the number centrosomes (D) and the amount of PCM recruited to the centrioles (E) is reduced after polo and Map205 depletion. Scale bar (A and B) represents 5 μm.

**Figure 5:**
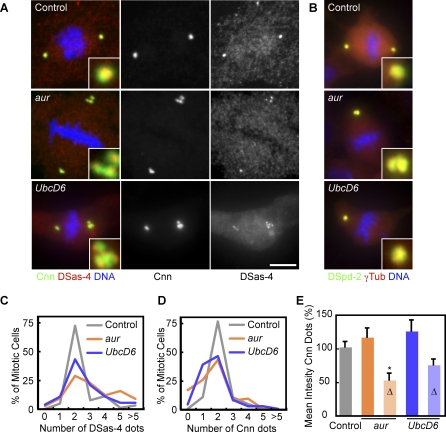
Genes Involved in Centrosome Separation (Class IV) (A) S2R+ cells treated with dsRNA against GFP (control), Aurora A (aur), and UbcD6 were stained with Hoechst (DNA, blue), DSas-4 (a centriole marker, red), and Cnn (a PCM marker, green). Inset shows a 4× magnified view. (B) Recruitment of DSpd-2 (green) and γ-tubulin (red) after dsRNA treatment for control, Aurora A, and UbcD6. DNA is shown in blue, and inset shows a 4× magnified view. (C and D) Analysis of centriole (C) and centrosome (D) numbers in mitotic cells after RNAi treatment. More than 30 mitotic cells were counted in two independent experiments. (E) Analysis of PCM size in mitotic cells after RNAi treatment. The graph represents the mean intensity of PCM staining (Cnn) from three independent experiments, each analysing more than 20 centrosomes. The lighter bars labelled with a Δ represent the PCM recruitment to the subset of the centrosomes that contained only one centriole dot. Error bars represent the SE. An asterisk (*) marks *p* ≤ 0.05 compared to control. This analysis suggests that PCM recruitment is impaired in cells depleted of Aurora A and UbcD6, but this effect is masked by the clustering of multiple centrosomes together. Scale bar in (A and B) represents 5 μm.

This analysis gave a final list of 32 genes whose depletion gave highly reproducible centrosome defects (Tables 1–4). For simplicity, we named any of these genes that had not previously been named, or that did not have homologs in other systems that had been assigned a function, Rcd proteins for “Reduction in Cnn Dots.” The 32 proteins were classified into four groups (Figure 1B; Tables 1–4). Nine proteins appeared to be required primarily for efficient centriole duplication (Class I, Figure 1B). The depletion of these proteins led to a reduction in the number of centrioles and centrosomes per cell, but in those cells that retained centrioles, the recruitment of the PCM was largely unperturbed (Figures 2 and S1). Nine proteins appeared to be required for both efficient centriole duplication and efficient PCM recruitment (Class II, Figure 1B). The depletion of these proteins led to a reduction in the average number of centrioles per cell, and in those cells that retained centrioles, the recruitment of the PCM to the centrioles was also reduced (Figures 3 and S1). Eleven proteins appeared to be required primarily for the efficient recruitment of the PCM to the centrioles (Class III, Figure 1B). The depletion of these proteins had only a minor effect on the average number of centrioles per cell, but significantly reduced the amount of PCM that was recruited to the centrioles (Figures 4 and S1). Finally, three proteins appeared to be required for centrosome separation (Class IV, Figure 1B). The depletion of these proteins led to an apparent reduction in the average number of centrosomes per cell, but staining with the centriole marker revealed that this was due to the clustering of several centrioles (Figure 5).

**Table 1:**
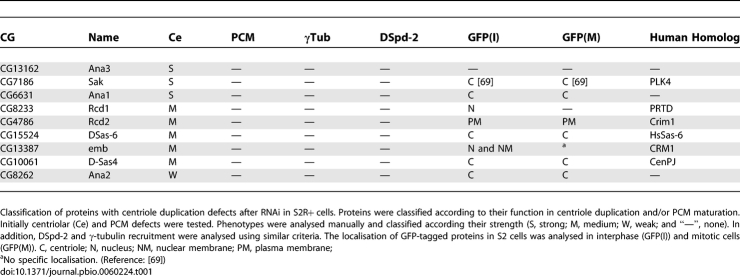
List of Proteins Involved in Centriole Duplication

To quantitate the defect in Cnn recruitment in cells depleted of each of these 32 proteins, we took optical sections through the entire cell volume and measured total centrosomal Cnn intensity. The average centrosomal intensity was measured in three independent depletion experiments (Figure S1—note that we typically analysed a total of ∼100 centrosomes in total, but in cases where centriole numbers were dramatically reduced, we could analyse only 20–40 centrosomes in total). Virtually all of the proteins classified as being required exclusively for PCM recruitment (Class III) showed a statistically significant decrease in the recruitment of Cnn to centrioles, but this was not true for any of the proteins classified as having a defect in only centriole duplication (Class I), strongly supporting the robustness of our scoring procedures. The proteins classified as being required for both PCM recruitment and centriole duplication (Class II), however, showed an intermediate phenotype: in eight of nine cases, the recruitment of Cnn was less than that seen in controls, but in only three cases was this difference statistically significant (Figure S1). As we consistently scored these proteins as having a defect in PCM recruitment in multiple experiments with multiple PCM markers, we suspect that this reflects the fact that the defect in PCM recruitment is more subtle in this class, and we would need to assay larger numbers of centrosomes to show statistical significance (see Discussion).

### Proteins Required for Centriole Duplication

The nine proteins we identified as being required for centriole duplication included the three proteins already known to be essential for this process in flies (DSas-4, DSas-6, and Sak/Plk4) as well as three proteins implicated in centriole duplication on the basis of their anastral spindle phenotype when depleted from S2 cells (Ana1–3) [42]. We created stable S2 cell lines expressing green fluorescent protein (GFP) fusions to Ana1 and Ana2 (we had difficulty in cloning full-length Ana3) under the control of either the metallothionein or ubiquitin promoter and found that they both localised to centrioles when expressed at low levels, as described previously [42] (Protocol S1, pages 5 and 11; and Table 1). When expressed at higher levels, Ana1 and 2 formed extra dots (usually 5–10) in the cytoplasm, a feature shared with the overexpression of GFP fusions to DSas-4, DSas-6, and Sak [43,44] (Figure S4). This suggests that, like these core centriole duplication proteins, Ana1 and Ana2 are structural components of the centriole required for efficient centriole duplication.

The three remaining proteins in this class have not previously been implicated in centriole duplication. Rcd1 (CG8233), Rcd2 (CG4786), and emb (CG13387) all have human homologs that have been implicated in various processes (Table S5), but none of these proteins were detectable at centrioles in stable cell lines expressing GFP fusions to any one of these proteins; instead these fusions localised to the nucleus, the plasma membrane, and nuclear membrane, respectively (Protocol S1, pages 6, 7, and 9; and Table 1). Thus, although it is possible that GFP-tagging disrupts the centriolar localisation of one or more of these proteins, it seems likely that they influence centriole duplication indirectly.

### Proteins Required for Centriole Duplication and Centrosome Maturation

The nine proteins identified as being required for both centriole duplication and PCM recruitment include four that have previously been implicated in centriole/centrosome function either in flies or in other systems (Table 2). Asterless (Asl; CG2919) is a centrosomal protein previously shown to be required for efficient PCM recruitment in flies, and it is related to the human centrosomal protein Cep152 [45] (Table S5). Asl-GFP localised to both centrioles and the PCM, as shown previously [45]; as with the overexpression of DSas-4, DSas-6, Sak, Ana1, and Ana2, its overexpression led to the formation of extra dots in cells (Figure S4A and S4B). Thus, we conclude that Asl is required for both centrosome maturation and centriole duplication in flies.

**Table 2:**
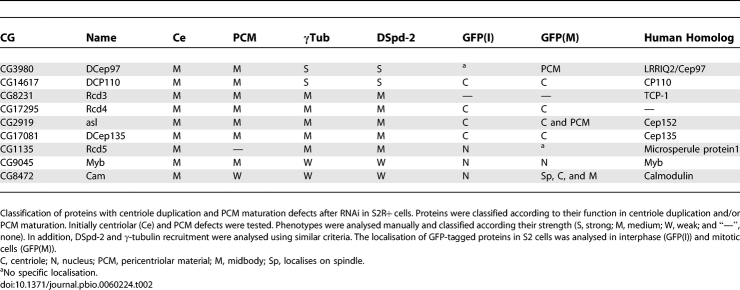
List of Proteins Involved in Centriole Duplication and PCM Maturation

**Table 3:**
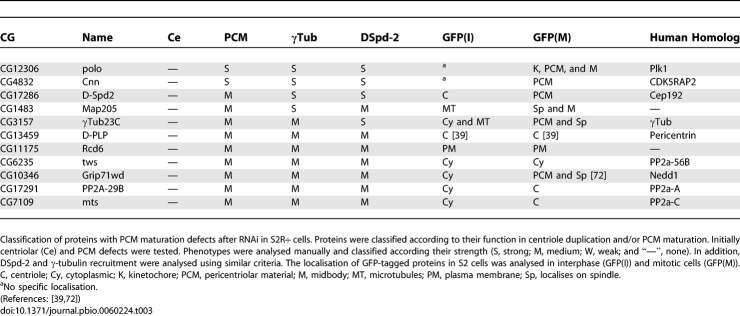
List of Proteins Involved in PCM Maturation

**Table 4:**
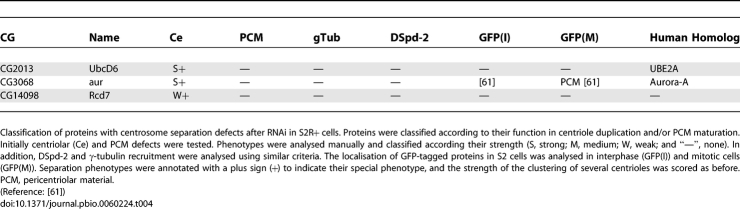
List of Proteins Involved in Centrosome Separation

**Figure 6:**
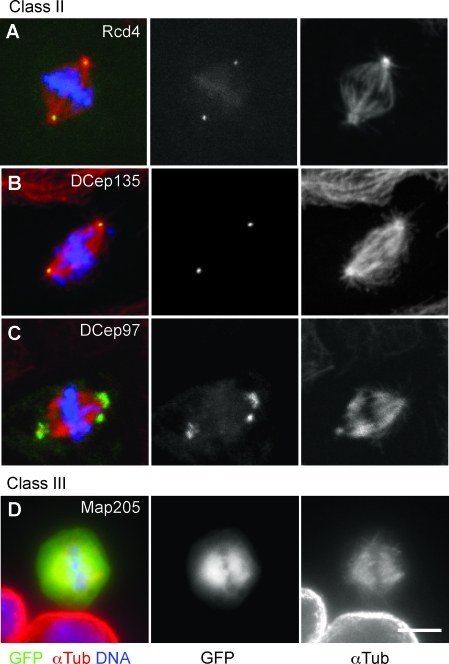
Localisation of Several Newly Identified Centrosome/Spindle Components by GFP-Tagging S2 cells stably transfected with GFP-constructs expressing Rcd4 (CG17295) (A), DCep135 (B), DCep97 (C), or Map205 (D) under the control of the metallothionein promotor (pMT) were induced for 24 h, fixed with paraformaldehyde, and costained with Hoechst (DNA) and anti–α-tubulin antibodies. The merged picture shows the GFP-fusion protein in green, α-tubulin in red, and DNA in blue. Scale bar represents 5 μm.

CG17081 is the fly homolog of human Cep135, CG14617 is the fly homolog of human CP110, and CG3980 is the fly homolog of Cep97; all of these proteins have been implicated in centriole duplication and PCM recruitment in humans [19,27,29,46]. We found that when expressed at low levels, GFP fusions to *Drosophila* Cep135 (DCep135) and *Drosophila* CP110 (DCP110) were concentrated at centrioles; interestingly, however, high-level overexpression of either protein led to the formation of fibre-like structures in the cytoplasm, most prominently in the case of DCep135 (Figures S4A; Protocol S1, pages 14 and 18). In contrast, a GFP fusion to *Drosophila* Cep97 (DCep97) localised to centrosomes specifically during mitosis (Figures 6D; Protocol S1, page 13; and Table 2). Together, these findings indicate that these four proteins are very likely to play a direct role in centriole duplication and/or centrosome maturation (see Discussion).

Two of these nine hits, Myb and Rcd5 (CG1135), were recently found in a screen to identify proteins involved in mitotic spindle function, but their exact defects were not characterized [42]. Myb is a transcription factor that has a variety of cell cycle–related functions [47], but GFP-Myb fusions did not detectably localise to centrioles or centrosomes, suggesting Myb's role at centrosomes may be indirect (Protocol S1, page 20; and Table 2). Interestingly, it has recently been shown that perturbing Myb function leads to a reduced Polo levels, perhaps explaining its influence on PCM recruitment [48].

Interestingly, Rcd5 (CG1135) was unique amongst all of the proteins we analysed in that it had only a slight effect on the amount of Cnn recruited to centrioles during mitosis (and it was picked up in our original screen primarily because of the reduction in the number of centrioles in depleted cells), but the amount of γ-tubulin and DSpd-2 recruited to centrosomes was more dramatically decreased, hence the inclusion of Rcd5 in Class II (Protocol S1, page 19; and Table 2). Thus, Rcd5 may act downstream of Cnn in the pathway that leads to DSpd-2 and γ-tubulin recruitment. GFP fusions to this protein were not, however, detectably concentrated at centrosomes (Protocol S1, page 19; and Table 2).

None of the three remaining proteins in this class have previously been implicated in centriole duplication or centrosome maturation. Calmodulin, however, has been implicated in targeting several proteins to centrioles and centrosomes, including CP110 [28], and a GFP-calmodulin fusion protein localised to centrosomes and spindles specifically during mitosis (Protocol S1, page 21). Rcd4 (CG17295) is not obviously related to any protein outside of insects, but GFP fusions to Rcd4 strongly localised to centrioles (Figure 6; Protocol S1, page 16). Thus, these two proteins are likely to have direct roles in centriole function. Rcd3 (CG8231) is homologous to the human T-complex protein 1 subunit zeta, which is needed for proper tubulin folding [49], so the observed defects are probably indirect.

### Proteins Required for Centrosome Maturation

The 11 proteins required for centrosome maturation (Table 3) include five of the six proteins that have previously been implicated in this process in flies: Cnn [36–38], Polo [50], DSpd-2 [51,52], D-PLP [39], and γ-tubulin [53]. The only protein of this type that we did not identify in our screen was Aurora A [54], which we found to be required for centrosome separation, but which is probably also required for PCM recruitment (see below).

Of the six remaining proteins in this class, Grip71WD is a centrosomal protein that is homologous to GCP-WD/NEDD1 in humans. Although it was thought not to be required for PCM recruitment in flies [55], Grip71WD has been implicated in PCM recruitment and centriole duplication in humans [31,56]. Our data suggest that this protein has some function in centrosome maturation flies. The MT-binding protein Map205 is localised to centrosomes and MTs [57] (Figure 6; Protocol S1, page 26), but null mutants in this gene are viable and fertile [57], demonstrating that its function is not essential in flies. Rcd6 (CG11175) is predicted to encode a transmembrane protein, and GFP fusions were predominantly localised to the plasma membrane, suggesting that any role in centrosome maturation is indirect (Protocol S1, page 29).

Surprisingly, the three remaining proteins in this class encode the catalytic subunit (mts), a regulatory subunit (tws), and a structural subunit (PP2A-29B) of the protein phosphatase PP2A, thus providing compelling evidence that this enzyme is essential for efficient PCM recruitment in flies. Components of PP2A are associated with centrosomes in human cells [19], and with the centrosome equivalents in fission yeast and *Dictyostelium* [58,59], but GFP fusions to any of these fly proteins were not detectably concentrated at centrosomes in our hands (Protocol S1, page 30; and Table 3). Although PP2A activity is required for many cell processes, this form of PP2A (PP2A^tws^) seems to be the only one that is essential for centrosome maturation; we tested the effect of depleting the three other PP2A regulatory subunits either individually, or in all combinations, and found that none of these were required for efficient centrosome maturation (J. Dobbelaere, unpublished data).

### Proteins Required for Centrosome Separation

To our surprise, Aurora A, together with the ubiquitin E2 ligase UbcD6 and the protein of unknown function Rcd7 (CG14098), were recovered in our screen as being required for centrosome separation (Table 4). These proteins were picked up in our primary screen because they were originally scored as having too few centrosomes per cell (Table S1). Our secondary screening revealed, however, that cells depleted of these proteins appeared to have too few centrosomes because they had not separated properly (Figure 5). Although Aurora A has previously been implicated in PCM recruitment in worms and flies [54,60], a centrosome clustering phenotype has been described previously in flies [61], and we confirmed that this is the dominant phenotype we observed in *aurora A* mutant larval brain cells (Figure S2). It seems likely, however, that this centrosome separation defect masks a role for Aurora A and UbcD6 in PCM recruitment, as the single centrioles that we observed in these cells were found to recruit less PCM than normal (Figures 5 and S1).

### Polo and Cnn Appear to Be Key Initiators of Centrosome Maturation

**Figure 7:**
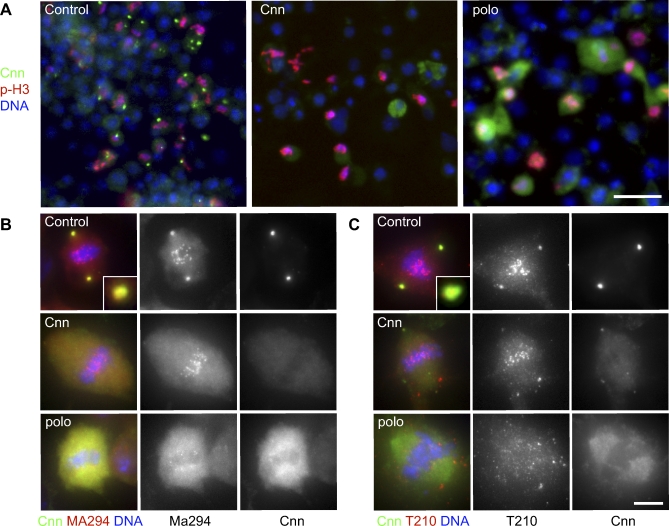
Polo and Cnn Are Interdependent for Their Localisation and Function at Centrosomes (A) Pictures from the primary screen of S2R+ cells treated with dsRNA against GFP (control), Cnn, and polo. The localisation of Cnn (green), Phospho-histone H3 (red), and DNA (blue) is shown. (B) S2R+ cells treated with RNAi against GFP, Cnn, and Polo were stained with antibodies against Cnn (green) and Polo (red), and counterstained with Hoechst (blue). (C) S2R+ cells treated with RNAi against GFP, Cnn, and polo were stained with antibodies against Cnn (green) and “active” Polo (red), and counterstained with Hoechst (blue). Note how the depletion of Cnn disrupts the centrosomal, but not the kinetochore, localisation of Polo, whereas the depletion of Polo disrupts the centrosomal localization of Cnn. Scale bar in (A) represents 15 μm, in (B and C), it represents 5 μm.

**Figure 8:**
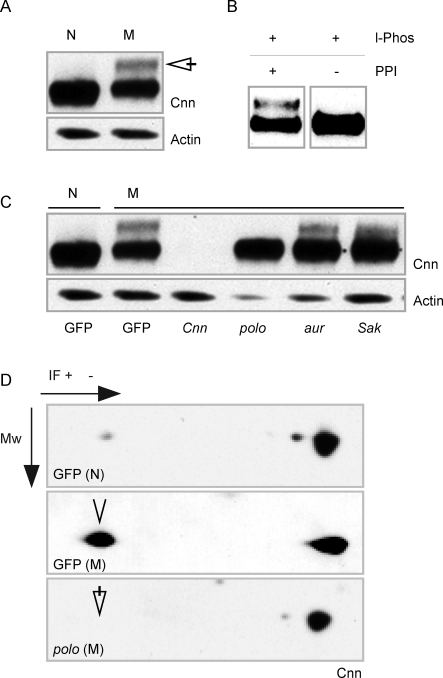
Cnn Is Phosphorylated in Mitosis in a Polo-Dependent Manner (A) Western blot showing the phosphorylation of Cnn (indicated by the band shift in the 1D gel—arrow) in extracts from nontreated cycling S2R+ cells (N) and extracts enriched for mitotic cells by colchicine treatment (M). Actin was used as a loading control. (B) Extracts enriched for mitotic cells were treated with λ-phosphatase in the presence or absence of phosphatase inhibitors. (C) Western blot showing the behaviour of Cnn in extracts from nontreated cycling cells (N) and extracts enriched for mitotic cells by colchicine treatment (M) after treatment with dsRNA against control (GFP), Cnn, polo, Aurora A (aur), and Sak/Plk4 for 4 d. The depletion of only Polo blocks the formation of the phosphorylated form of Cnn. Note that the total protein loaded in the Polo depletion shown here is slightly reduced compared to the other lanes. The upper (phosphorylated) form of Cnn, however, was undetectable on much longer exposures of this blot, and we consistently failed to detect this upper band in several independent Polo-depletion experiments (unpublished data). Thus, we are very confident that the absence of this band from the Polo-depleted cells is not simply due to the lower amount of protein loaded in this lane. (D) Western blot analysis of a 2-D gel of nontreated cycling S2R+ cell extracts (N) or extracts enriched for mitotic cells by colchicine treatment (M) treated with a control dsRNA (GFP) or a dsRNA against Polo. The phosphorylated form of Cnn is enriched in control (GFP) mitotic extracts (arrowhead), but is not present in mitotic extracts from Polo-depleted cells (polo—arrow). Note that the depletion of Polo does not dramatically alter the proportion of cells in mitosis in any of these depleted cells treated with colchicine (∼20%–35% in all cases—as judged by phospho-histone H3 staining).

From our analysis of all the proteins we identified as being required for efficient centrosome maturation, it was clear that the depletion of Cnn or Polo had a significantly stronger effect on this process than the depletion of any other protein (Figures 7A and S1, and Protocol S1, pages 23 and 24—note that for Cnn, this was judged by the strength of its effect on the centrosomal recruitment of γ-tubulin and DSpd-2). This suggests that these two proteins have a particularly important role in centrosome maturation in flies. Since Polo is known to localise to centrosomes in mitosis [50,62] (Figure 7), we tested whether Polo might initiate centrosome maturation by phosphorylating Cnn. Western blotting experiments revealed that Cnn was indeed phosphorylated specifically during mitosis, and that this phosphorylation was dependent on Polo, but not on the centrosomal kinases Aurora A or Sak/Plk4 (Figure 8). Moreover, Cnn and Polo exhibited a reciprocal dependency for their localisation at centrosomes: Cnn was essentially undetectable at centrosomes in cells depleted of Polo, whereas Polo and activated Polo—detected with antibodies raised against Polo phosphorylated on the activating T210/T182 (in humans and flies, respectively)—were undetectable at centrosomes in cells depleted of Cnn (Figure 7B and 7C). These observations raise the intriguing possibility that it is the Polo-dependent phosphorylation of Cnn that initiates centrosome maturation in flies.

## Discussion

In this study, we set out to identify proteins required for centriole duplication and centrosome maturation in *Drosophila* S2R+ cells. As well as recovering all known *Drosophila* proteins that had previously been implicated in these processes, we identified several fly homologs of centrosomal proteins previously identified in other systems, and several new proteins that had not previously been implicated in centrosome function, some of which have homologs in other systems. We show that several of these new proteins are centrosomal components, indicating that they probably have a direct role in centrosome function.

One surprising aspect of our results was the identification of a relatively large number of proteins (nine) that appear to be required for both centriole duplication and centrosome maturation (Table 2). It is unclear, however, whether these proteins have separate functions in these processes. Previous studies in worms and human cells have revealed that although centrosome maturation is not essential for centriole duplication, the recruitment of at least some PCM components to the centrioles is required for this process to occur efficiently [63–65]. Thus, although the proteins we identify in this class do not have a particularly strong defect in centrosome maturation (compared to Cnn and Polo, for example, which have stronger defects in centrosome maturation, but no defects in centriole duplication), it may be that these proteins play a particularly important part in recruiting a small amount of PCM to the centrioles during S-phase, and that this is required for efficient centriole duplication. Alternatively, some or all of these proteins may only be required for efficient centriole duplication, but their partial depletion may lead to the formation of defective centrioles that no longer efficiently recruit PCM. Further investigation will reveal how these proteins regulate these two processes, but it is clear that Asl/Cep152, DCep135 (CG17081), DCP110 (CG14617), DCep97 (CG3980), Rcd4 (CG17259), (which so far has no homolog outside of insects), and calmodulin are all centrosomal components that are required for efficient centriole duplication and/or efficient PCM recruitment in fly cells.

Studies in worm embryos have identified just five proteins that are required for centriole duplication, and these have been ordered into a functional pathway: SPD-2 recruits the kinase ZYG-1, which recruits SAS-5 and SAS-6, which in turn recruit SAS-4 [20–26]. Proteins related to ZYG-1, SAS-6, and SAS-4 are required for centriole duplication in several other systems, and it has been postulated that these five proteins constitute a conserved “core” centriole duplication machinery [66]. Previous studies in fly cells suggested that three additional proteins (Ana1–3) may also be required for centriole duplication (inferred from a lack of astral MTs in spindles and absence of γ-tubulin at the poles when these proteins were depleted), and Ana1 and Ana2 were shown to localise to centrioles [42]. We have confirmed these results and extended them by directly showing that centriole numbers decrease in cells depleted of Ana1–3. Further experiments will be required, however, to determine whether these proteins are part of the conserved “core” centriole duplication machinery.

It is worth noting that whereas SPD-2 is a key initiator of centriole duplication in worm embryos [25,26], DSpd-2 was only picked up in our screen as being required for PCM recruitment (see below), consistent with previous analyses of DSpd-2 mutant flies [51,52]. Whether human Spd-2/Cep192 has a role in centriole duplication that is independent of its role in PCM recruitment remains controversial [67,68]. Thus, the exact role of this family of proteins in centriole duplication and PCM maturation remains to be clarified.

We believe we have now identified most, if not all, of the major structural components required for general PCM assembly during mitosis (see below). Cnn, DSpd-2, D-PLP, γ-tubulin, and Grip71WD are all components of the PCM, whereas Map205 is a MT-associated protein that is present in the PCM. Polo and a specific form of PP2A appear likely to play regulatory roles in this process. Moreover, although the depletion of Aurora A and UbcD6 causes primarily a centriole-clustering phenotype, the recruitment of PCM to individual centrioles is reduced when either of these proteins is depleted, indicating that they too play a part in centrosome maturation. Although it remains unclear how these proteins work together to drive centrosome maturation, the individual depletion of two of these proteins, Cnn and Polo, consistently perturbed centrosome maturation to a greater extent than the depletion of any of the other proteins. This indicates that these two proteins may initiate the centrosome maturation pathway in flies. In support of this possibility, we found that Cnn is specifically phosphorylated during mitosis in a Polo-dependent manner. More experiments are required, however, to determine whether Polo phosphorylates Cnn directly, and whether this phosphorylation event really initiates centrosome maturation, or is simply correlated with it.

Interestingly, it has previously been postulated that Cnn functions primarily to “strengthen” the structure of the PCM, thus preventing the PCM from dissipating away from the centrosomes soon after it is recruited [38]. An attractive model is that the Polo-dependent phosphorylation of Cnn may initiate centrosome maturation by allowing Cnn to strengthen the PCM. In such a scenario, the centrioles would actively recruit PCM at all stages of the cell cycle, but in the absence of phosphorylated Cnn, the PCM is structurally weak, and it cannot accumulate to any extent around the centrioles. As cells enter mitosis, Polo phosphorylates Cnn (either directly or indirectly), thus allowing it to strengthen the PCM, which can then accumulate around the centrioles.

An important question is whether the proteins we identify here represent a complete list of those required for centriole duplication and centrosome maturation in flies. Clearly, we may have missed some proteins. Our screen probed only approximately 92% of protein-coding genes, and 108 proteins could not be tested because there were not enough mitotic cells to be scored after their depletion. In addition, some proteins may not have been detected because they are poorly depleted by RNAi, or because their depletion produced such pleiotropic defects that centrosome defects could not be scored properly. On the other hand, all 13 of the known fly proteins previously implicated in centrosome maturation (Polo, Aurora A, Cnn, DSpd-2, D-PLP, Asl, and γ-tubulin) or centriole duplication (DSas-4, DSas-6, Sak, Ana1, Ana2, and Ana3) were successfully identified in our screen. This is despite the fact that many centriolar proteins are known to be difficult to deplete by RNAi [42,69] (J. Dobbelaere, unpublished data). Moreover, the depletion of proteins such as Polo and Aurora A clearly produces pleiotropic mitotic defects, yet both proteins were successfully identified in our screen.

Taken together, these observations suggest that it is unlikely we are missing large numbers of proteins from this list, and that we are at least approaching a near-complete inventory of the proteins required for centriole duplication and centrosome maturation in flies. Although this list is significantly larger than the list that has emerged from studies in worm embryos, it is still surprisingly small, and we conclude that only a relatively small subset of the many proteins concentrated at centrosomes is actually essential for the key centrosomal functions of duplication and maturation. Clearly, this extensive dataset provides an important framework with which to delineate the events that drive the centrosome cycle.

## Materials and Methods

### Preparation of the *Drosophila* RNAi library.

An RNAi library covering nearly the whole *Drosophila* genome was purchased from Ambion (Silencer(R) *Drosophila* RNAi Library, AM85000). This library comprises dsRNAs designed against 13,059 *Drosphila* genes, or approximately 92% of all currently known protein-coding genes (Flybase). The original library, in 96-well plates, was replated onto clear bottom 384-well plates (Corning #3712) to a final concentration of 0.22 μg of dsRNA/well in 5 μl (1× PBS) using a Beckman Biomek FX. Controls were added in the upper left and lower right corner of each plate. dsRNA against DsRed was used as a negative control. dsRNA against Scar, String, and Thread were added as controls for cell morphology, division, and cell death. Finally, dsRNA against Polo and Cnn were added as positive controls to every 384-well plate for this specific screen.

### RNA interference, cell staining, and image acquisition.

For the primary screen, S2R+ cells were cultured in Shields and Sang medium (Sigma S3652) with 10% FBS (Sigma F9665) and 1% penicillin/streptomycin (Gibco 15070–063). After trypsinising the cells, they were diluted to 7 × 10^5^ cells/ml in serum-free Shields and Sang medium. A total of 15 μl of cells were added to the dsRNA-containing 384-well plates using a Thermo Wellmate (giving a final concentration of ∼10,500 cells per well). Plates were gently spun, and cells were incubated for 30–45 min, and 35 μl of serum-containing medium was added. Plates were sealed and incubated for 4 d at 25 °C. Eight hours prior to fixation, we exchanged the medium for medium containing 25 μM colchicine (Sigma #C3915), a microtubule depolymerising drug that arrests cells in mitosis (this typically resulted in 20%–35% of the cells in a well being in mitosis at the time of fixation). Cells were washed once with PBS, fixed with 4% formaldehyde (in PBS) (Sigma #F8775) for 12 min, and permeabilised with 0.5% SDS in PBS for 10 min. Cells were blocked with 5% goat serum (Sigma G9023) in PBS-T (0.1% Triton) for 20 min and stained overnight at 4 °C with anti-Cnn antibodies (1:1,000, rabbit) to stain centrosomes [38] and anti-pH3 Ser10 antibodies to label mitotic cells (1:2,000, mouse; Abcam 14955). Antibodies were diluted in PBS-T with 5% goat serum. The next day, cells were washed three times with PBS-T for 5 min. Secondary antibodies, anti-rabbit Alexa 488 (1:1,500; Molecular Probes A21206) and anti-mouse Alexa 567 (1:1,500; Molecular Probes A11004), in 5% goat serum in PBS-T were added for 2 h at room temperature. Cells were washed once with PBS-T, incubated with Hoechst 33258 (final concentration of 0.2 μg/ml; Sigma #861405) in PBS for 10 min, and then washed once more with PBS-T. Finally, 20 μl of PBS was added to each well, and plates were sealed with aluminium sealing tape (Corning #6569).

Specimens were imaged on a Nikon TE2000E microscope, with an automated Prior stage controlled with Metamorph software (Molecular Devices) using a 20×, 0.45NA, Plan Fluor air objective. After automated focusing, we took pictures of the three channels (Hoechst, Alexa 488, and Alexa 567) at four different sites per well (an average total of 500–2,000 cells, approximately 150–400 of which were usually in mitosis). All primary pictures and annotation are available on the Flight database (http://flight.licr.org/)

For the secondary screening assays, RNAi was performed as above (using 0.22 μg, 0.6 μg, 2 μg, or 10 μg of dsRNA per well for 384-well, 96-well, 24-well, or 6-well plates, respectively). Detailed immunofluorescence analysis of centrioles and PCM was performed by adding a glass cover slip before seeding the cells in 24-well plates and analysing the cells on a Perkin Elmer Ultraview ERS spinning disk system on a Zeiss Axioskop II microscope using a 63×, 1.4NA, Plan Apo oil objective. Antibodies used in the secondary screen were rabbit anti-DSpd2 (1:500; [51]), rabbit anti-DSas4 (1:500; [16]), mouse anti–γ-tubulin (1:500; GTU-88 Sigma), and mouse anti–α-tubulin (1:1;000; DM1α Sigma). Twenty images at 0.25-μm separation in the *Z*-axis were taken in each channel, and a maximum-intensity *Z*-projection was made using the Ultraview ERS software. Note that the anti–DSas-4 antibodies usually cannot distinguish between a single centriole and a centriole pair (as centriole pairs usually stain as a single dot in these cells with this antibody).

### Image analysis and statistical analysis.

To identify proteins that give centrosome defects after depletion with dsRNA, we scored each well by three different methods. First, each well was inspected manually on the widefield microscope system described above, and given a numerical score (from −3 to 3) for the severity of any defect in cell number, mitotic index, centrosome number, and centrosome size. Second, the pictures taken with the automated microscope were manually scored using the same criteria. All of these analyses were performed “blind,” so that we did not know which genes were being analysed. Finally, the pictures were analysed with CellProfiler (http://www.cellprofiler.org) [41] using a self-made pipeline (See Text S1). This resulted in a numerical value for the number of Cnn dots per mitotic cell. The inverse of this numerical dataset was normalised (plate average was set to zero) and corrected for plate-by-plate variations and possible edge effects using the CellHTS software ([70], using the *B*-score method) (See Figure S3). The *Z*′-score was calculated using Cnn and Polo as positive controls, and all empty and DsRed wells as negative controls. This analysis enabled us to give a statistical significance to each potential hit. A total of 108 genes were excluded from both the manual and the automated analysis because of the lack of cells or lack of mitotic cells in the well (Table S2); 119 genes were selected for secondary analysis as they were scored as hits with at least two of these three methods. From these 119 genes, only 79 were selected for a more detailed secondary analysis, as we eliminated genes that were commonly identified in previous screens (indicating they are likely false positives), were known components of the ribosome or transcription machinery, or were the result of clear off-target effects (Table S1).

For the secondary analysis, centriole (DSas-4) and centrosome (Cnn) number (shown in the graphs associated with each gene in Protocol S1) were quantified as follows. Maximum intensity *z*-projections from two independent experiments (at least 30 mitotic cells per experiment) were analysed, and the number of centrioles per mitotic cell were counted. The amount of PCM accumulated around each centriole was scored by eye as either normal or small/absent. For the quantification of PCM recruitment shown in Figures 2E, 3E, 4E, 5E, and S1, PCM size was quantified by measuring the background-corrected mean intensity of the Cnn dots in the *z*-projected image. Average intensities (normalised against control RNAi set to 100%) are represented from three independent experiments (typically 20–40 Cnn dots were counted per experiment, but occasionally only 10–15 Cnn dots were counted for proteins whose depletion meant there were very few centrosomes that could be counted). The statistical significance was measured using a dual-tailed *t*-test. *p* ≤ 0.05 are marked on the graphs by a single asterisk (*), and *p* ≤ 0.01 are marked with double asterisks (**).

### GFP-tagging of proteins identified in the genome-wide screen.

Vectors allowing the expression of GFP-tagged proteins were made using the Gateway system (Invitrogen). A list of the primers used is shown in Table S4. Constructs for all genes, unless otherwise stated (Table S4), were made for both N- and C-terminal (NT and CT, respectively) tagging. Forward primers for NT- and CT-tagging were the same (including ATG), but the NT reverse primer included the STOP codon, whereas the CT-primers lacked the STOP codon. All genes were cloned from cDNA unless stated otherwise (Table S4). Once cloned in the pZEO-Entry vector, inserts were checked by restriction digest and most of them also by sequencing (Table S4).

The genes were then recombined into the expression vectors pMT (Invitrogen) and pwUbq (gift from R. Basto), placing the genes under the control of the metallothionein and ubiquitin promoters, respectively. Transfection of the expression vectors in S2 cells was performed as described previously [71]. Approximately 350,000 S2 cells were plated in 24-well plates for 2 h. At 30 min before transfection, 0.6 μg of vector DNA was mixed with 0.06 μg of pCoBlast (Invitrogen), 5 μl of Cellfectin (Invitrogen), and 50 μl of serum-free Schneider medium (SFM) (Sigma). A total of 450 μl of SFM was added to the transfection mix. The medium of the plated S2 cells was removed, and the transfection mix was added. After 3–4 h, 1 ml of serum-containing Schneider medium was added. Cells were incubated for 4 d before adding 25 μg/ml blasticidin. After 3–4 wk, stable cultures were obtained. GFP expression was analysed by western blotting and immunofluorescence (IM). Cells containing the pMT vector were induced 24 h prior to analysis with 100 μM CuSO_4_. When S2 cells were to be analysed by immunofluorescence, cells were plated on glass slides coated with 0.05 μg/ml ConcavalinA (Sigma #C5275) and fixed with 4% paraformaldehyde (freshly prepared in PBS). Cells were costained with anti–α-tubulin (1:1,000 DM1α) and anti-DSas4 antibodies (1:500) and Hoechst. Pictures were taken and analysed as described above. Maximum *z*-projections are shown in all figures.

### Analysis of Cnn phosphorylation.

S2R+ cells incubated with or without dsRNA (as described above) in 24-well plates were washed once with PBS and then suspended in 200 μl of loading buffer. Samples were boiled for 10 min, and 10 μl was loaded on a 3%–8% gels (Nupage; Invitrogen). The samples were blotted on nitrocellulose membranes and probed with anti-Cnn antibodies (1:1,000), as described previously [38]. An anti-actin antibody (MP Biomedicals #08691001) was used as a loading control (1:1,000). For the phosphatase treatment of S2R+ cell extracts, cells were diluted in lysis buffer (PBS, 5 mM EDTA, 1× PMSF, 1× protease inhibitor [Roche Complete]) plus or minus phosphatase inhibitors (25 mM NaF, 1 mM Na_3_VO_4_, 20 mM beta-glycerol phosphate, 1× phosphatase inhibitor cocktail [Sigma #P2850]) and syringed through a G24 needle approximately 60 times on ice. Lysates were incubated for 30 min at 30 °C with 10 units/100 μl of lambda phosphatase (Sigma # P9614). The reaction was quenched by the addition of 4× loading buffer. Samples were analysed by western blotting. For the 2-D analysis, samples were suspended in 2-D buffer (10 mM Tris [pH 8–8.5], 5 mM magnesium acetate, 8 M urea, and 4% CHAPS). The protein concentration was measured and 50 μg of proteins analysed using pH 4–10 strips and 12% acrylamide gels, and processed for western blotting.

### Analysis 3rd instar larval brains.

Third instar larval brains were dissected from wild-type (*w^67^*) and *aurora-A* mutant flies (transheterozygotes between the two hypomorphic alleles *aur^e200^* and *aur^e209^*), and fixed and stained as described previously [16]. Brains were stained with Cnn (1:1,000), α-tubulin (1:1,000), γ-tubulin (1:500), and DSas-4 (1:500) antibodies. More than 50 mitotic cells were analysed for three different brains. For the statistical analysis of the centriole number in these mitotic cells, centrioles were only counted if they were DSas-4 and γ-tubulin positive.

## Supporting Information

Figure S1 Quantitation of PCM Size after Protein Depletion A bar chart showing the average PCM size in S2R+ cells treated with dsRNAs against the proteins identified in our screen. The control PCM size was assigned a value of 100% (GFP—grey), and error bars represent the standard error (SE). Red bars represent genes involved in centriole duplication; green bars represent genes involved in PCM maturation; red/green hybrid bars represent genes involved in both centriole duplication and PCM maturation; dark blue bars represent genes involved in centriole separation. The light blue bars marked with a Δ represent the PCM size in cells depleted of proteins involved in centriole separation, but where we only quantitated the amount of PCM around single centrioles that were well separated from any others. Thus, the depletion of Aurora A (aur) and UbcD6 decreases the amount of PCM recruited around individual centrioles, whereas the depletion of CG14093 does not. Each bar represents the mean intensity of PCM staining (Cnn) from three independent experiments, each analysing more than 20 centrosomes. Error bars represent the SE; a single asterisk (*) or double asterisks (**) indicate *p* ≤ 0.05 or *p* ≤ 0.01 compared to control, respectively. Note that from this experiment, one cannot infer the strength of the defect in PCM recruitment in Cnn-depleted cells, but that Cnn depletion gave an equally strong reduction of the PCM when stained with the PCM markers γ-tubulin and DSpd-2.
(382 KB AI)

Figure S2 A Failure in Centrosome Separation Is the Main Phenotype Observed in *aurora A* Mutant Brain Cells (A–D) Wild-type (*w^67^*) or *aurora-A* mutant (*aur^e200/e209^*) 3rd instar larval brains were stained with anti–α-tubulin (red) and anti-Cnn (green) antibodies and counterstained with Hoechst (blue).
(E) Graph depicting the centriole numbers in mitotic cells in control (grey) and *aurora-A* mutants (red). Centrioles were stained with DSas-4 and were only counted if they where also γ-tubulin positive. More than 50 mitotic cells were counted from three different brains.
(F) Graph representing the number of centrioles per mitotic cell after RNAi in S2R+ cells. Centrioles were stained with DSas-4 antibodies and counted in control (grey) and Aurora-A (red)–depleted cells.
(7.99 MB AI)

Figure S3 Statistical Analysis of the Primary Screen Using CellProfiler and CellHTS (A) A graph showing the “raw” average number of centrosomes (centrosome index) per mitotic cell in each 384-well plate as measured in CellProfiler. Error bars represent the distribution per plate.
(B) A graph showing the “normalised” number of centrosomes per mitotic cell in each 384-well plate. Error bars represent the distribution per plate.
(C) Representation of all positive and negative controls per plate after normalisation. Due to a pipetting error, plate 25 did not contain any positive control.
(D) Representation of the deviation of all positive and negative controls combined from all plates.
(E) Colour representation (blue as negative, red as positive) of plate 8 after normalisation and edge effect correction using the *B*-score method in CellHTS.
(1.55 MB AI)

Figure S4 The Overexpression of Some Centriole Components in S2 cells Produces Extra Cytoplasmic Dots (A) Images of a cell from several stably transfected S2 cell lines overexpressing a GFP-tagged protein (green) from the Ubq promoter (as labelled in each panel) are shown here. Cells were stained with anti–DSas-4 antibodies (red) to reveal the localisation of the endogenous centrioles, and DNA is shown in blue. Note how DCep135 overexpression induces the formation of filaments in the cytoplasm; these filaments were almost always associated with a centriole. The scale bar represents 5 μm
(B) Graph showing the average number of GFP (orange) and DSas-4–positive (brown) dots per cell after the overexpression of various proteins (as indicated on the graph). More than 30 cells were counted in two independent experiments. Error bars represent the SE. Dashed line represented the expected value of two centrosomes per mitotic cell.
(959 KB AI)

Protocol S1 Overview of the Primary and Secondary Screening and GFP-Tagging for Each Gene Identified in the Screen (A) A representative picture from the primary screen using a 20× objective. Colchicine arrested S2R+ cells stained for Cnn (green), p-H3 (red) and DNA (blue).
(B) Detailed RNAi analysis to distinguish between genes involved in centriole duplication and/or centrosome maturation. dsRNA treated S2R+ cells were stained with Cnn, DSas-4, α-tubulin, and Hoechst.
(C) Detailed RNAi analysis in S2R+ cells for the PCM markers DSpd-2, γ-tubulin, and Cnn.
(D) Analysis of the localisation of each protein using GFP-tagging or antibodies in S2 cells and colocalisation with DSas-4 and α-tubulin.
(E) Graph showing the number of centrioles (DSas-4 positive) and PCM dots (Cnn positive) per mitotic cells after treatment with a control (blue) or dsRNA against each gene (red). These data were collected from two independent experiments where cells were stained with Dsas-4, Cnn, α-tubulin, and Hoechst after RNAi; More than 30 centrosome were counted per experiment.
Scale bar in (A) represents 15 μm; the scale bar in (B, C, and D) represents 5 μm.
(12.88 MB PDF)

Table S1 Validation of the Genes Selected in the Primary Screen (75 KB PDF)

Table S2 List of Genes Excluded from the Screen Due to Lack of Cells or the Absence Of Mitotic Cells (34 KB PDF)

Table S3 List of dsRNAs Used in the Secondary Screening (80 KB PDF)

Table S4 List of GFP-Tagged Proteins Analysed (33 KB PDF)

Table S5 List of All the Genes Identified in the Primary Genome-Wide Screen as Being Defective in Centrosome Function (35 KB XLS)

Text S1 CellProfiler Pipeline Used to Identify the Number of Centrosomes per Mitotic Cell (19 KB TXT)

## Abbreviations

dsRNA: double-stranded RNA
GFP: green fluorescent protein
MT: microtubule
PCM: pericentriolar material
PP2A: protein phosphatase 2A
RNAi: RNA interference
