# Supplementary material for: A Genome-Wide RNAi Screen to Dissect Centriole Duplication and Centrosome Maturation in Drosophila
Source: PLoS Biol. 2008 Sep 16;6(9):e224. doi: 10.1371/journal.pbio.0060224 (PMC2535660; doi:10.1371/journal.pbio.0060224)

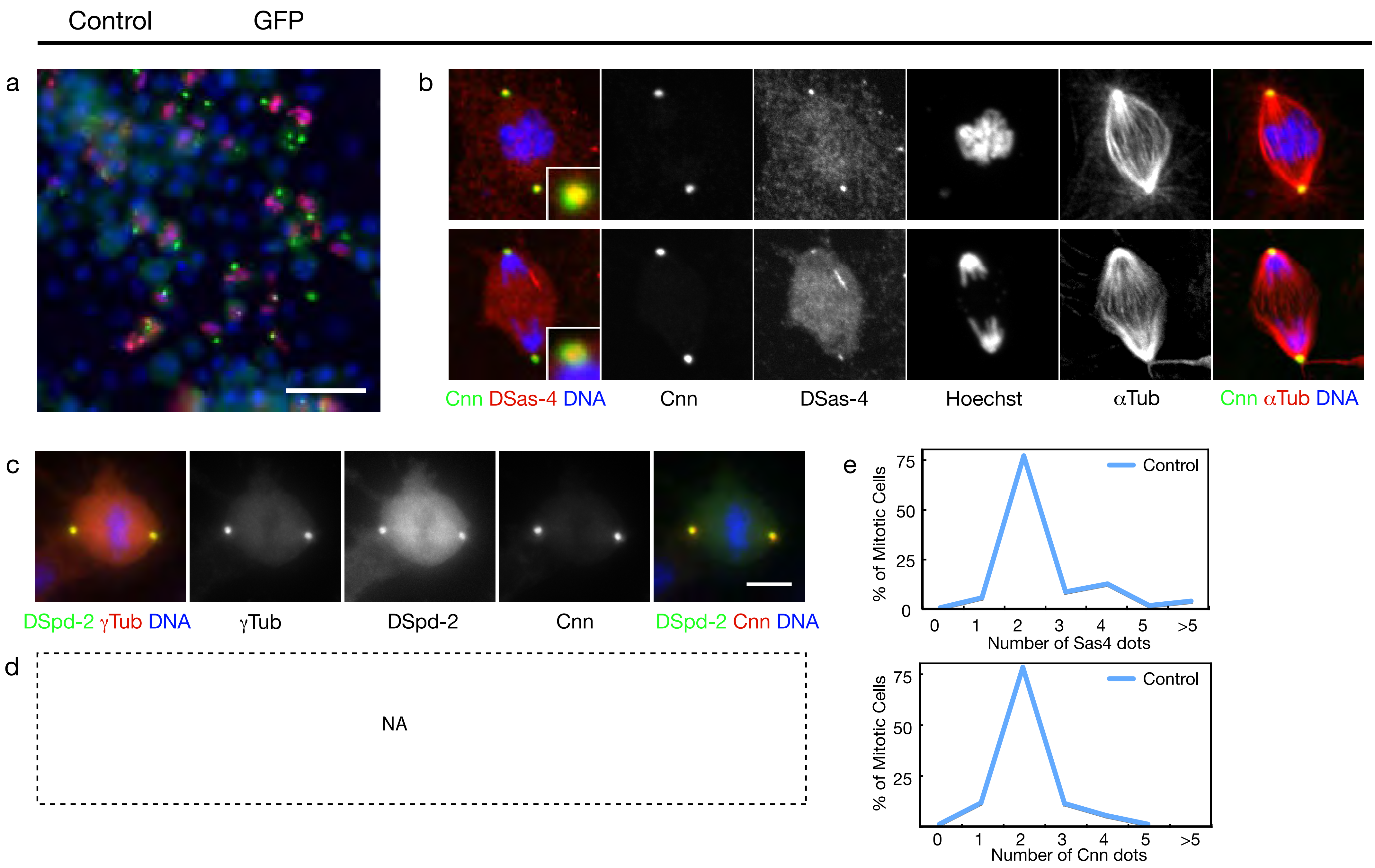
Control
GFP
a
b
Cnn DSas-4 DNA
Cnn
DSas-4
Hoechst
αTub
Cnn αTub DNA
c
DSpd-2 γTub DNA
γTub
DSpd-2
Cnn
DSpd-2 Cnn DNA
d
NA
e
Control
% of Mitotic Cells
75
50
25
0
0 1 2 3 4 5 >5
Number of Sas4 dots
Control
% of Mitotic Cells
75
50
25
0 1 2 3 4 5 >5
Number of Cnn dots

Class I :
Centriole Duplication Defects

Ana3 CG13162

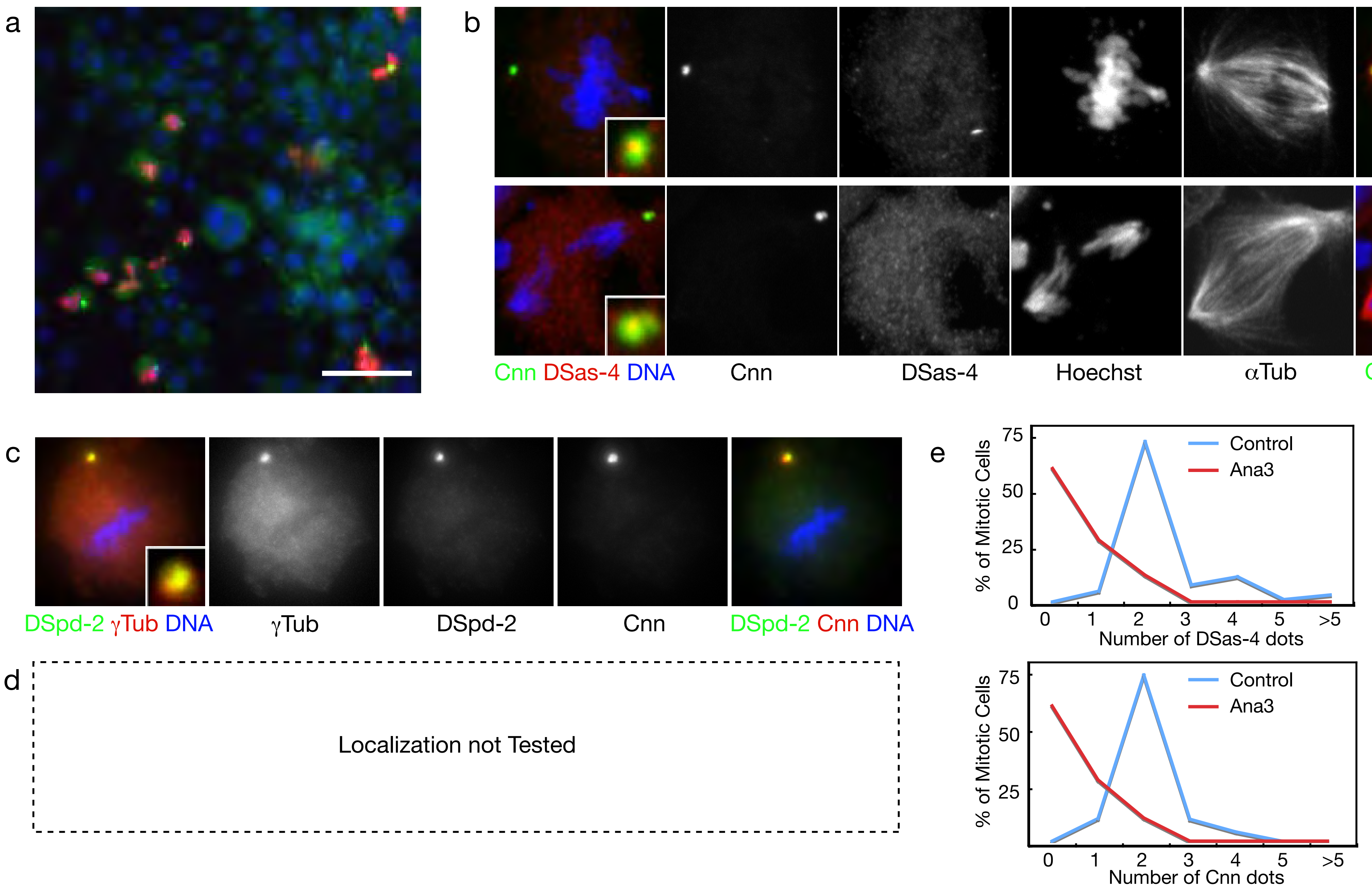

Sak CG7186

a

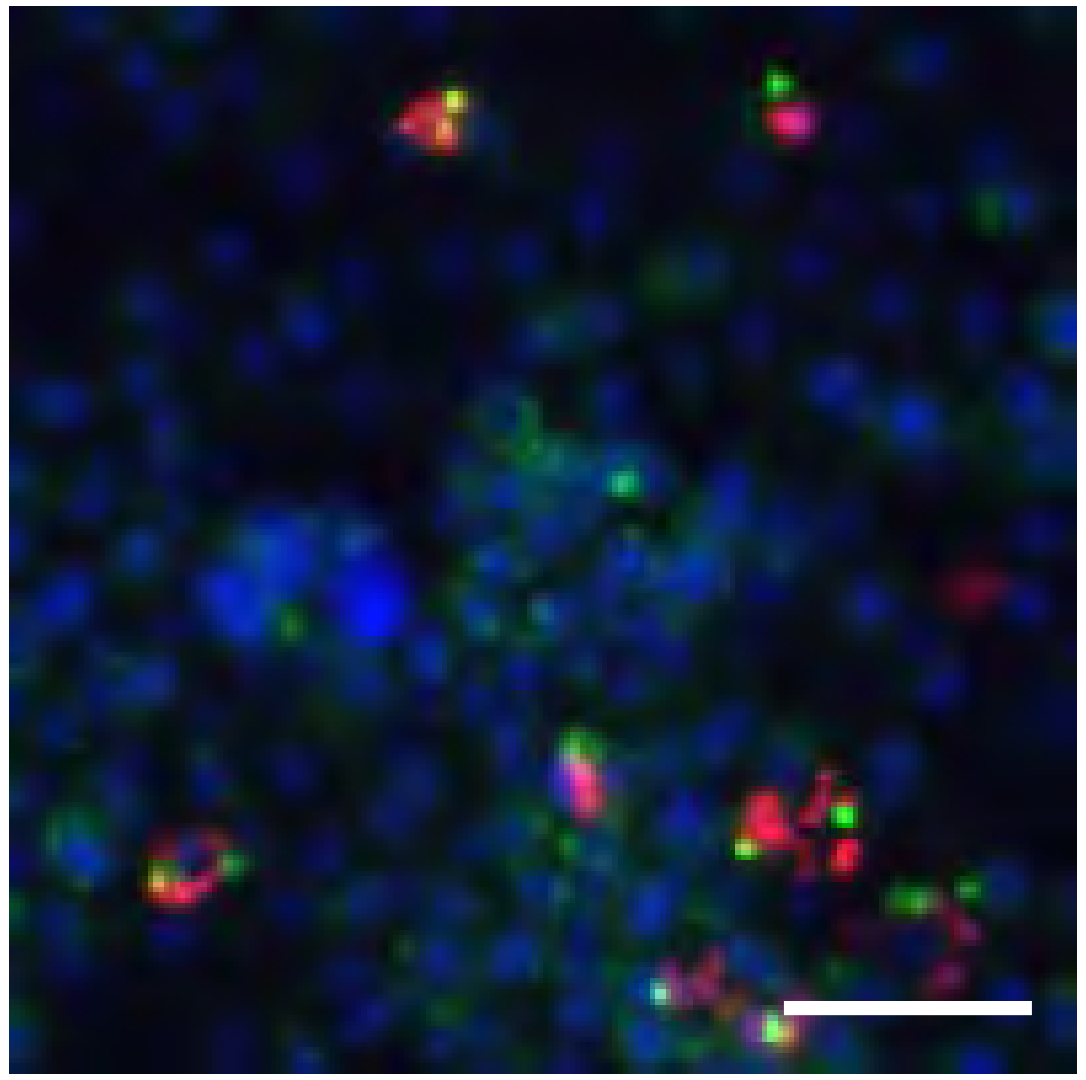

b

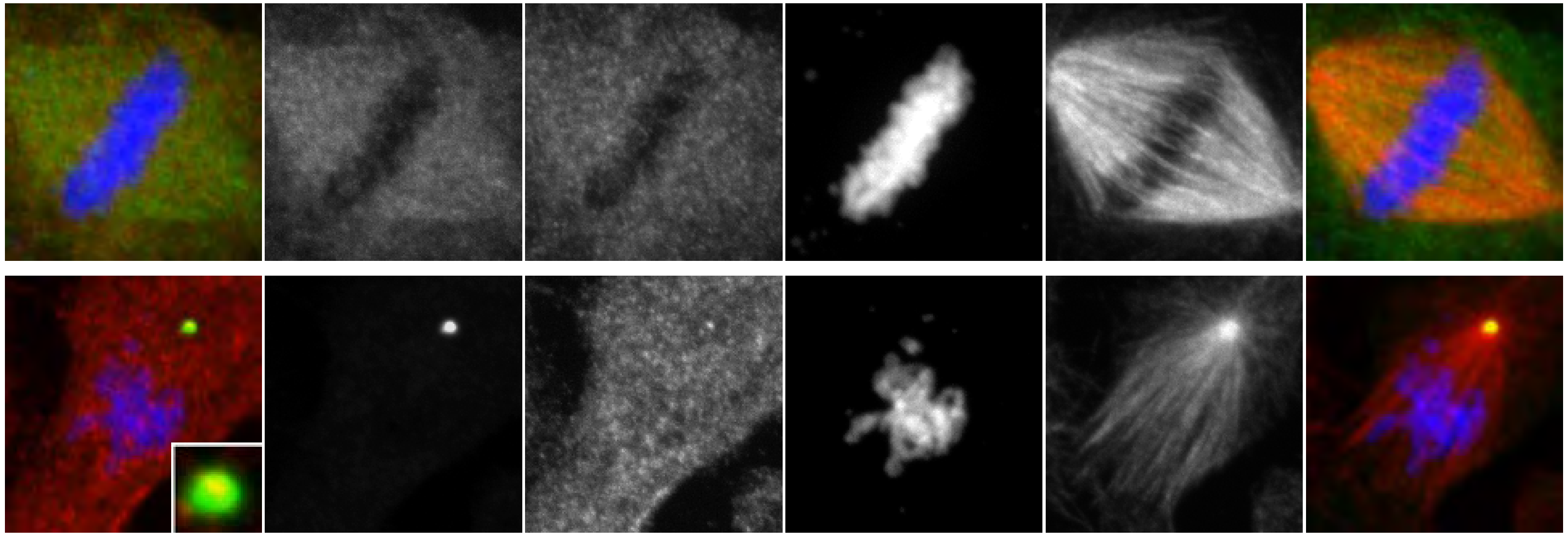

c

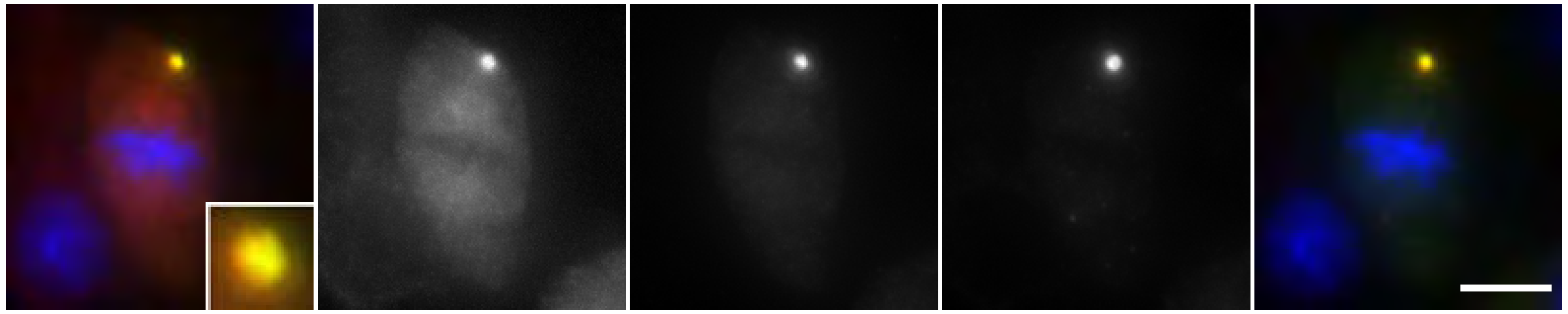

d

Reference:
SAK/PLK4 Is Required for Centriole Duplication and Flagella Development.
Current Biology, Volume 15, Issue 24, Pages 2199-2207, 2005
M. Bettencourt-Dias et al.

e

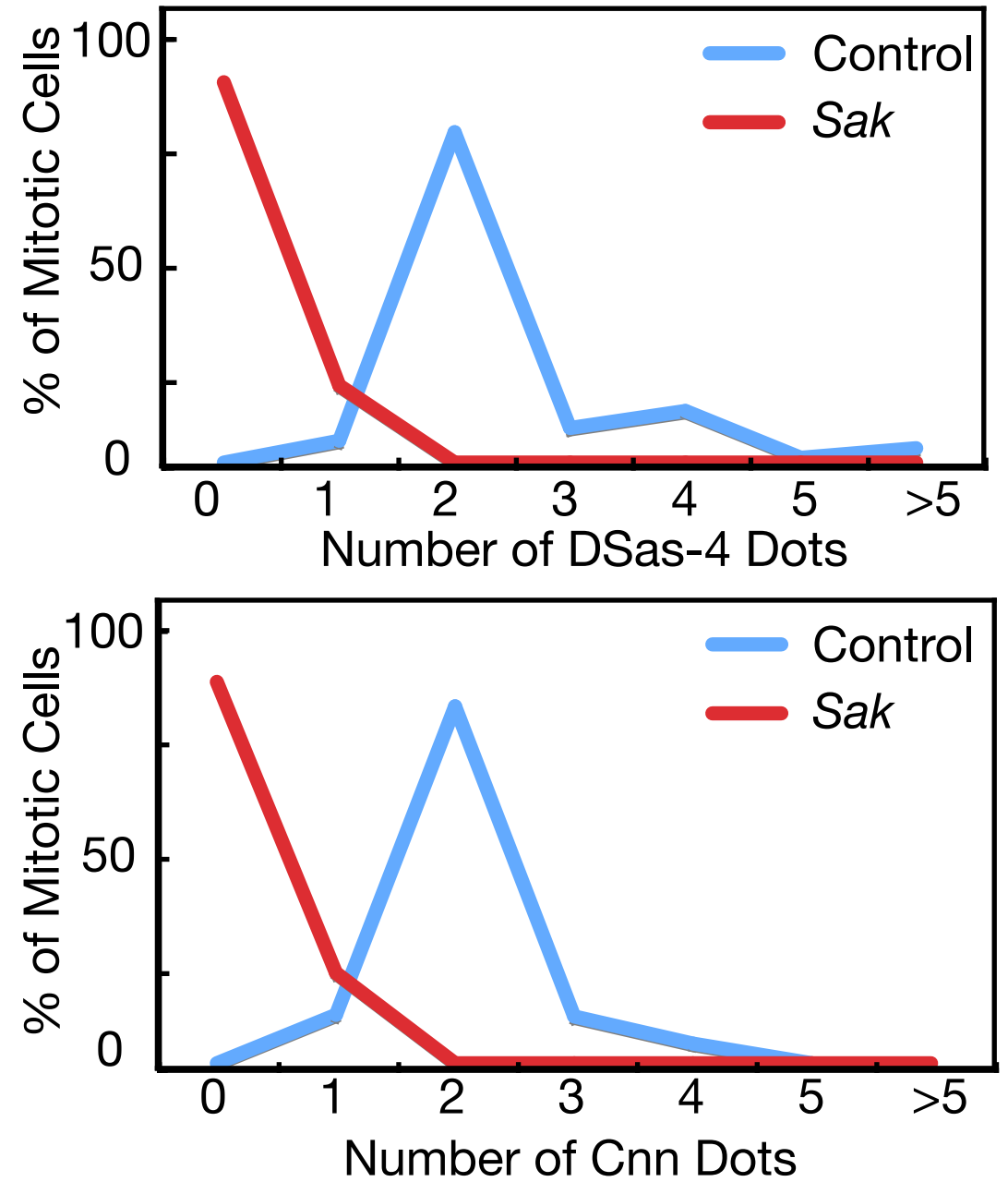

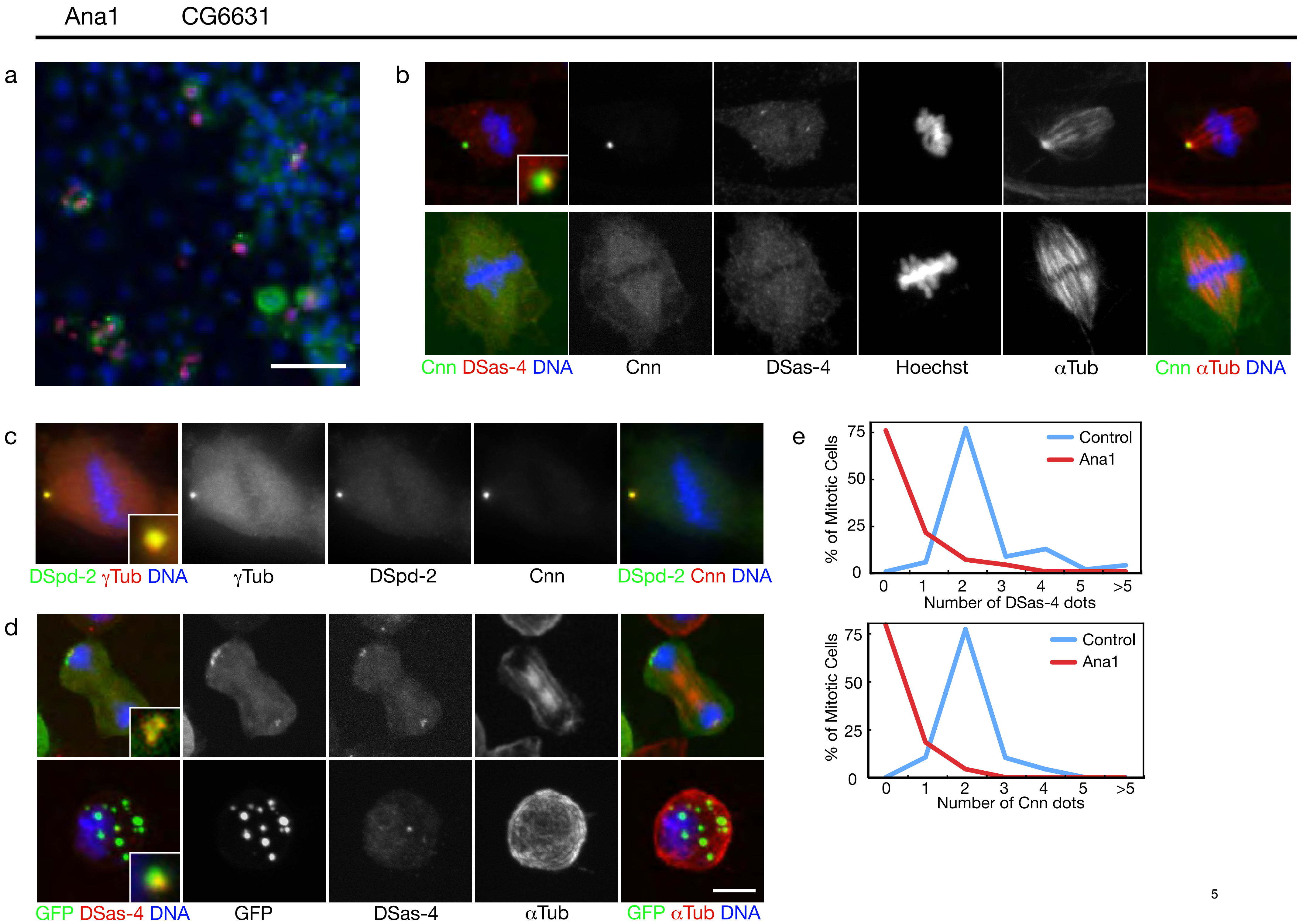
Ana1 CG6631
a
b
Cnn DSas-4 DNA
Cnn
DSas-4
Hoechst
αTub
Cnn αTub DNA
c
DSpd-2 γTub DNA
γTub
DSpd-2
Cnn
DSpd-2 Cnn DNA
d
GFP DSas-4 DNA
GFP
DSas-4
αTub
GFP αTub DNA
e
% of Mitotic Cells
Control
Ana1
Number of DSas-4 dots
Number of Cnn dots
0 25 50 75
0 1 2 3 4 5 >5

Rcd1 CG8233

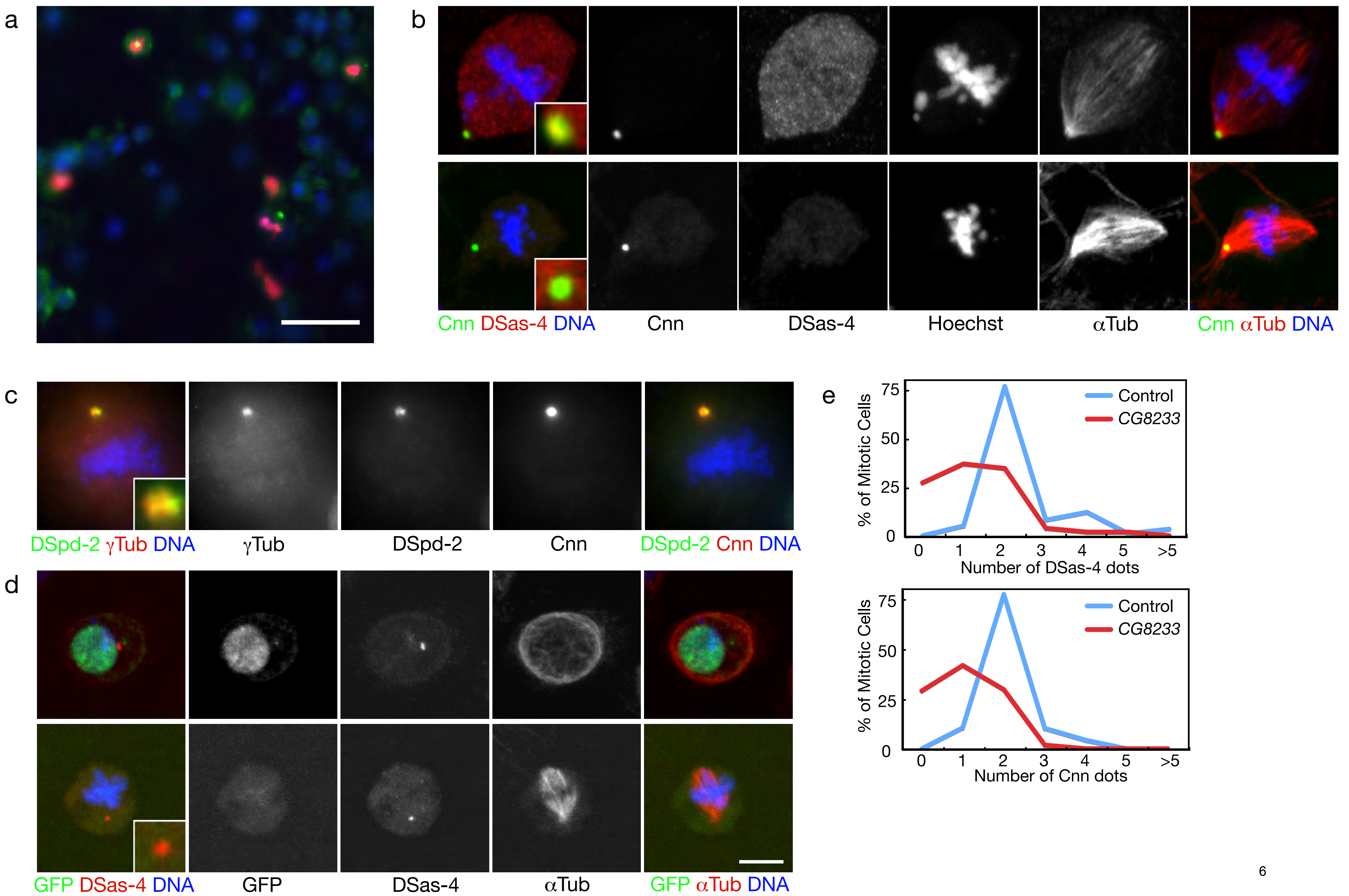

a
b
Cnn DSas-4 DNA
Cnn
DSas-4
Hoechst
αTub
Cnn αTub DNA
c
DSpd-2 γTub DNA
γTub
DSpd-2
Cnn
DSpd-2 Cnn DNA
d
GFP DSas-4 DNA
GFP
DSas-4
αTub
GFP αTub DNA
e
% of Mitotic Cells
75
50
25
0
Control
CG8233
0 1 2 3 4 5 >5
Number of DSas-4 dots
% of Mitotic Cells
75
50
25
0
Control
CG8233
0 1 2 3 4 5 >5
Number of Cnn dots

Rcd2 CG4786

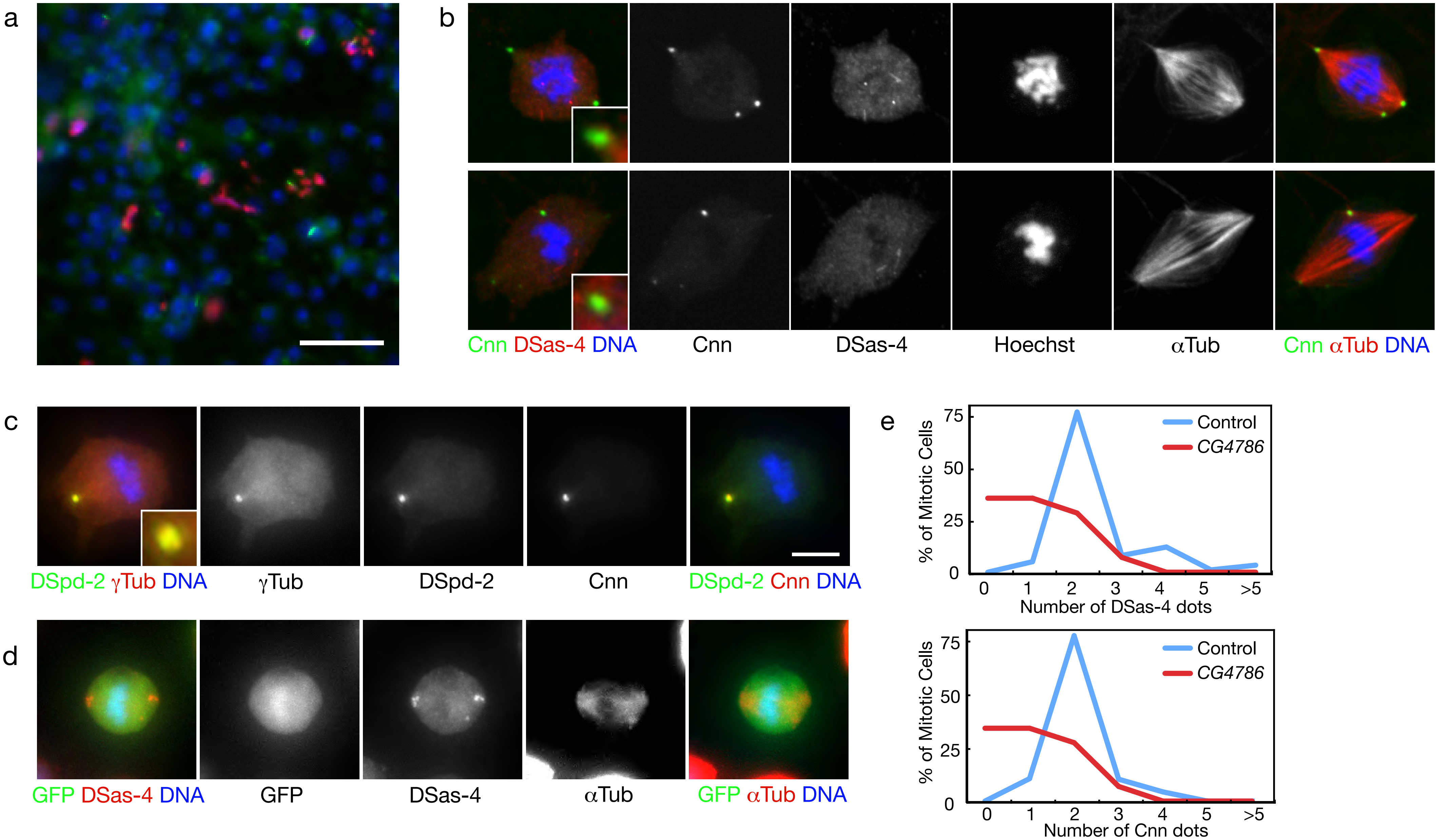
a
b
Cnn DSas-4 DNA
Cnn
DSas-4
Hoechst
αTub
Cnn αTub DNA
c
DSpd-2 γTub DNA
γTub
DSpd-2
Cnn
DSpd-2 Cnn DNA
d
GFP DSas-4 DNA
GFP
DSas-4
αTub
GFP αTub DNA
e
% of Mitotic Cells
Control
CG4786
Number of DSas-4 dots
Number of Cnn dots
0
25
50
75
0 1 2 3 4 5 >5

DSas-6 CG15524

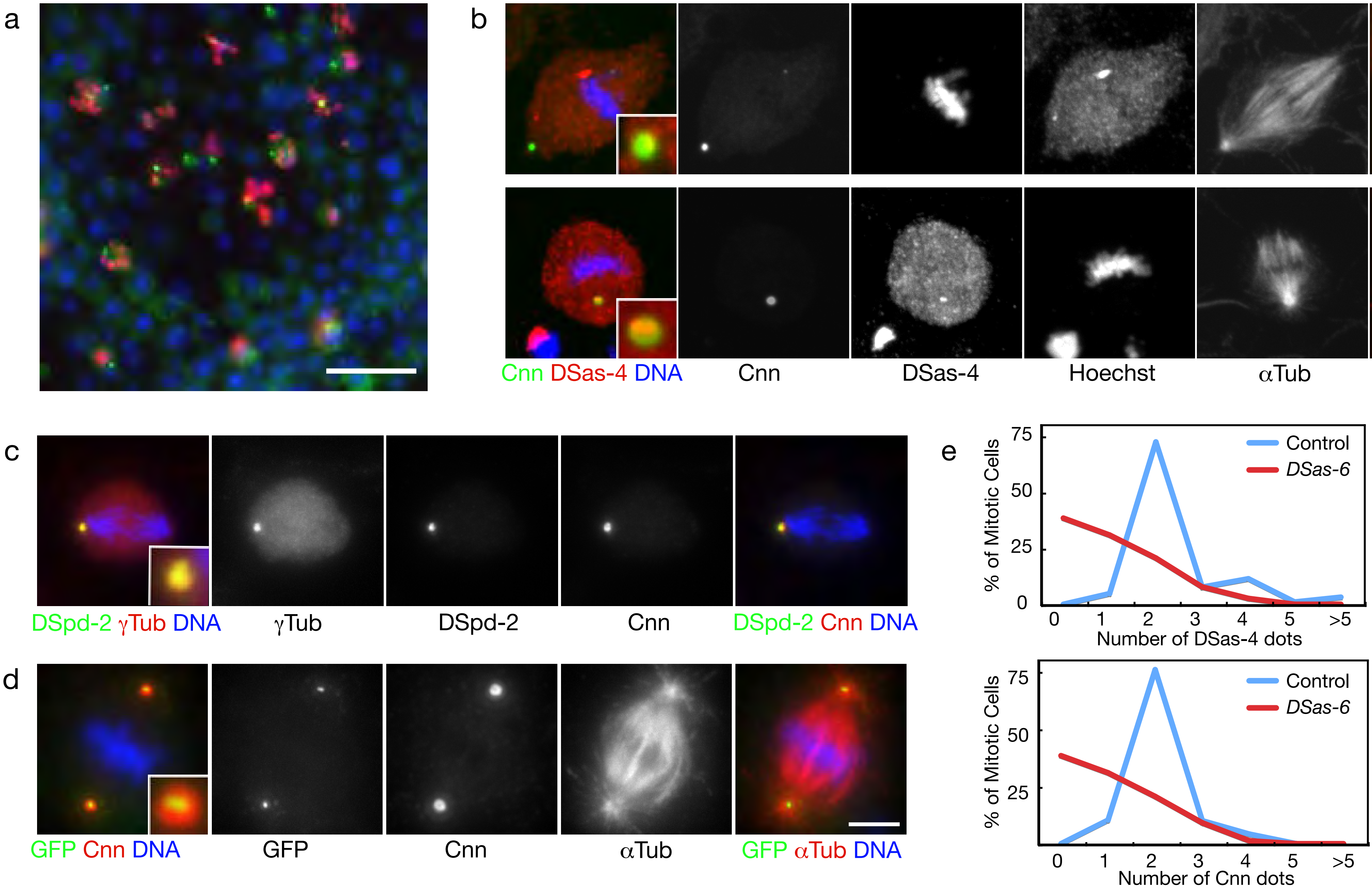

emb CG13387

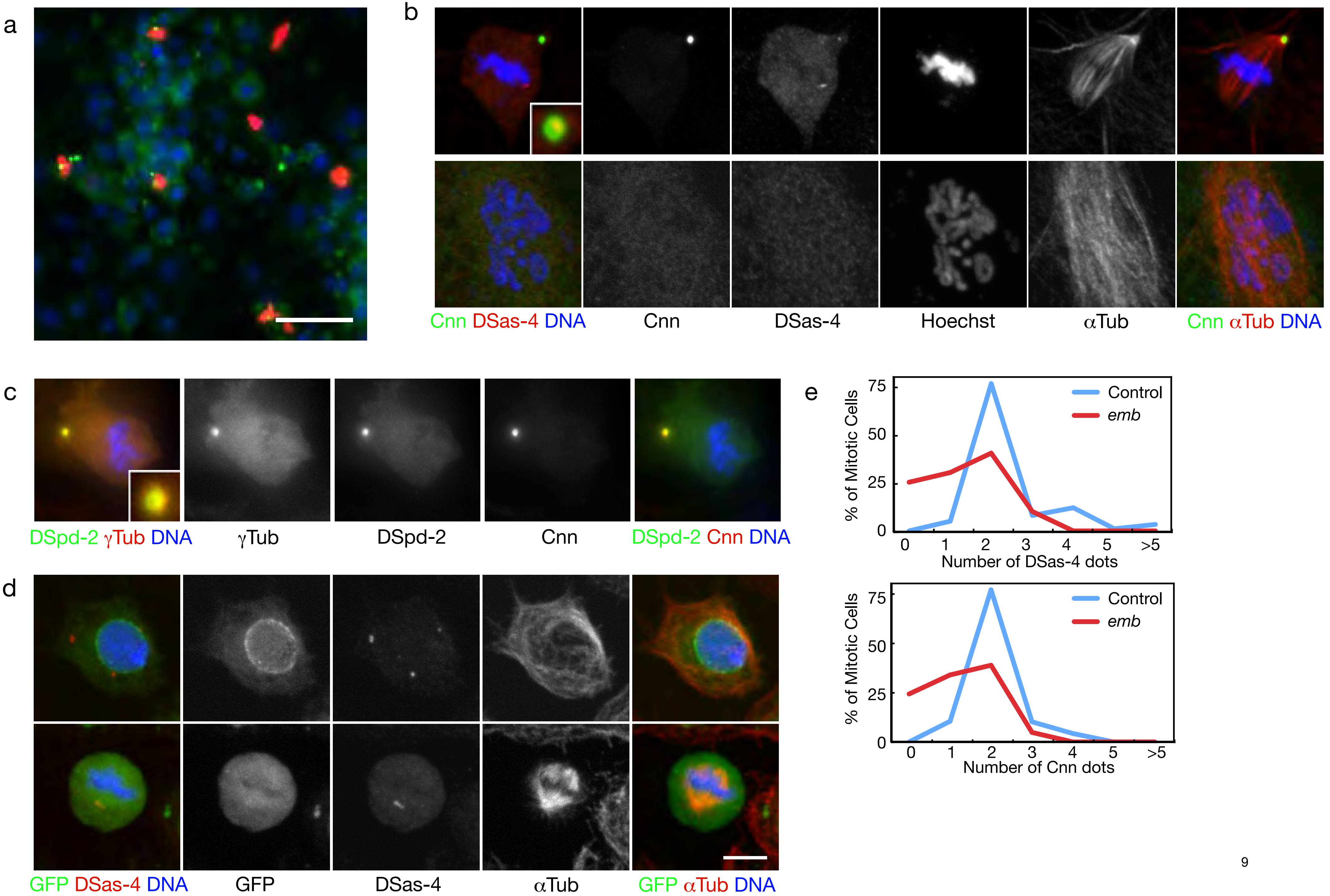
a
b
Cnn DSas-4 DNA
Cnn
DSas-4
Hoechst
αTub
Cnn αTub DNA
c
DSpd-2 γTub DNA
γTub
DSpd-2
Cnn
DSpd-2 Cnn DNA
d
GFP DSas-4 DNA
GFP
DSas-4
αTub
GFP αTub DNA
e
% of Mitotic Cells
Control
emb
Number of DSas-4 dots
Number of Cnn dots
0 1 2 3 4 5 >5
0 25 50 75

DSas-4 CG10061

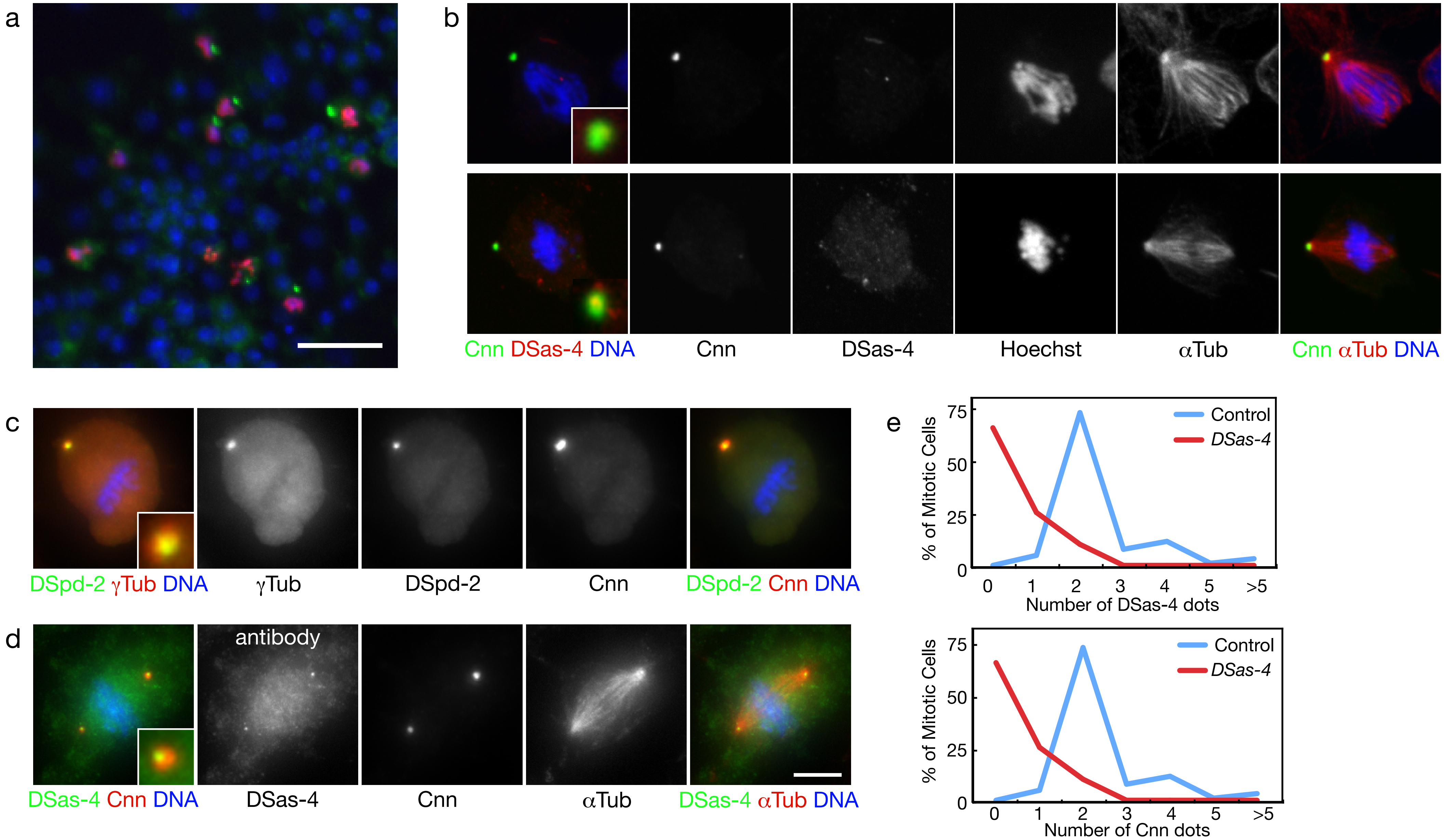

Ana2 CG8262

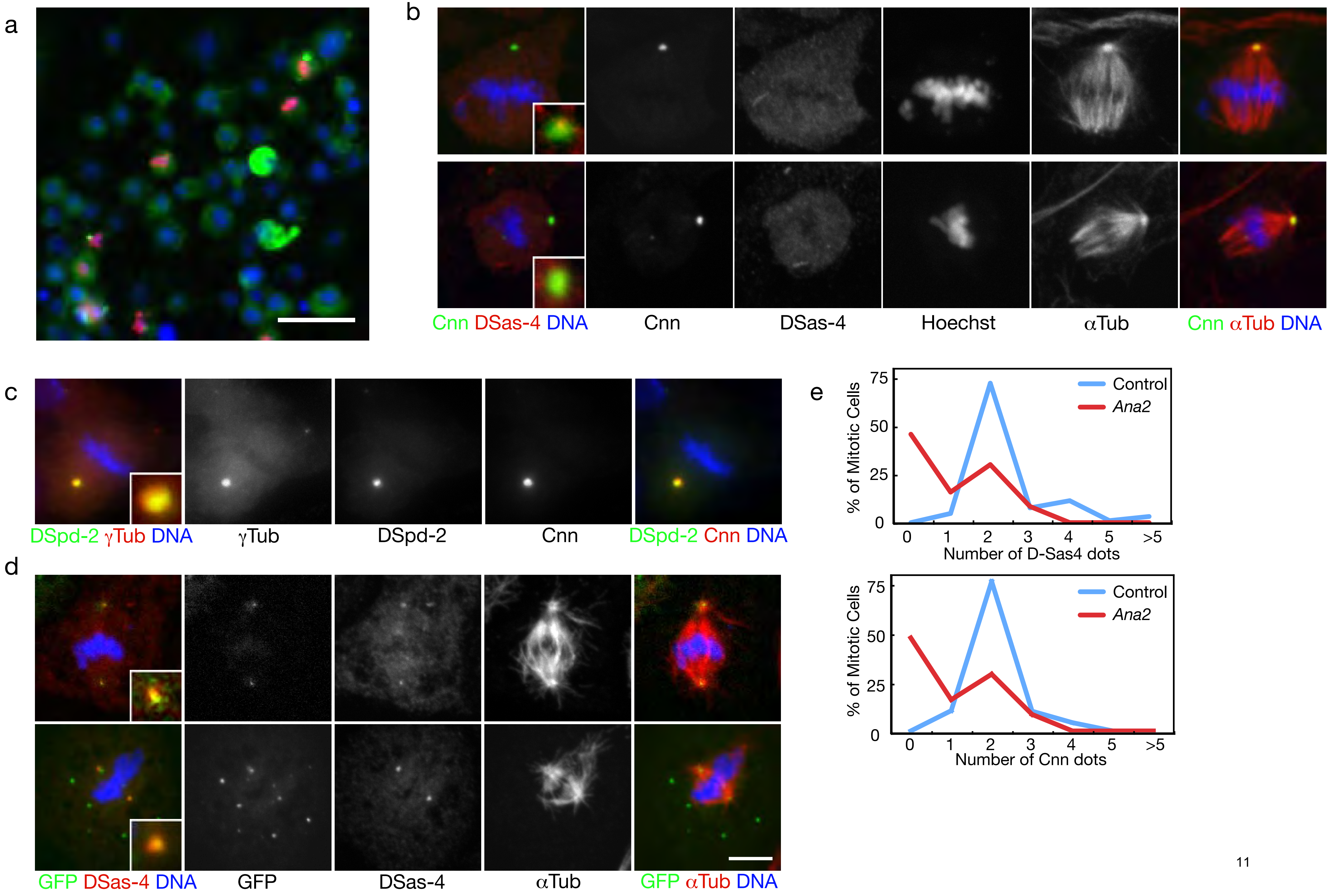
a
b
Cnn DSas-4 DNA
Cnn
DSas-4
Hoechst
αTub
Cnn αTub DNA
c
DSpd-2 γTub DNA
γTub
DSpd-2
Cnn
DSpd-2 Cnn DNA
d
GFP DSas-4 DNA
GFP
DSas-4
αTub
GFP αTub DNA
e
% of Mitotic Cells
75
50
25
0
Control
Ana2
0 1 2 3 4 5 >5
Number of D-Sas4 dots
% of Mitotic Cells
75
50
25
0
Control
Ana2
0 1 2 3 4 5 >5
Number of Cnn dots

Class II :
Centriole Duplication and PCM Maturation Defects

DCep97 CG3980

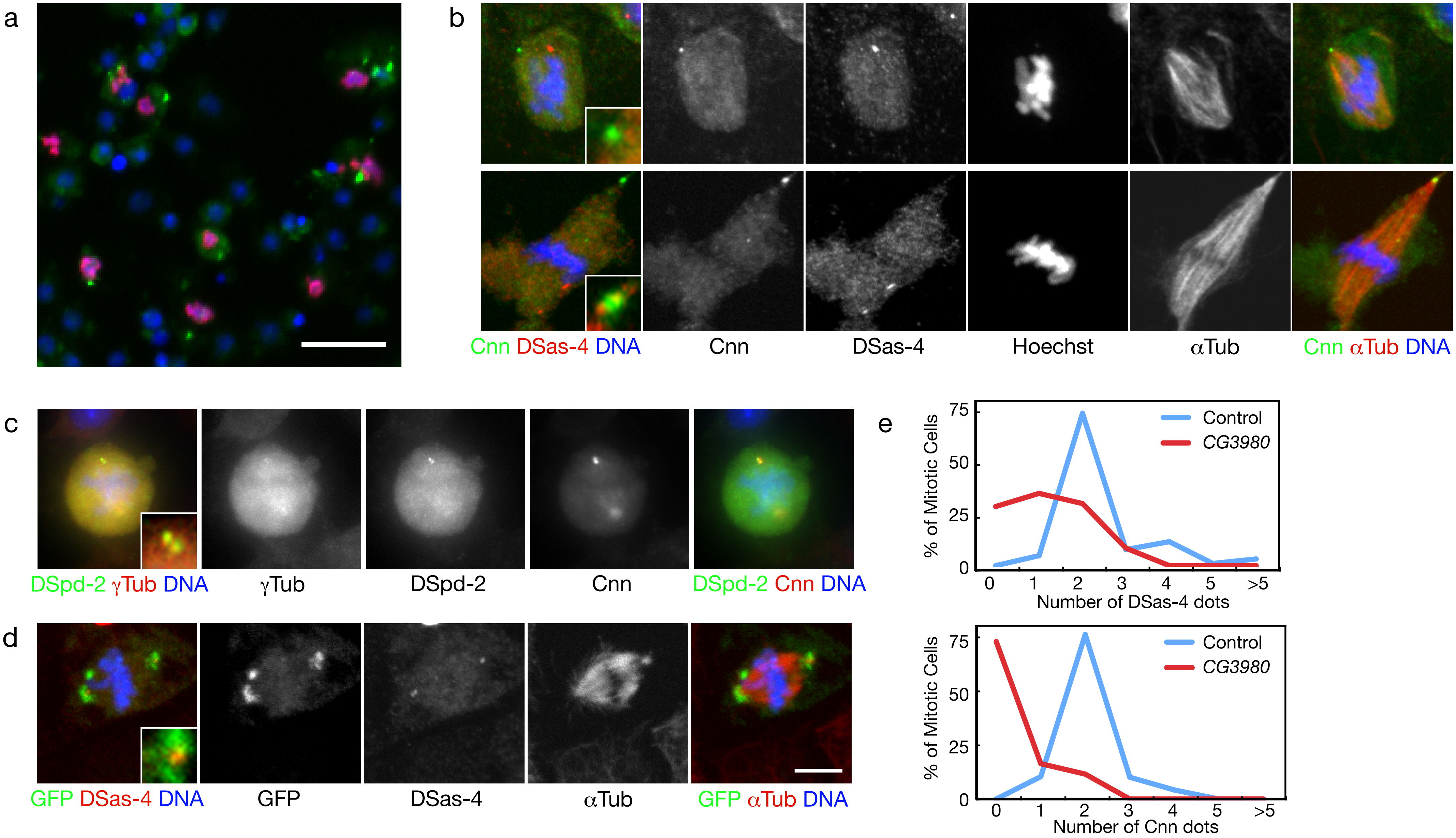

DCp110 CG14617

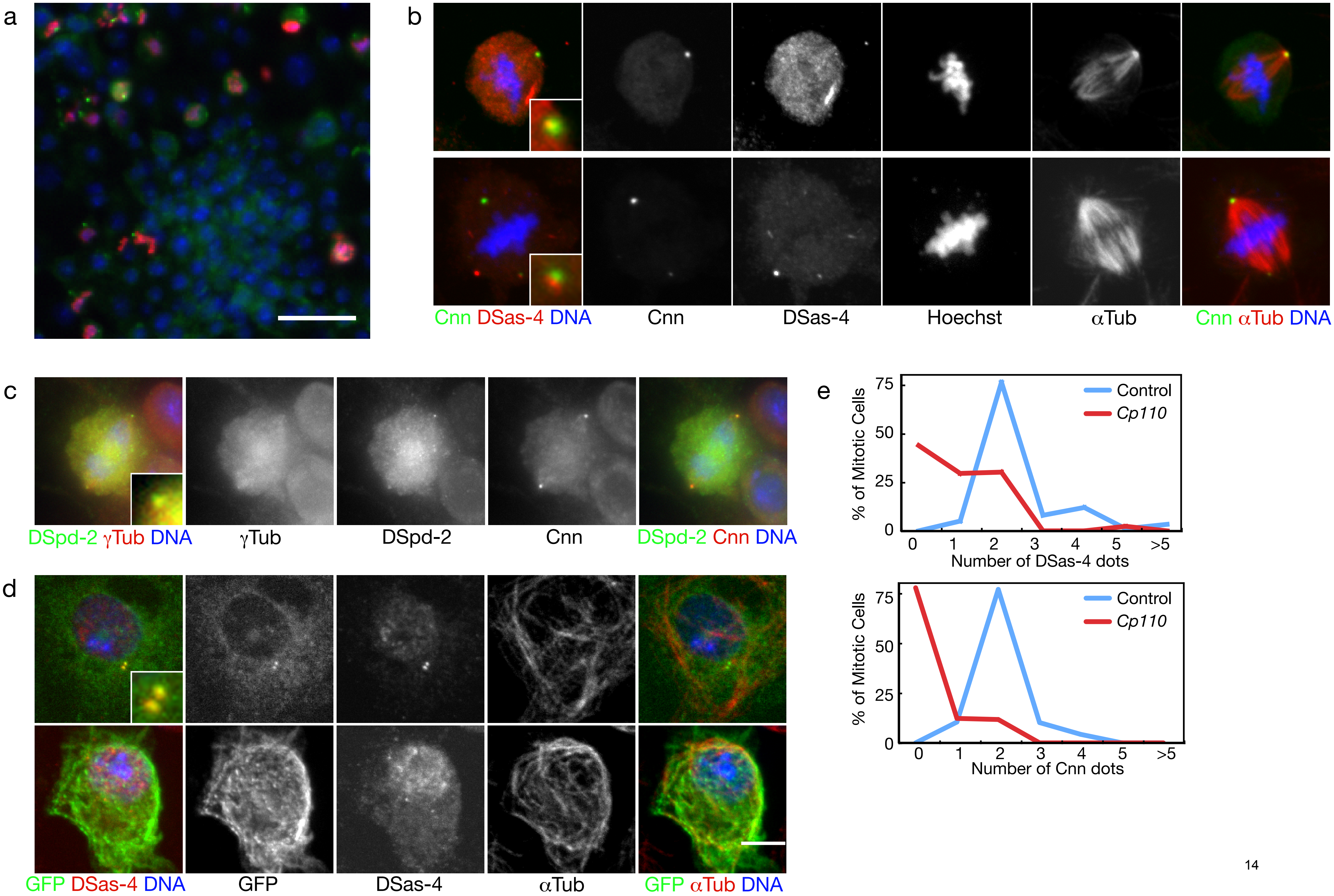

Rcd3 CG8231

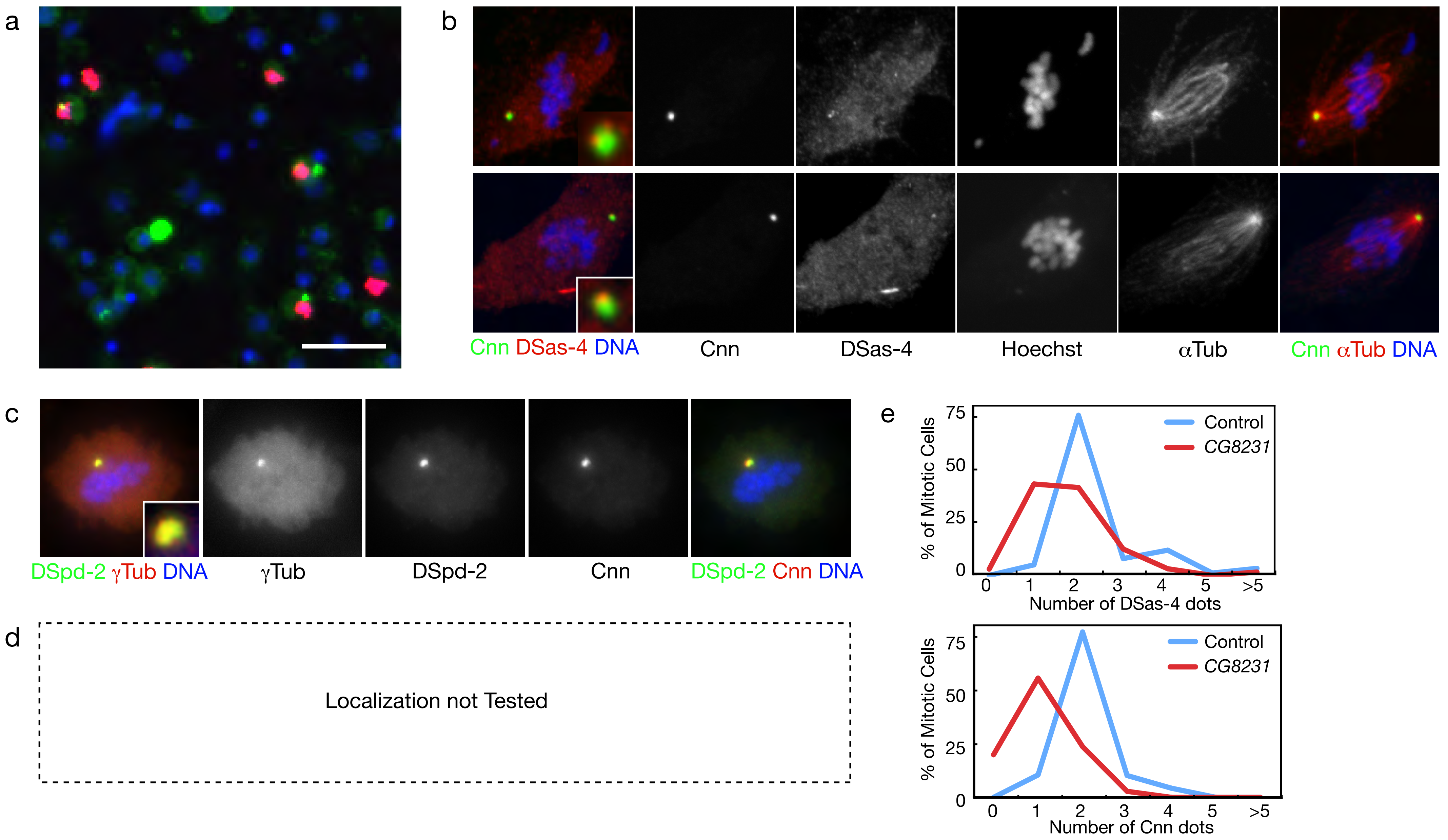
a
b
Cnn DSas-4 DNA
Cnn
DSas-4
Hoechst
αTub
Cnn αTub DNA
c
DSpd-2 γTub DNA
γTub
DSpd-2
Cnn
DSpd-2 Cnn DNA
d
Localization not Tested
e
% of Mitotic Cells
75
50
25
0
Control
CG8231
0 1 2 3 4 5 >5
Number of DSas-4 dots
% of Mitotic Cells
75
50
25
0
Control
CG8231
0 1 2 3 4 5 >5
Number of Cnn dots

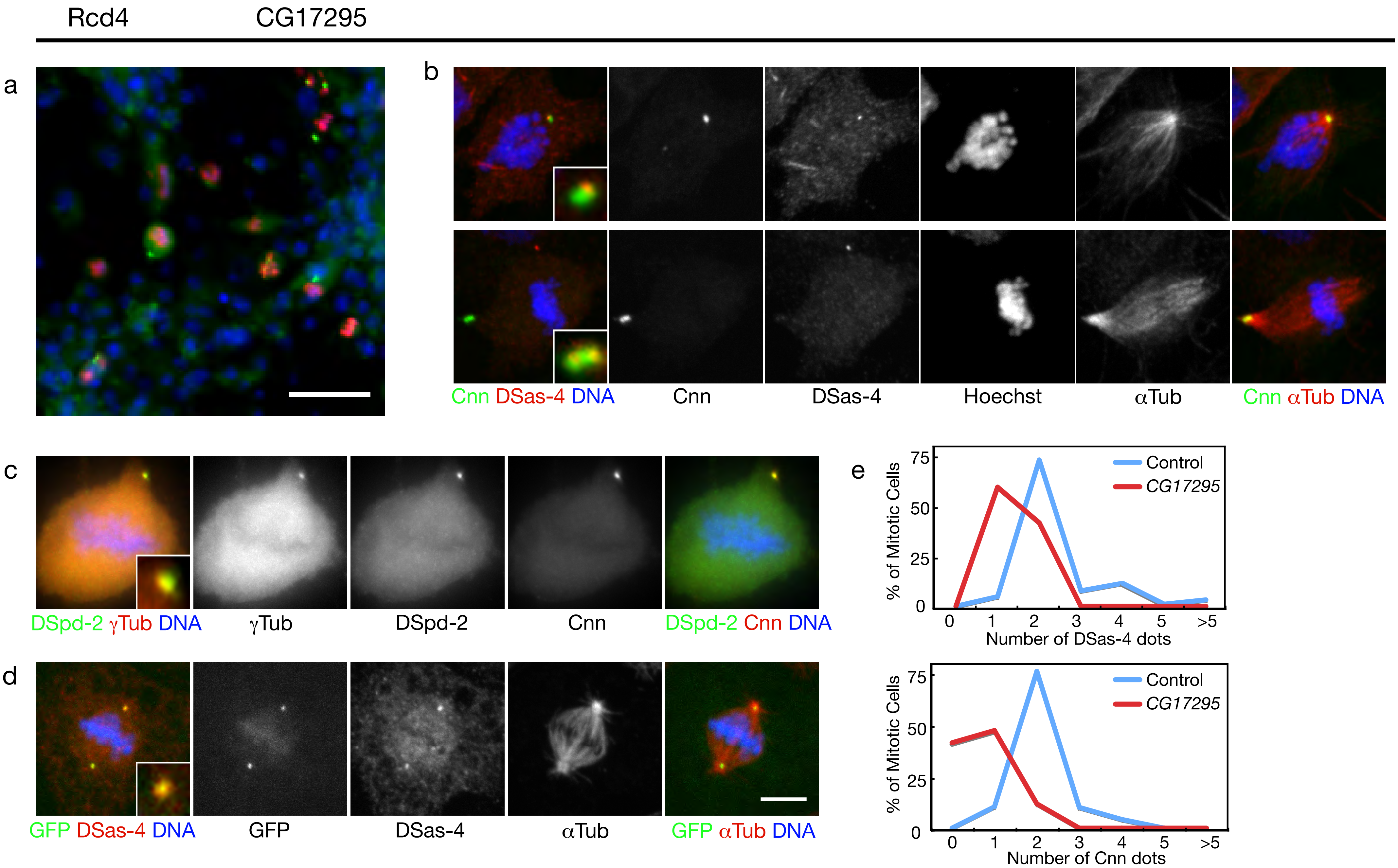

Rcd4
CG17295
a
b
Cnn DSas-4 DNA
Cnn
DSas-4
Hoechst
αTub
Cnn αTub DNA
c
DSpd-2 γTub DNA
γTub
DSpd-2
Cnn
DSpd-2 Cnn DNA
d
GFP DSas-4 DNA
GFP
DSas-4
αTub
GFP αTub DNA
e
% of Mitotic Cells
75
50
25
0
Control
CG17295
0
1
2
3
4
5
>5
Number of DSas-4 dots
% of Mitotic Cells
75
50
25
0
Control
CG17295
0
1
2
3
4
5
>5
Number of Cnn dots

asl CG2919

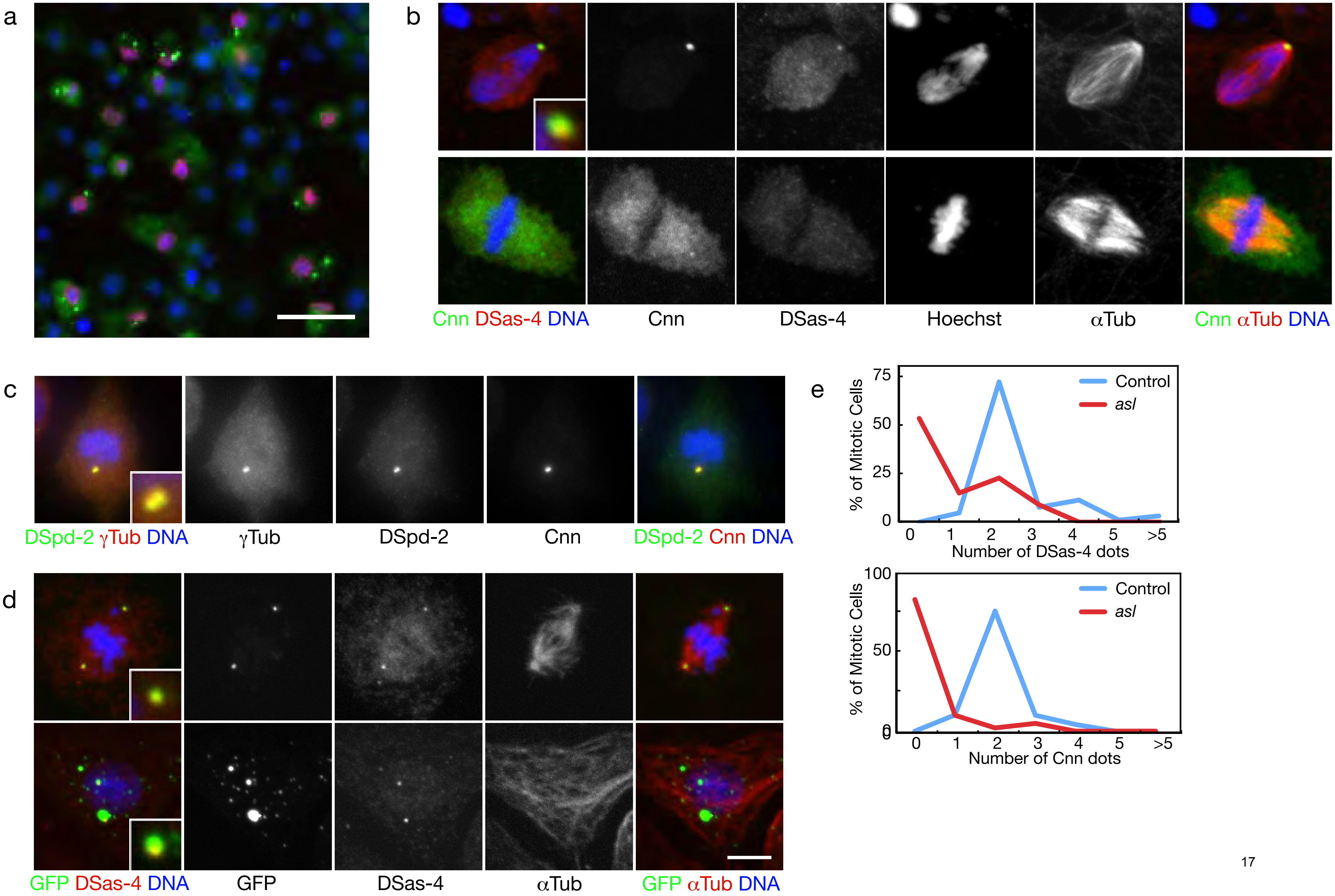
a
b
Cnn DSas-4 DNA
Cnn
DSas-4
Hoechst
αTub
Cnn αTub DNA
c
DSpd-2 γTub DNA
γTub
DSpd-2
Cnn
DSpd-2 Cnn DNA
d
GFP DSas-4 DNA
GFP
DSas-4
αTub
GFP αTub DNA
e
% of Mitotic Cells
Control
asl
Number of DSas-4 dots
0 1 2 3 4 5 >5
0 25 50 75
% of Mitotic Cells
Control
asl
Number of Cnn dots
0 1 2 3 4 5 >5
0 50 100

DCep135 CG17081

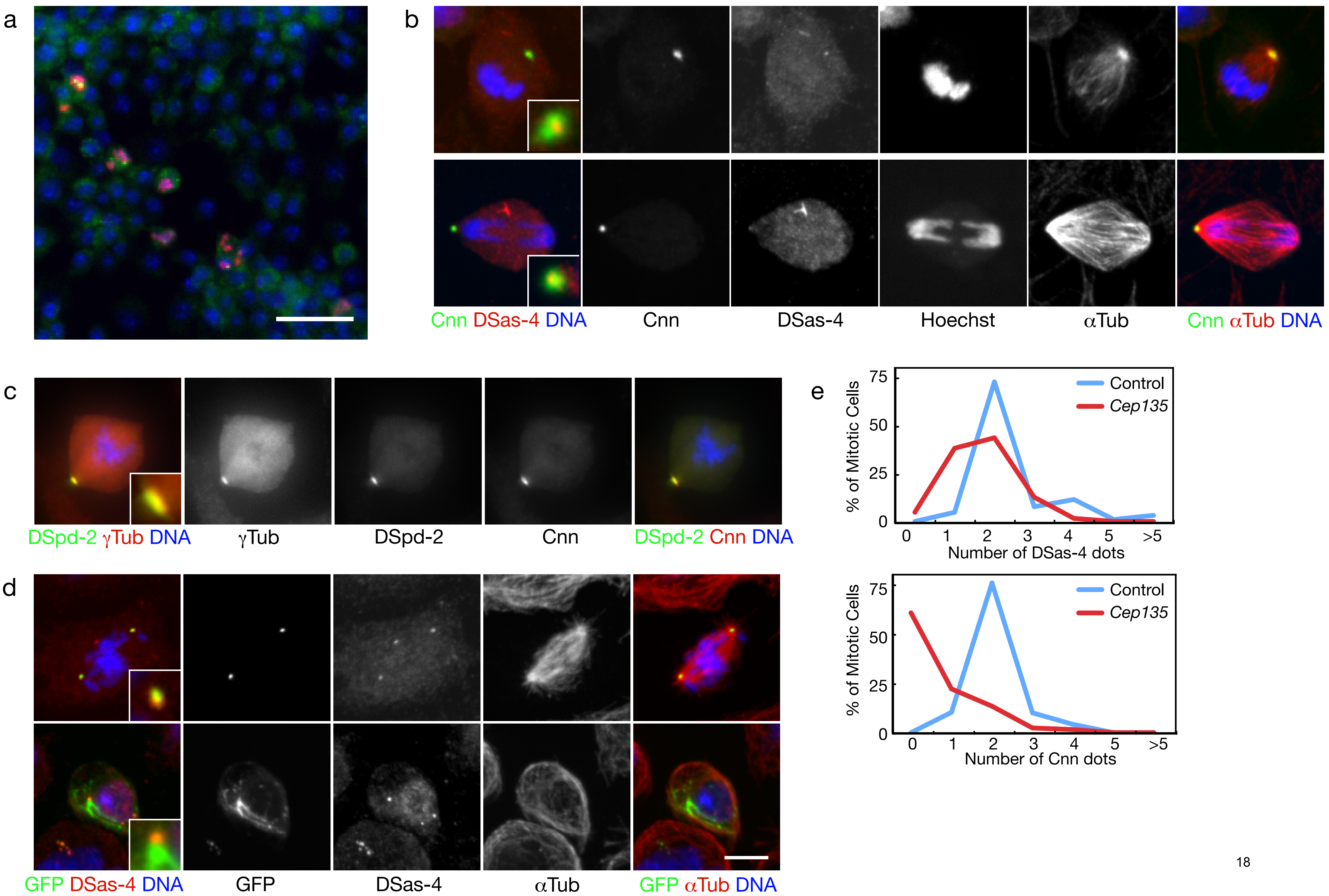

Rcd5 CG1135

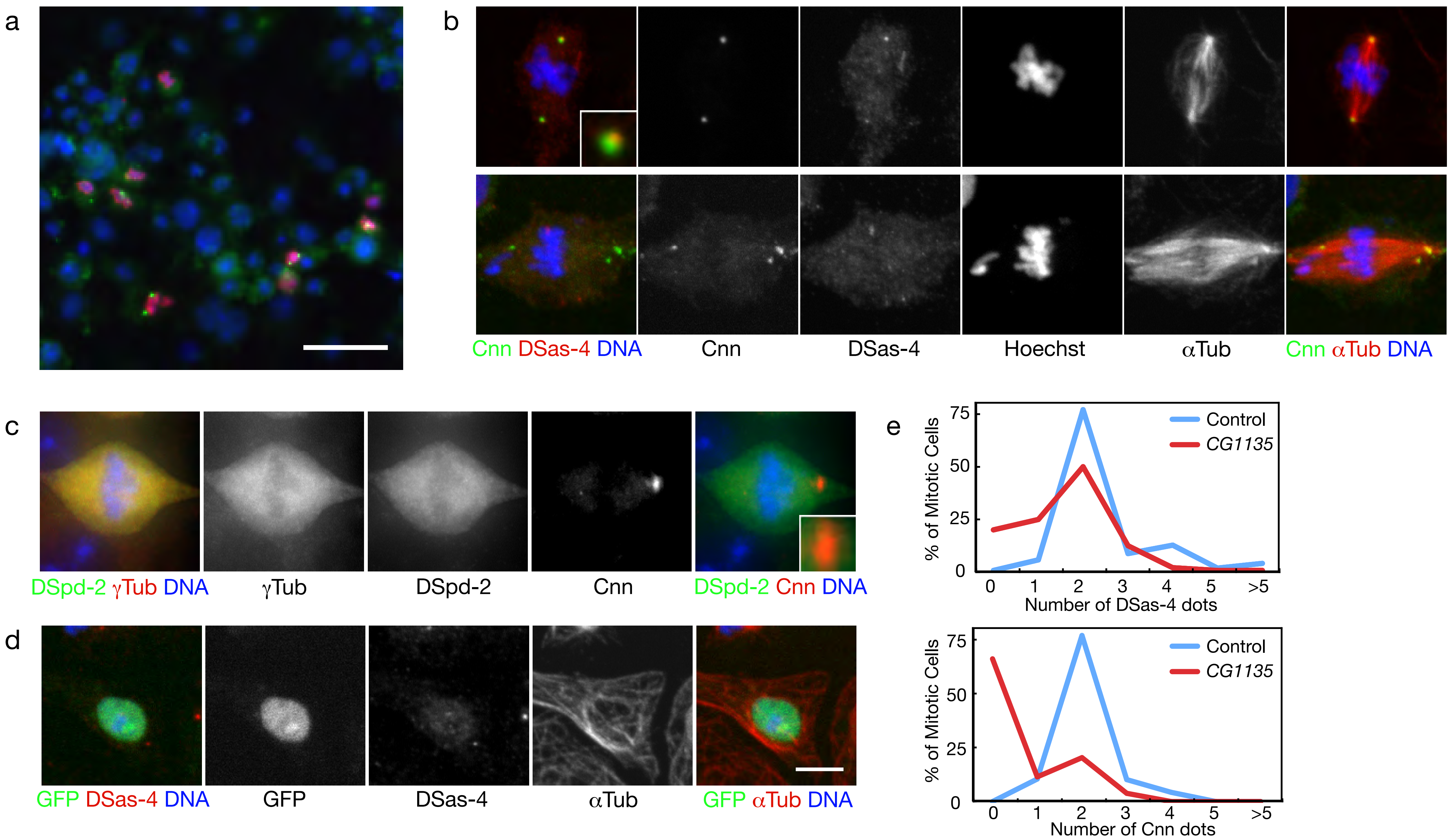

a
b
Cnn DSas-4 DNA
Cnn
DSas-4
Hoechst
αTub
Cnn αTub DNA
c
DSpd-2 γTub DNA
γTub
DSpd-2
Cnn
DSpd-2 Cnn DNA
d
GFP DSas-4 DNA
GFP
DSas-4
αTub
GFP αTub DNA
e
% of Mitotic Cells
75
50
25
0
Control
CG1135
0 1 2 3 4 5 >5
Number of DSas-4 dots
% of Mitotic Cells
75
50
25
0
Control
CG1135
0 1 2 3 4 5 >5
Number of Cnn dots

Myb CG11052

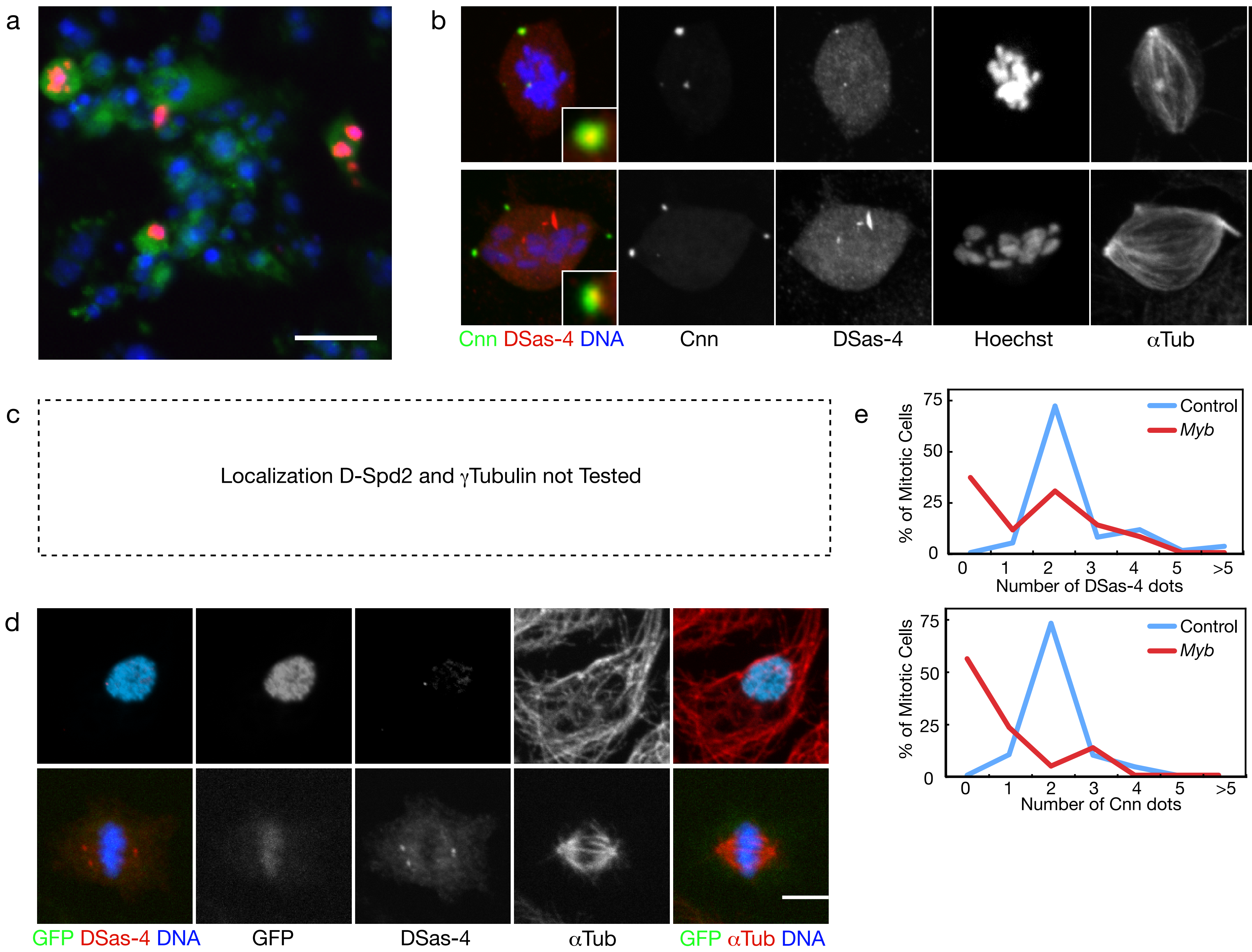
a
b
Cnn DSas-4 DNA
Cnn
DSas-4
Hoechst
αTub
Cnn αTub DNA
c
Localization D-Spd2 and γTubulin not Tested
d
GFP DSas-4 DNA
GFP
DSas-4
αTub
GFP αTub DNA
e
Control
Myb
% of Mitotic Cells
75
50
25
0
0 1 2 3 4 5 >5
Number of DSas-4 dots
Control
Myb
% of Mitotic Cells
75
50
25
0
0 1 2 3 4 5 >5
Number of Cnn dots

Cam CG8472

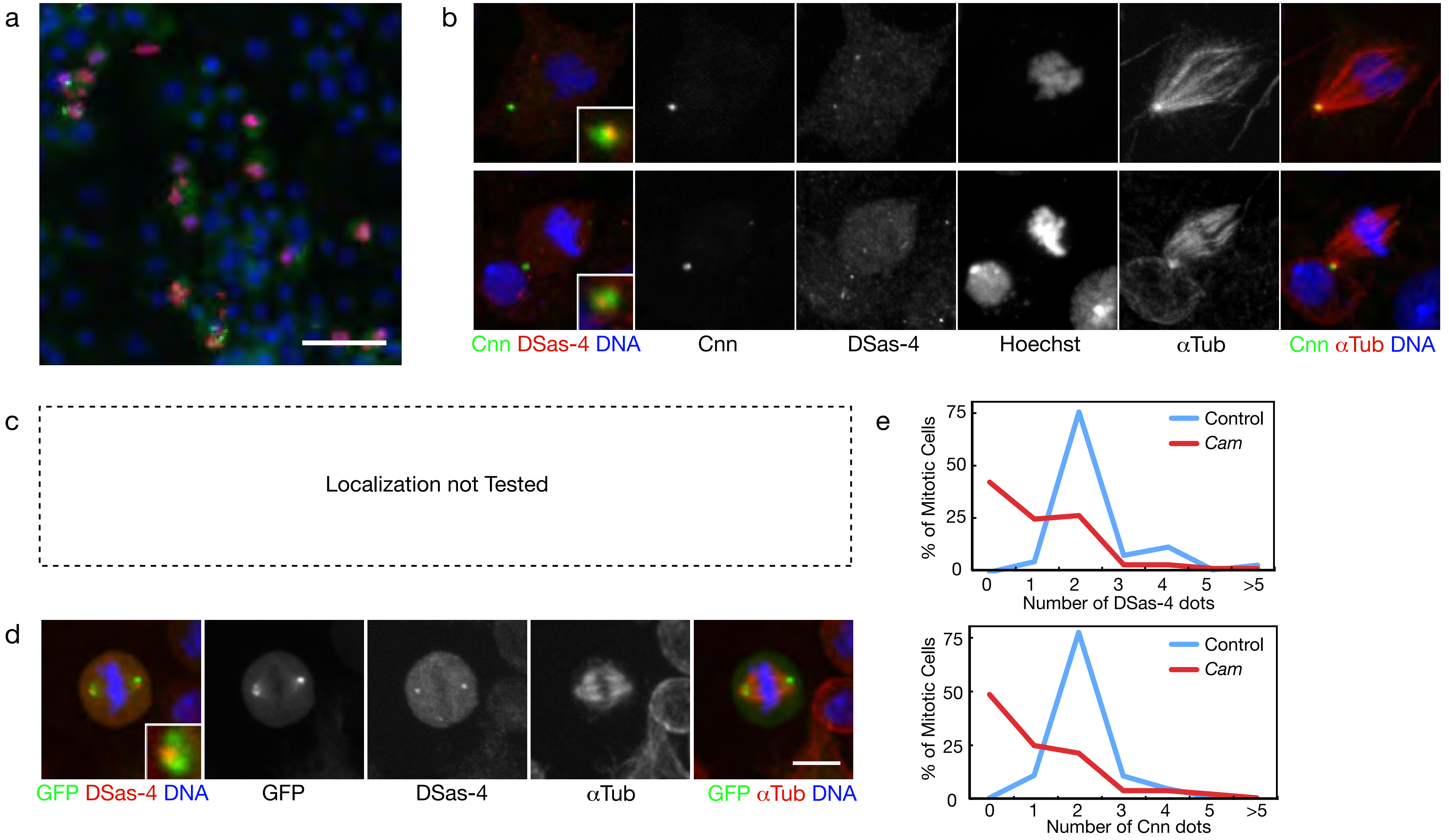
a
b
Cnn DSas-4 DNA
Cnn
DSas-4
Hoechst
αTub
Cnn αTub DNA
c
Localization not Tested
d
GFP DSas-4 DNA
GFP
DSas-4
αTub
GFP αTub DNA
e
% of Mitotic Cells
Control
Cam
Number of DSas-4 dots
Number of Cnn dots
0 1 2 3 4 5 >5
0 25 50 75

Class III :
PCM Maturation Defects

polo CG12306

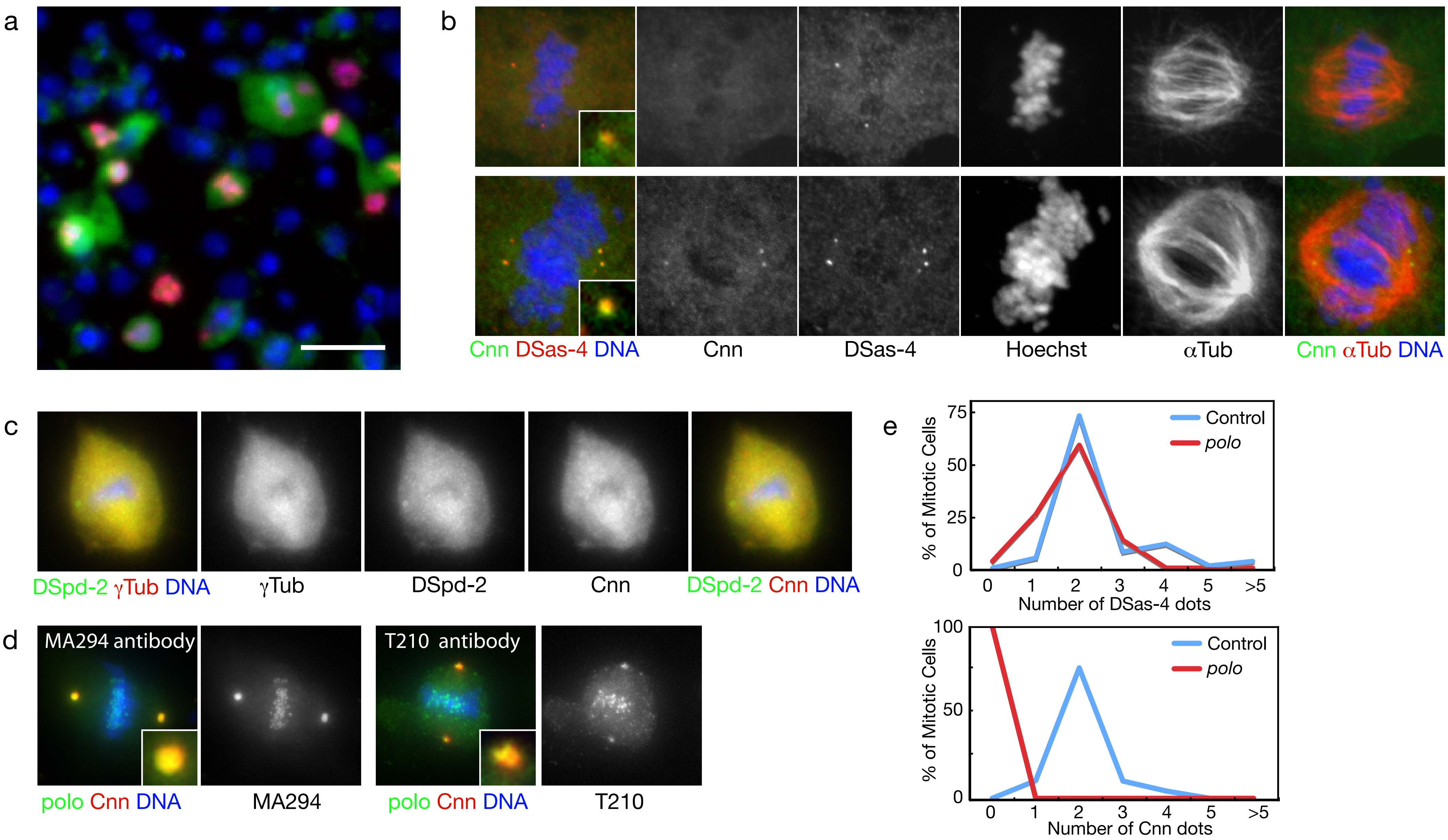

Cnn CG4832

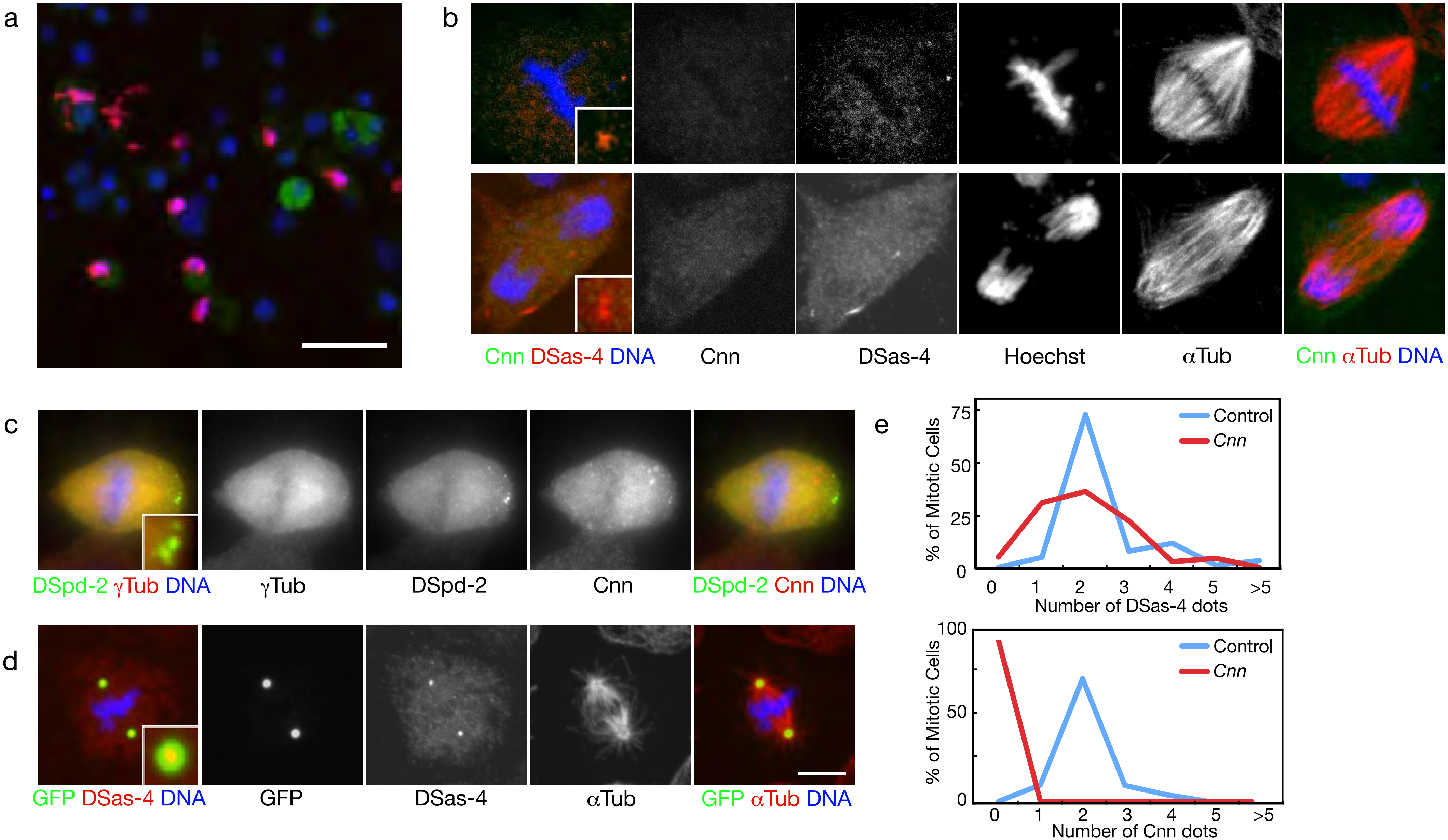
a
b
Cnn DSas-4 DNA
Cnn
DSas-4
Hoechst
αTub
Cnn αTub DNA
c
DSpd-2 γTub DNA
γTub
DSpd-2
Cnn
DSpd-2 Cnn DNA
d
GFP DSas-4 DNA
GFP
DSas-4
αTub
GFP αTub DNA
e
% of Mitotic Cells
75
50
25
0
Control
Cnn
0 1 2 3 4 5 >5
Number of DSas-4 dots
100
50
0
Control
Cnn
0 1 2 3 4 5 >5
Number of Cnn dots

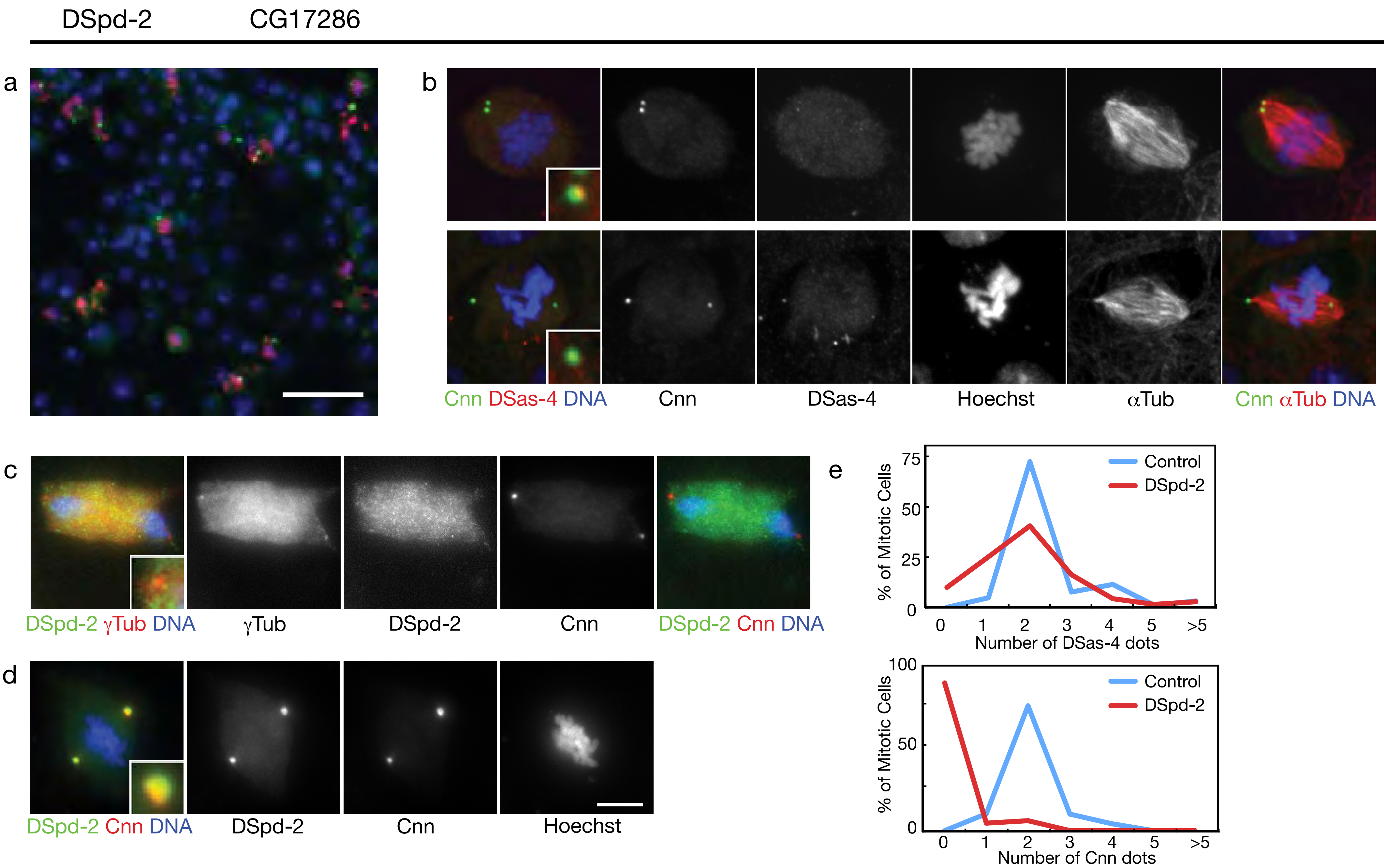
DSpd-2
CG17286
a
b
Cnn DSas-4 DNA
Cnn
DSas-4
Hoechst
αTub
Cnn αTub DNA
c
DSpd-2 γTub DNA
γTub
DSpd-2
Cnn
DSpd-2 Cnn DNA
d
DSpd-2 Cnn DNA
DSpd-2
Cnn
Hoechst
e
% of Mitotic Cells
Control
DSpd-2
Number of DSas-4 dots
Number of Cnn dots
0
1
2
3
4
5
>5
25
50
75
100

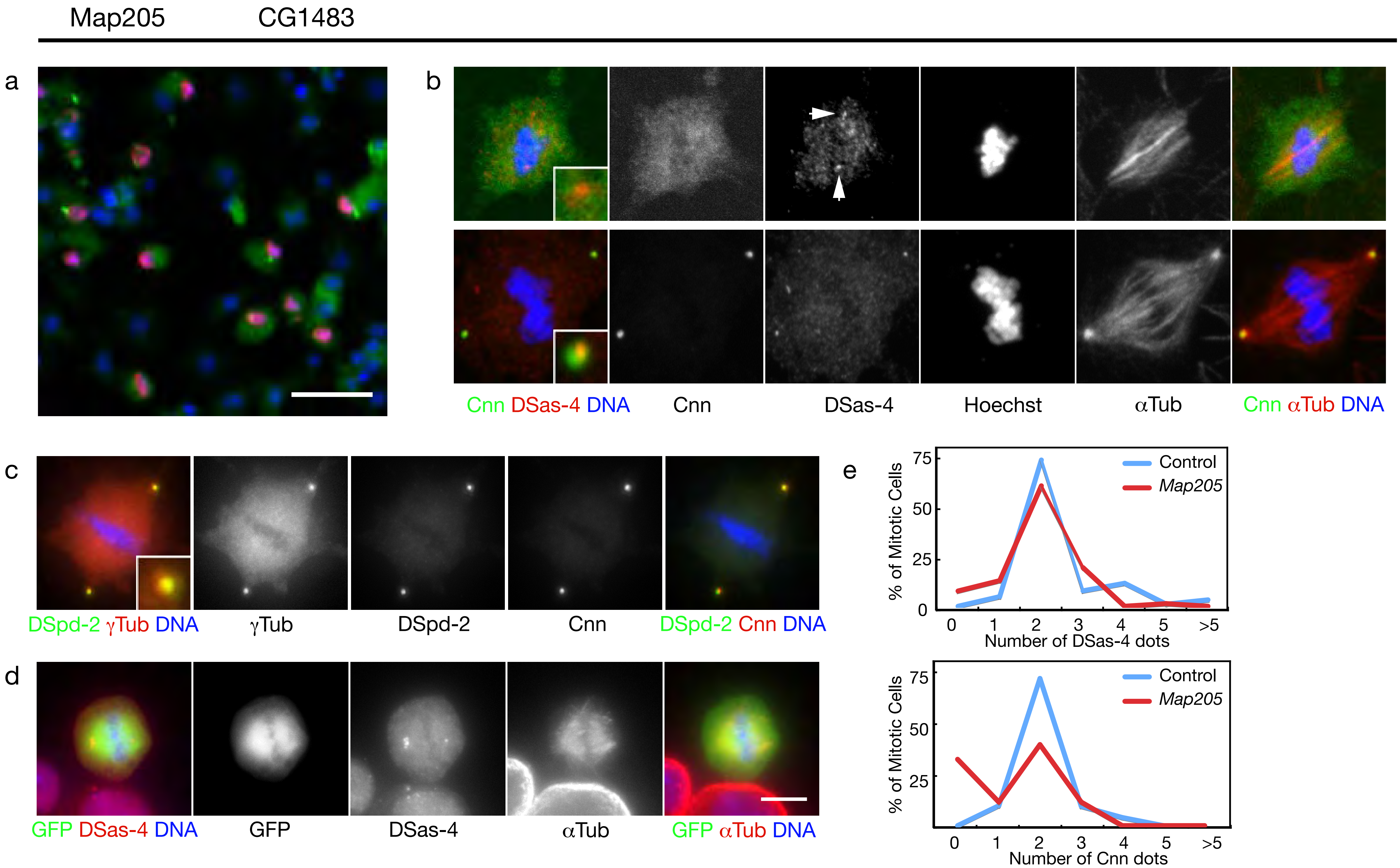

Map205
CG1483
a
b
Cnn DSas-4 DNA
Cnn
DSas-4
Hoechst
αTub
Cnn αTub DNA
c
DSpd-2 γTub DNA
γTub
DSpd-2
Cnn
DSpd-2 Cnn DNA
d
GFP DSas-4 DNA
GFP
DSas-4
αTub
GFP αTub DNA
e
% of Mitotic Cells
Control
Map205
Number of DSas-4 dots
Number of Cnn dots
0
1
2
3
4
5
>5
25
50
75

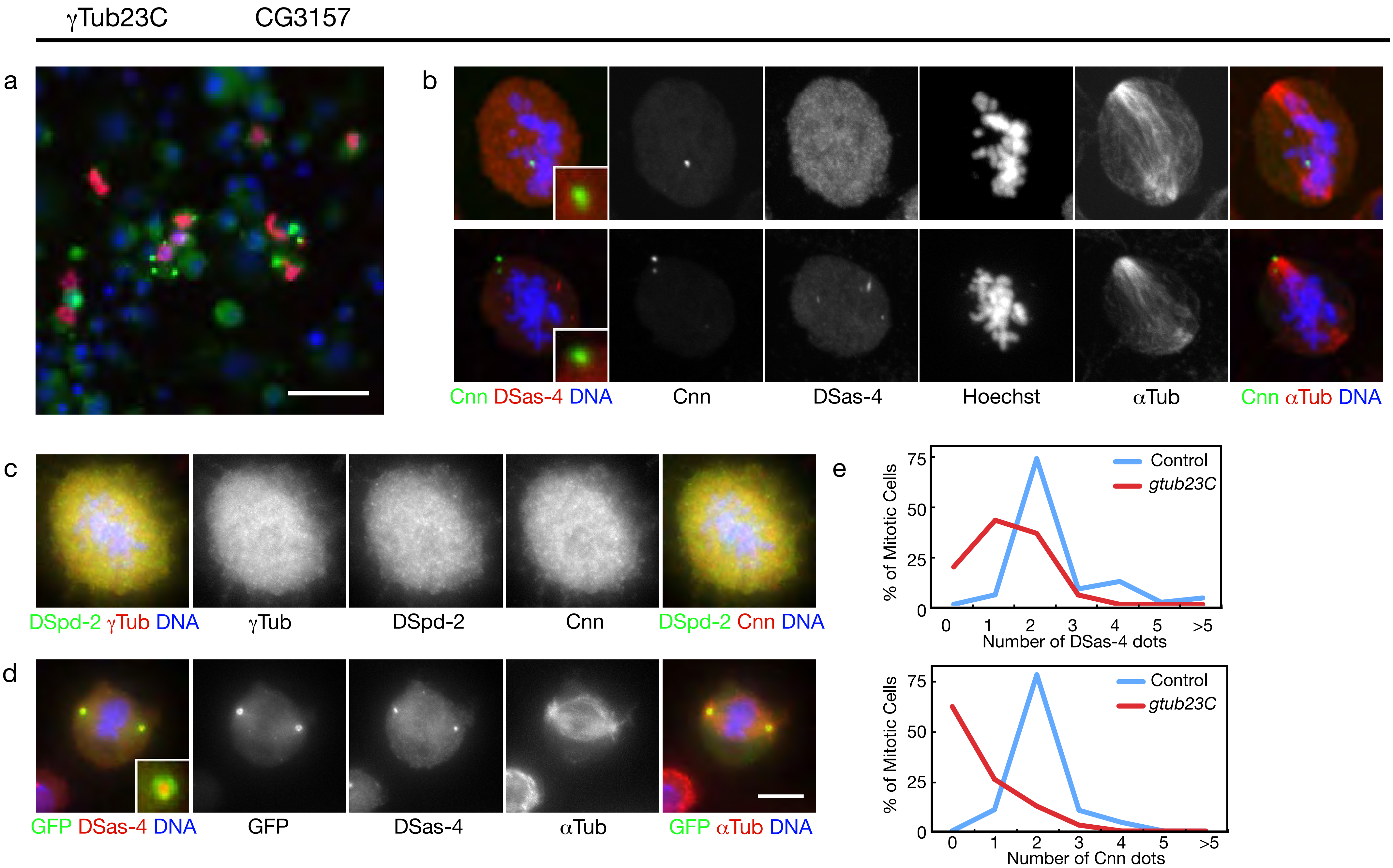
γTub23C
CG3157
a
b
Cnn DSas-4 DNA
Cnn
DSas-4
Hoechst
αTub
Cnn αTub DNA
c
DSpd-2 γTub DNA
γTub
DSpd-2
Cnn
DSpd-2 Cnn DNA
d
GFP DSas-4 DNA
GFP
DSas-4
αTub
GFP αTub DNA
e
% of Mitotic Cells
Control
gtub23C
Number of DSas-4 dots
% of Mitotic Cells
Control
gtub23C
Number of Cnn dots

D-PLP CG13459

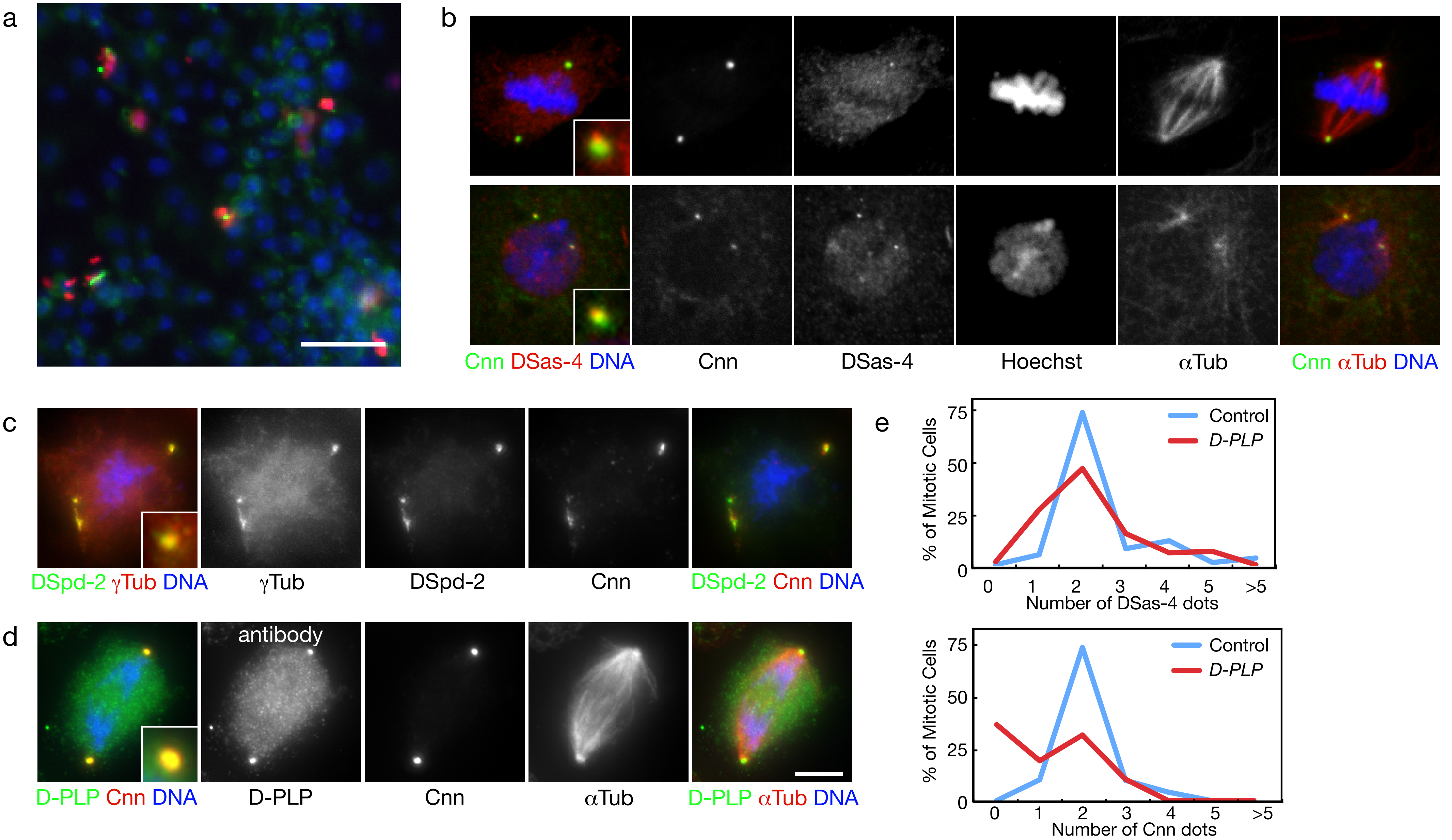

Rcd6 CG11175

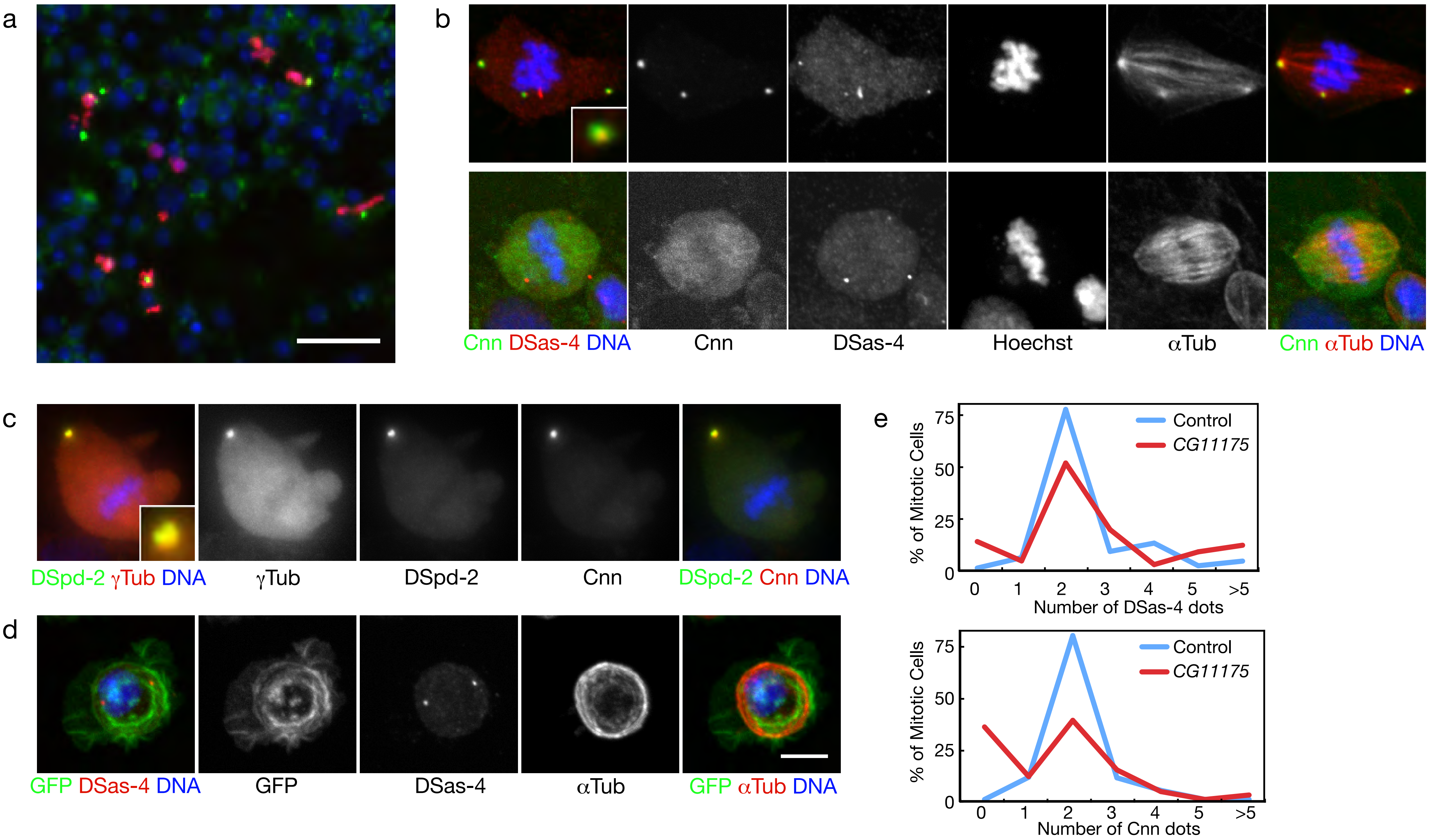

tws CG6235

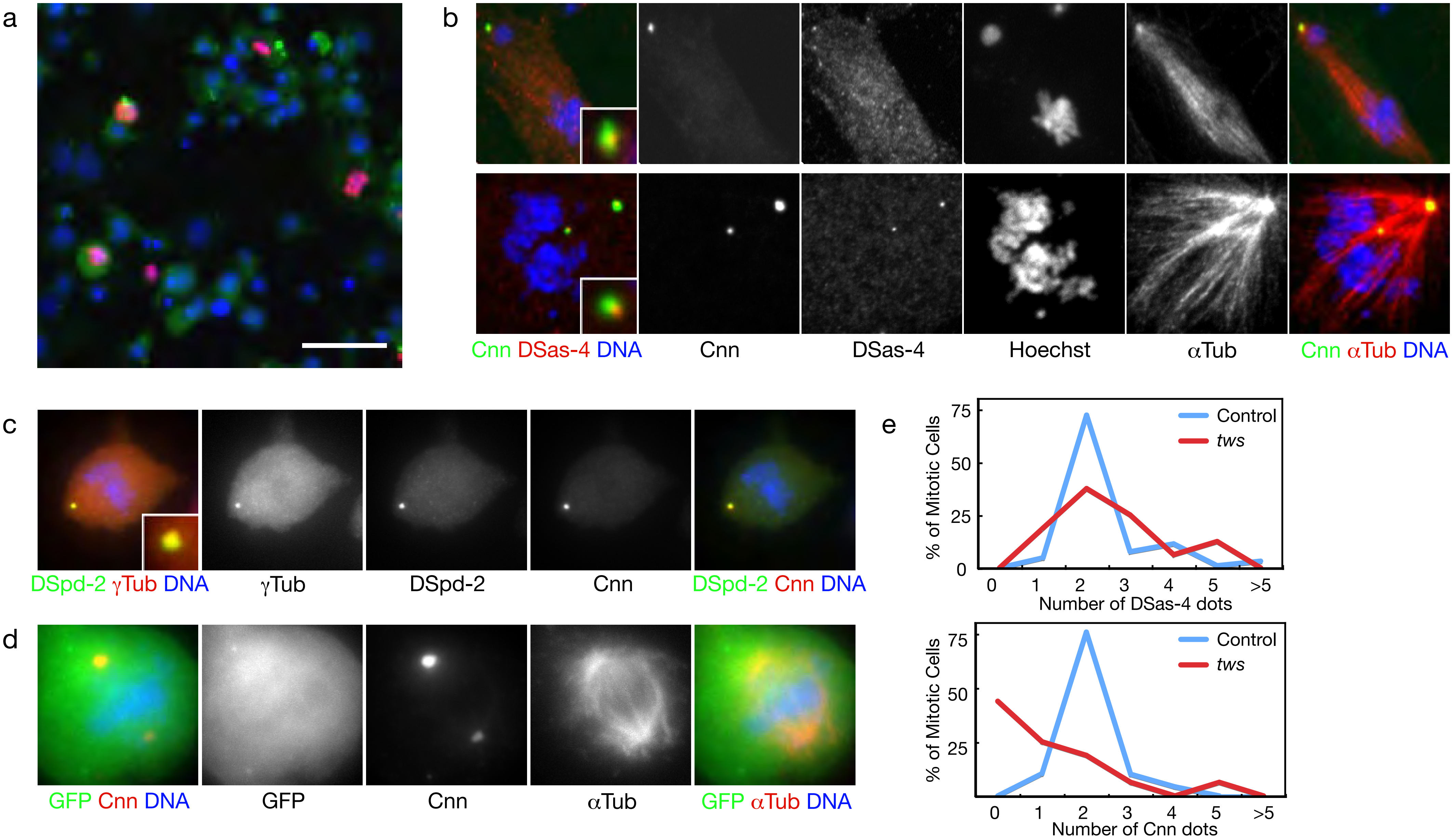

a
b
Cnn DSas-4 DNA
Cnn
DSas-4
Hoechst
αTub
Cnn αTub DNA
c
DSpd-2 γTub DNA
γTub
DSpd-2
Cnn
DSpd-2 Cnn DNA
d
GFP Cnn DNA
GFP
Cnn
αTub
GFP αTub DNA
e
% of Mitotic Cells
75
50
25
0
Control
tws
0 1 2 3 4 5 >5
Number of DSas-4 dots
% of Mitotic Cells
75
50
25
Control
tws
0 1 2 3 4 5 >5
Number of Cnn dots

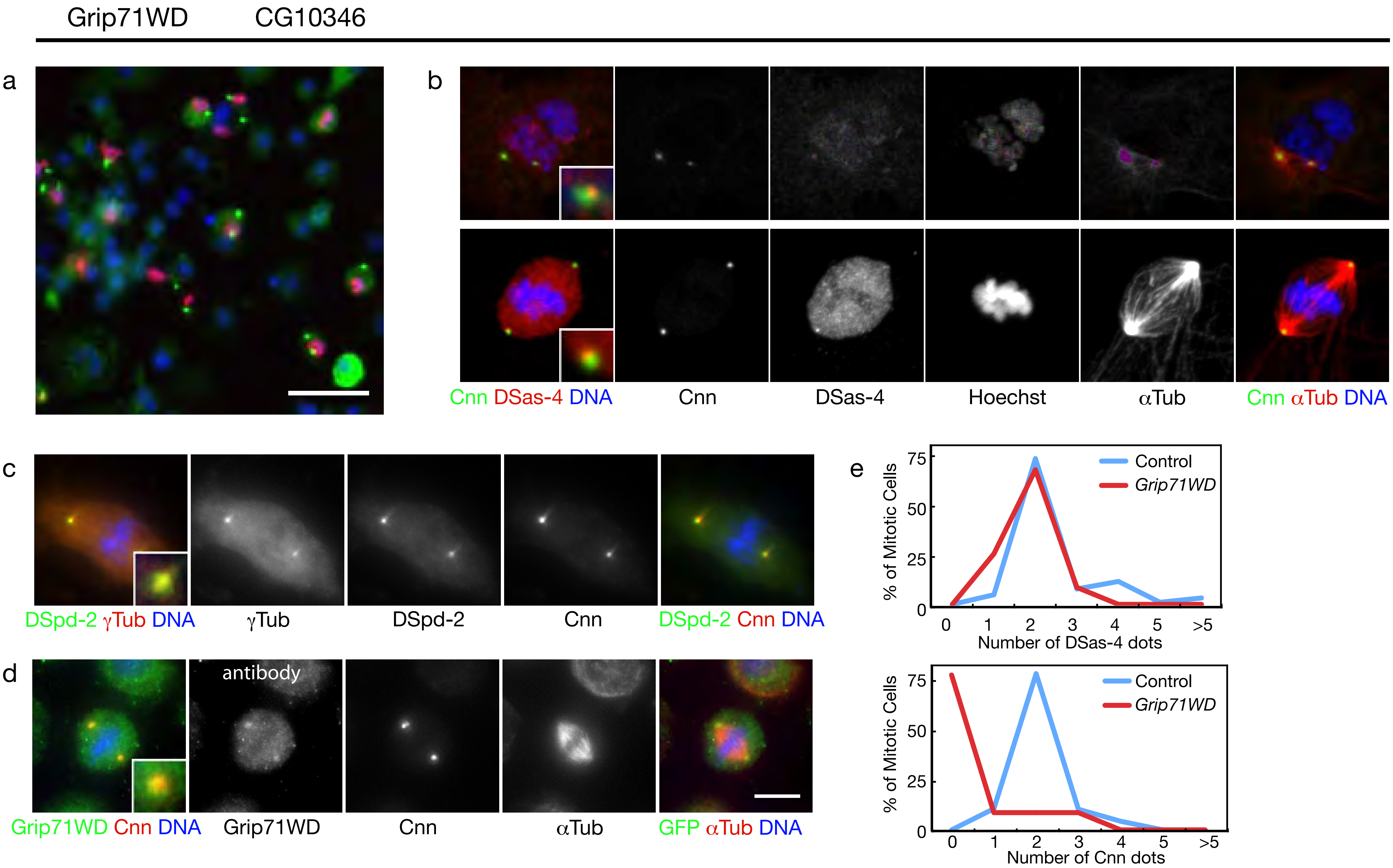
Grip71WD CG10346
a
b
Cnn DSas-4 DNA
Cnn
DSas-4
Hoechst
αTub
Cnn αTub DNA
c
DSpd-2 γTub DNA
γTub
DSpd-2
Cnn
DSpd-2 Cnn DNA
d
antibody
Grip71WD Cnn DNA
Grip71WD
Cnn
αTub
GFP αTub DNA
e
% of Mitotic Cells
Control
Grip71WD
75
50
25
0
0 1 2 3 4 5 >5
Number of DSas-4 dots
% of Mitotic Cells
Control
Grip71WD
75
50
25
0
0 1 2 3 4 5 >5
Number of Cnn dots

PP2A-29B CG17291

a

Not in Original Screen

b

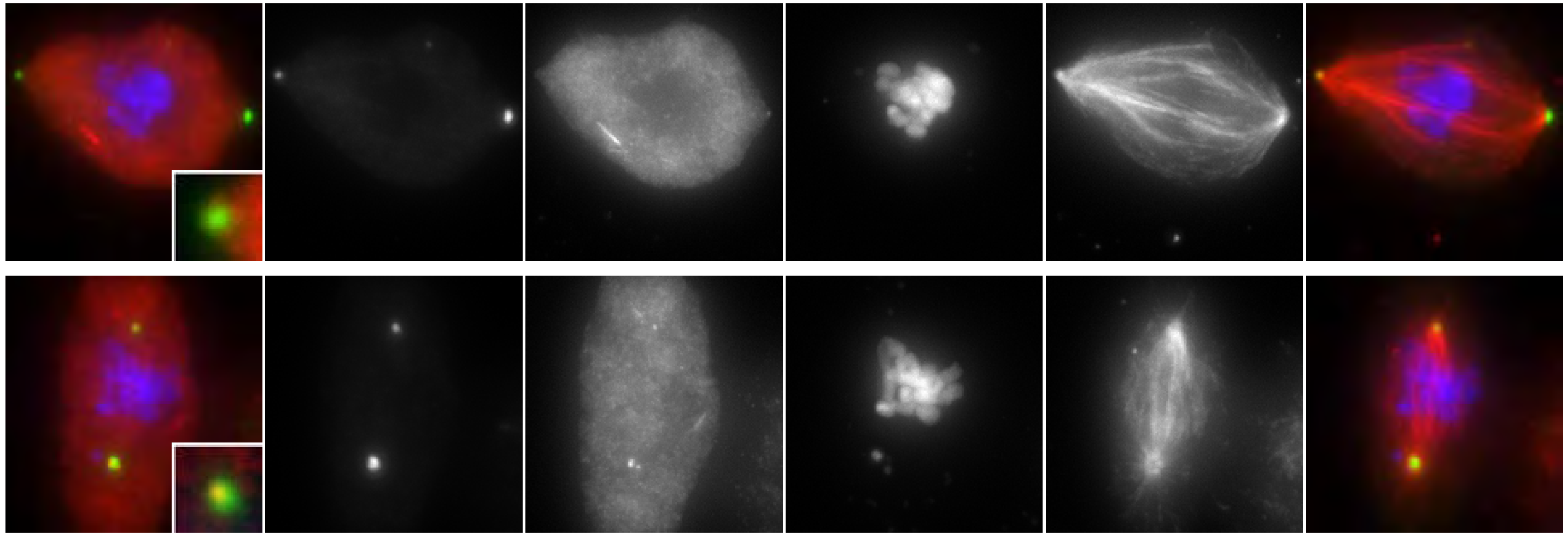

c

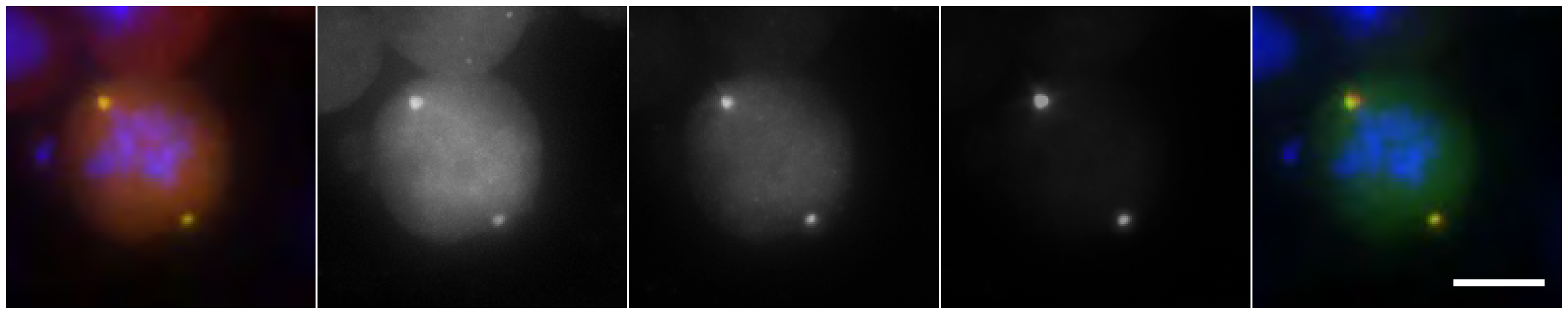

d

Localization not Tested

GFP DSas-4 DNA GFP DSas-4 αTub GFP αTub DNA

e

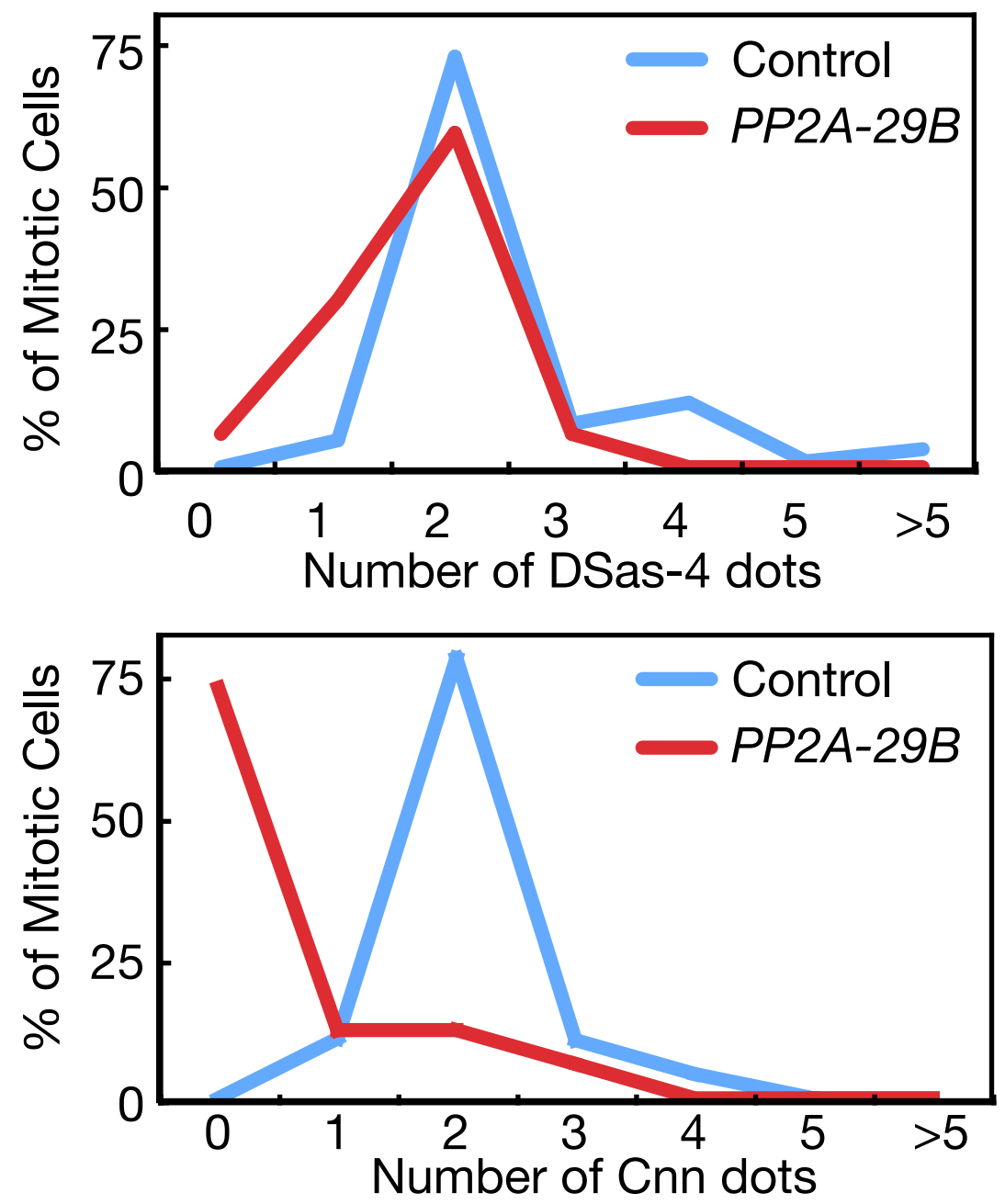

mts CG7109

a

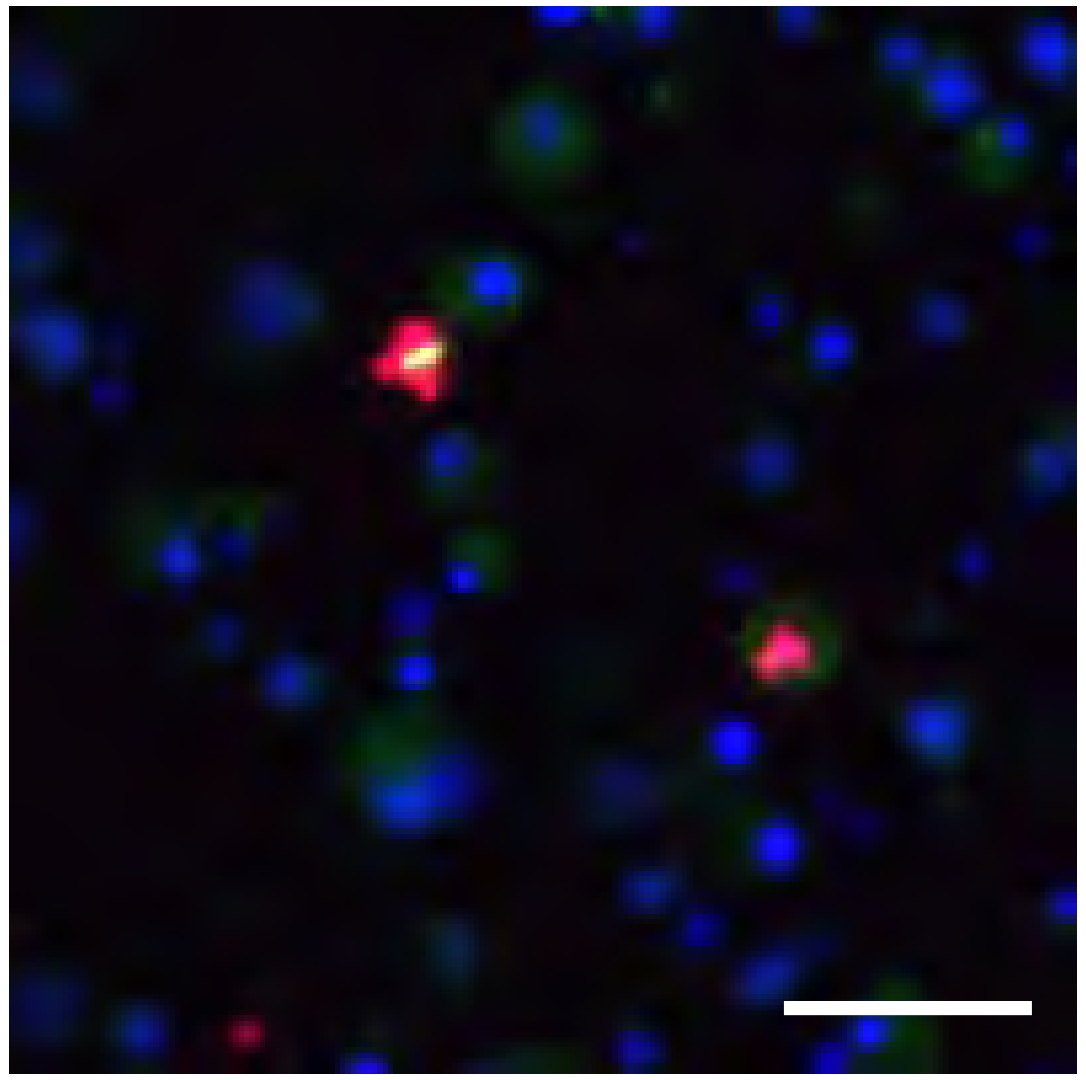

b

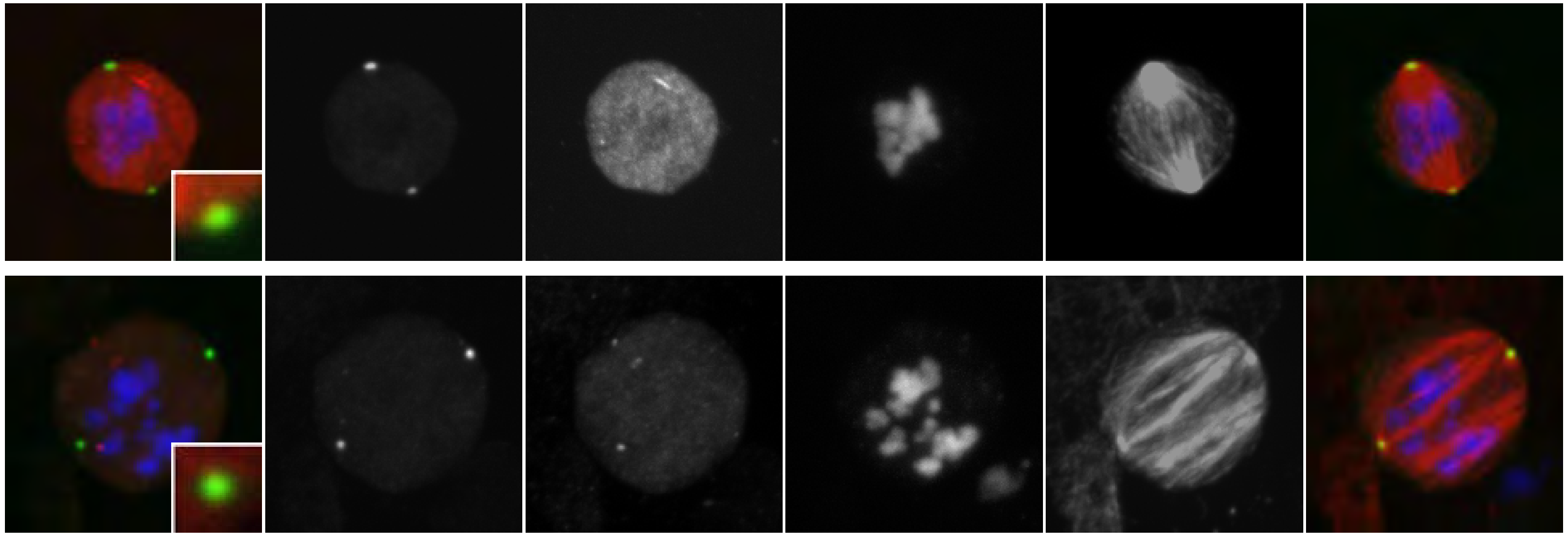

c

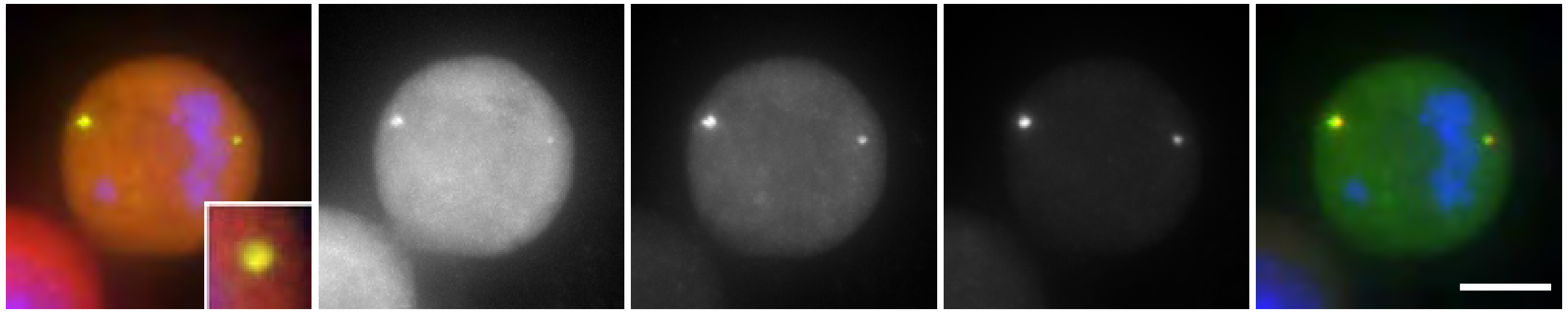

d

Localization not Tested

e

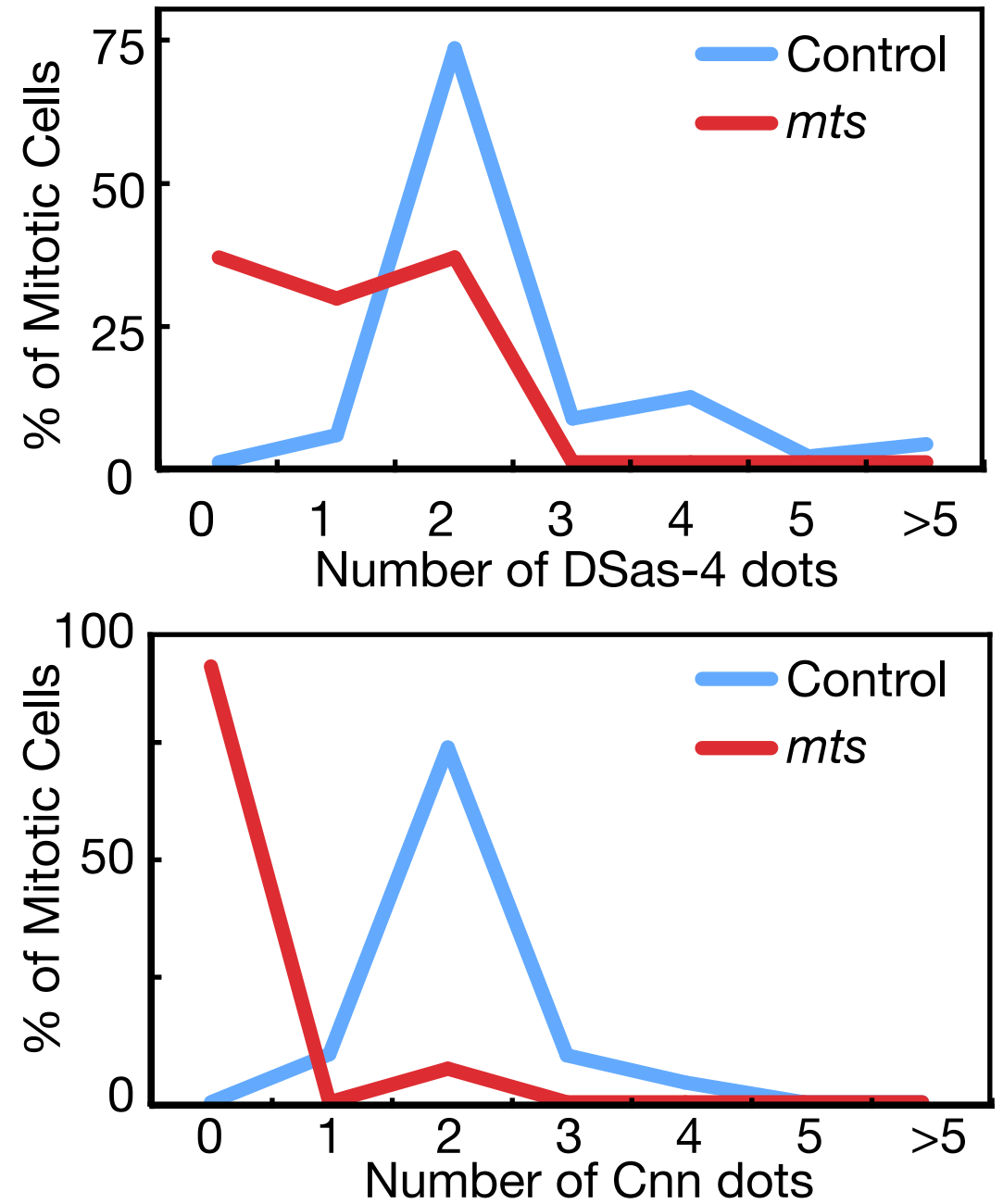

Class IV :
Centrosome Separation Defects

UbcD6 CG2013

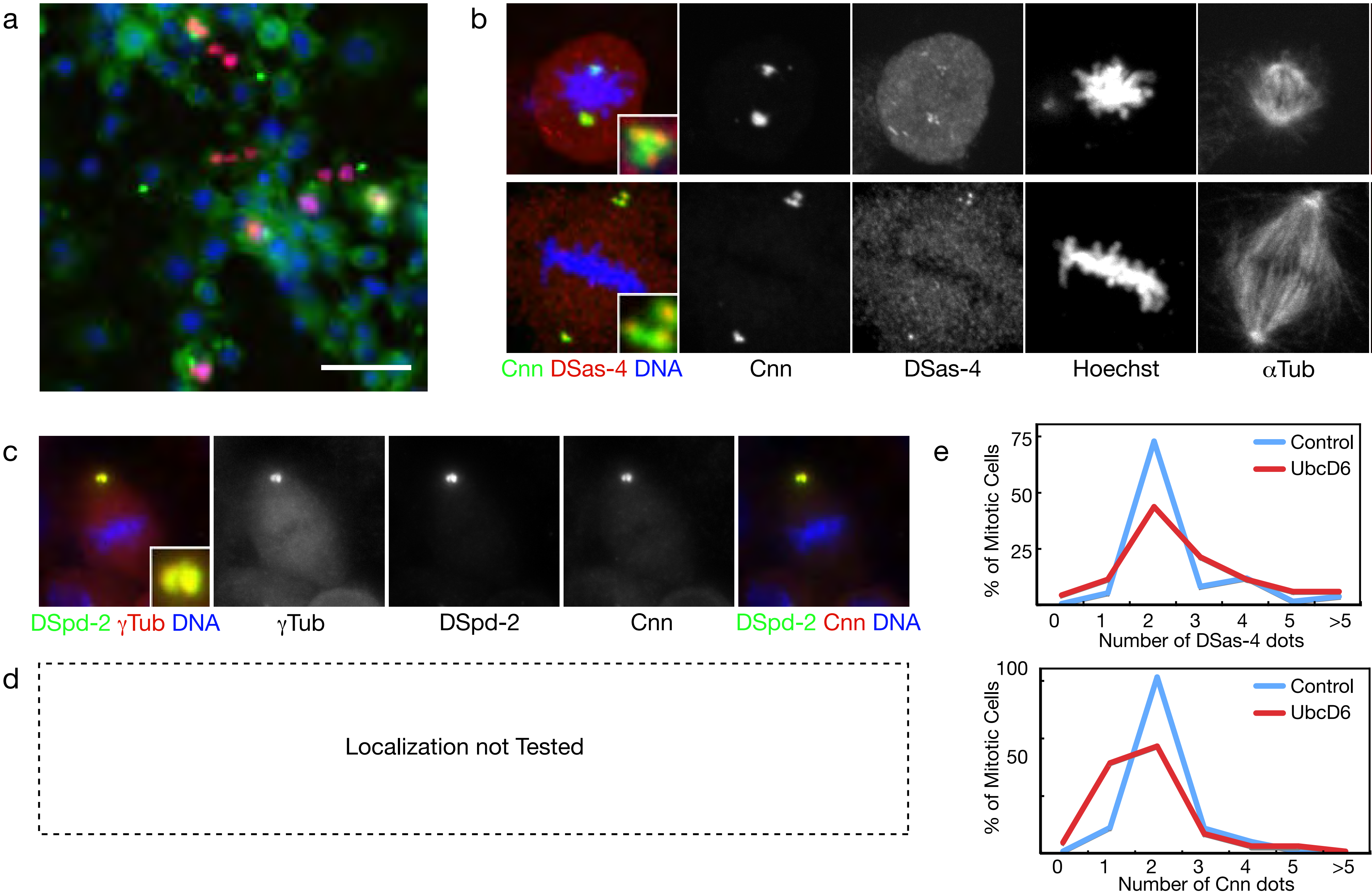

ial CG6620

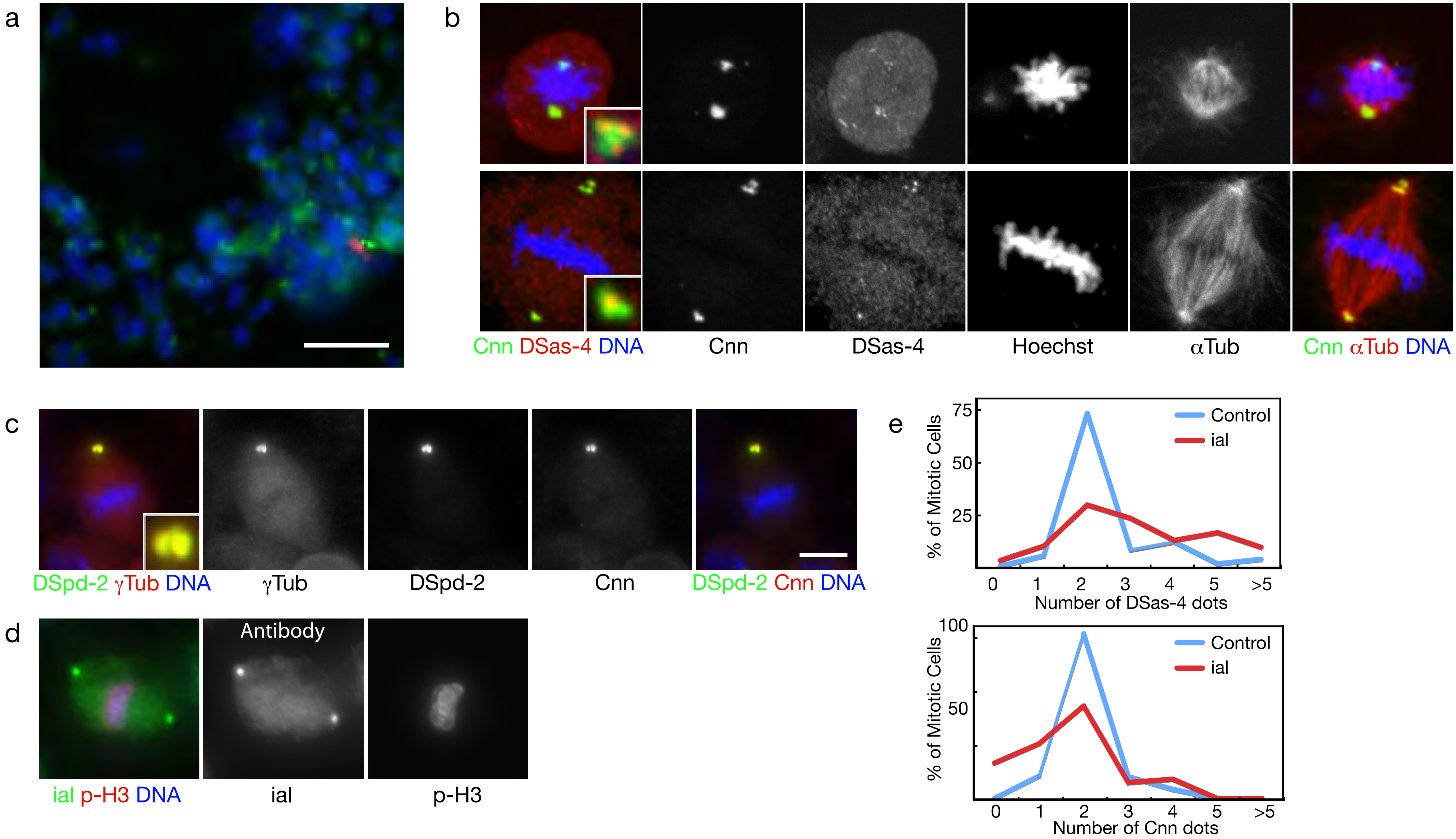

a
b
Cnn DSas-4 DNA
Cnn
DSas-4
Hoechst
αTub
Cnn αTub DNA
c
DSpd-2 γTub DNA
γTub
DSpd-2
Cnn
DSpd-2 Cnn DNA
d
Antibody
ial p-H3 DNA
ial
p-H3
e
Control
ial
% of Mitotic Cells
75
50
25
0
1
2
3
4
5
>5
Number of DSas-4 dots
100
50
Number of Cnn dots

Rcd7 CG14098

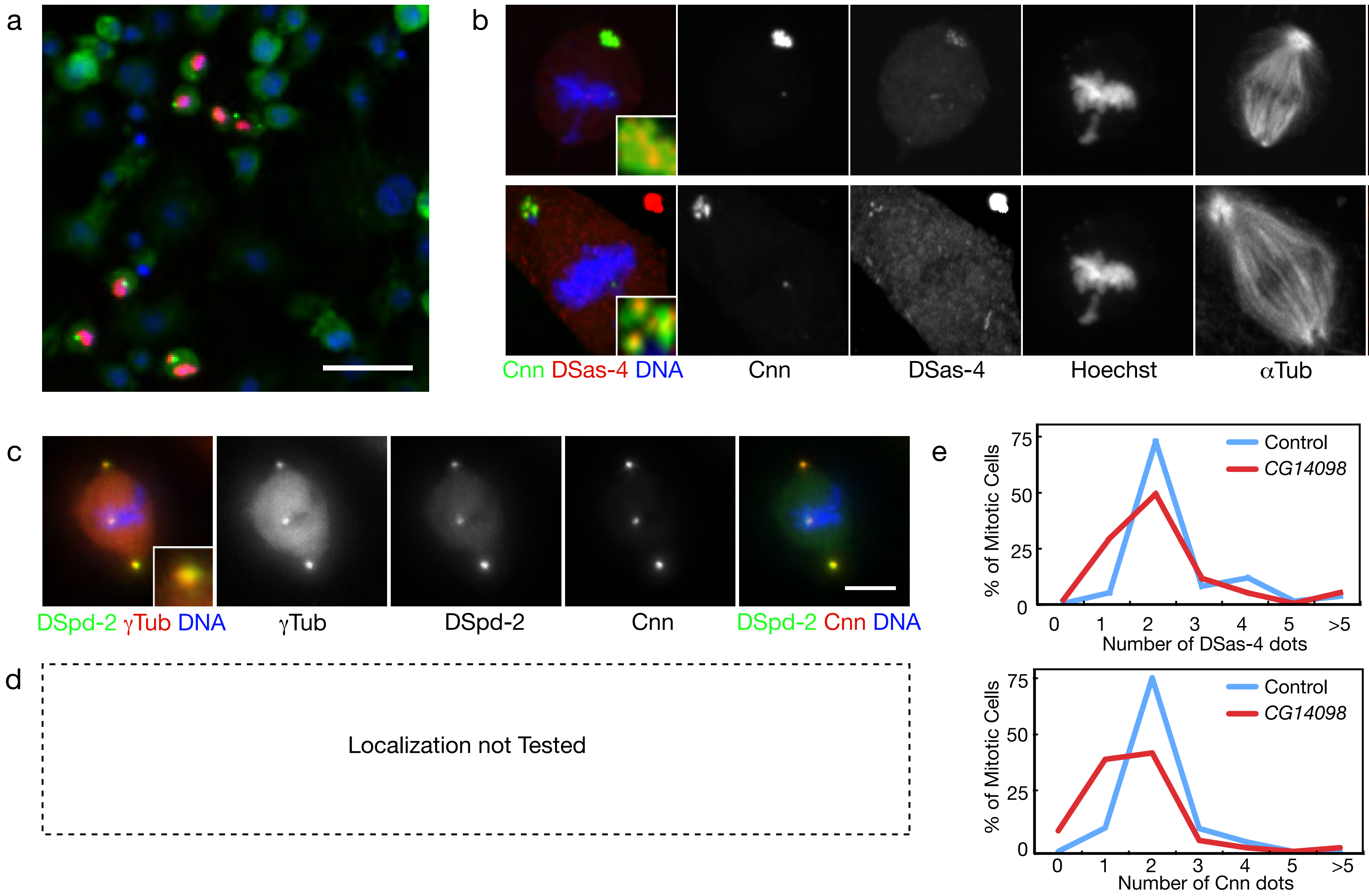

Supplement: Supplementary file 1 [file pbio.0060224.sd001.pdf]
