# Supplementary material for: A Genome-Wide RNAi Screen to Dissect Centriole Duplication and Centrosome Maturation in Drosophila
Source: PLoS Biol. 2008 Sep 16;6(9):e224. doi: 10.1371/journal.pbio.0060224 (PMC2535660; doi:10.1371/journal.pbio.0060224)
Supplement: Supplementary file 2 [file pbio.0060224.sg001.pdf]

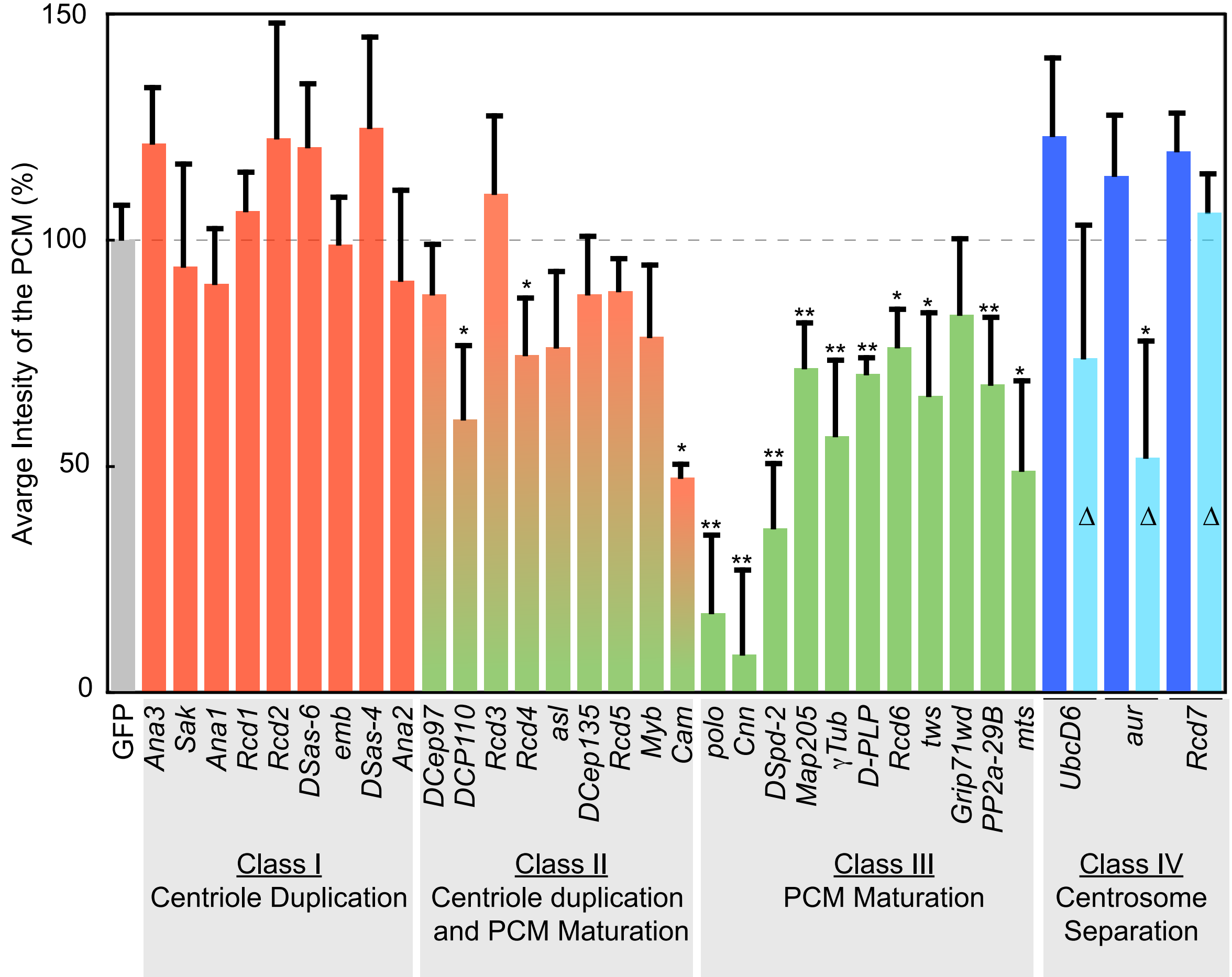
Avarge Intesity of the PCM (%)
150
100
50
0
GFP
Ana3
Sak
Ana1
Rcd1
Rcd2
DSas-6
emb
DSas-4
Ana2
DCep97
DCP110
Rcd3
Rcd4
asl
DCep135
Rcd5
Myb
Cam
polo
Cnn
DSpd-2
Map205
γTub
D-PLP
Rcd6
tws
Grip71wd
PP2a-29B
mts
UbcD6
aur
Rcd7
Δ
Δ
Δ
Class I
Centriole Duplication
Class II
Centriole duplication
and PCM Maturation
Class III
PCM Maturation
Class IV
Centrosome
Separation
