# Supplementary material for: A Genome-Wide RNAi Screen to Dissect Centriole Duplication and Centrosome Maturation in Drosophila
Source: PLoS Biol. 2008 Sep 16;6(9):e224. doi: 10.1371/journal.pbio.0060224 (PMC2535660; doi:10.1371/journal.pbio.0060224)
Supplement: Supplementary file 3 [file pbio.0060224.sg002.pdf]

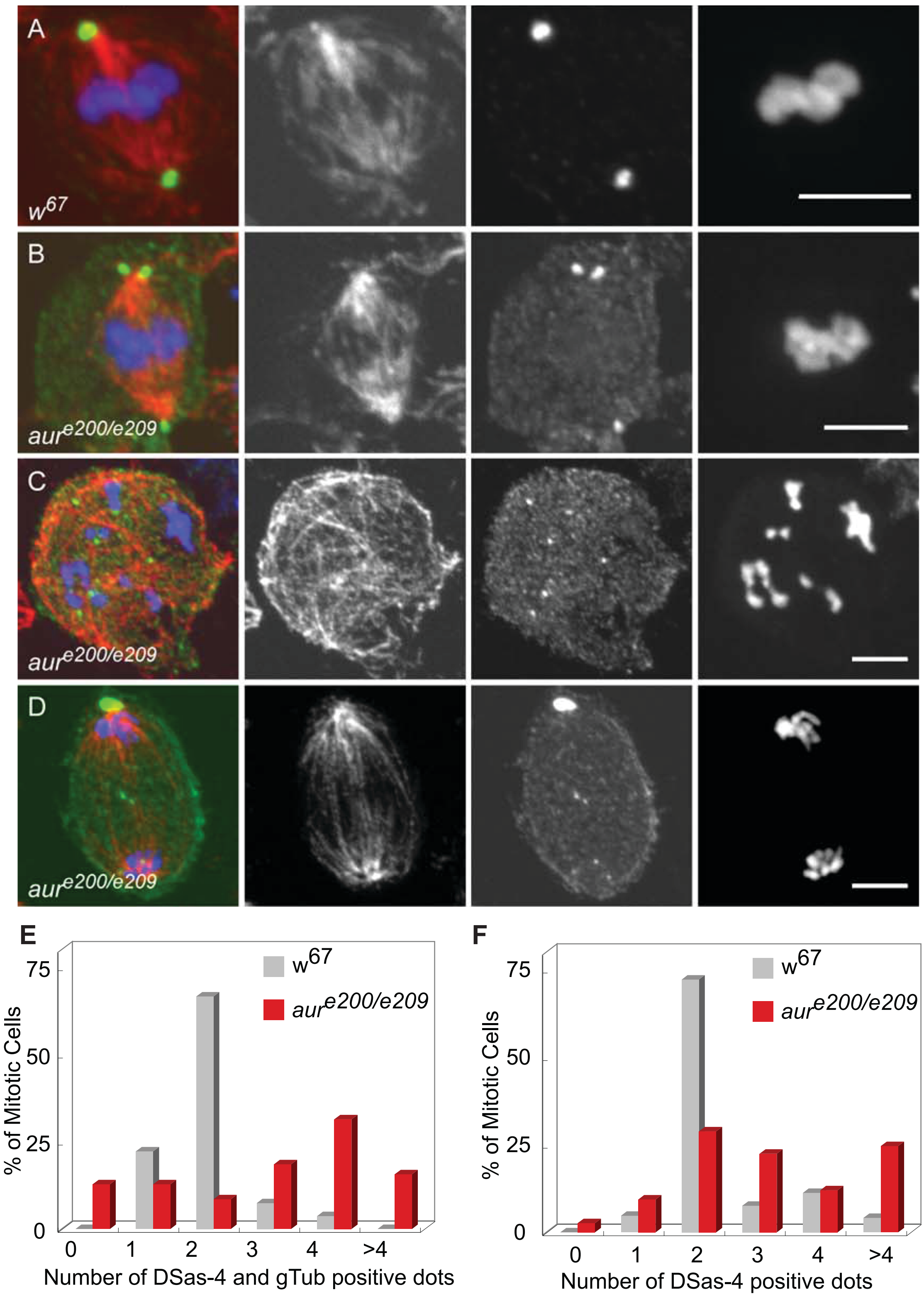

A
w67
B
aure200/e209
C
aure200/e209
D
aure200/e209
E
w67
aure200/e209
% of Mitotic Cells
75
50
25
0
0
1
2
3
4
>4
Number of DSas-4 and gTub positive dots
F
w67
aure200/e209
% of Mitotic Cells
75
50
25
0
0
1
2
3
4
>4
Number of DSas-4 positive dots
