# Supplementary material for: A Genome-Wide RNAi Screen to Dissect Centriole Duplication and Centrosome Maturation in Drosophila
Source: PLoS Biol. 2008 Sep 16;6(9):e224. doi: 10.1371/journal.pbio.0060224 (PMC2535660; doi:10.1371/journal.pbio.0060224)
Supplement: Supplementary file 4 [file pbio.0060224.sg003.pdf]

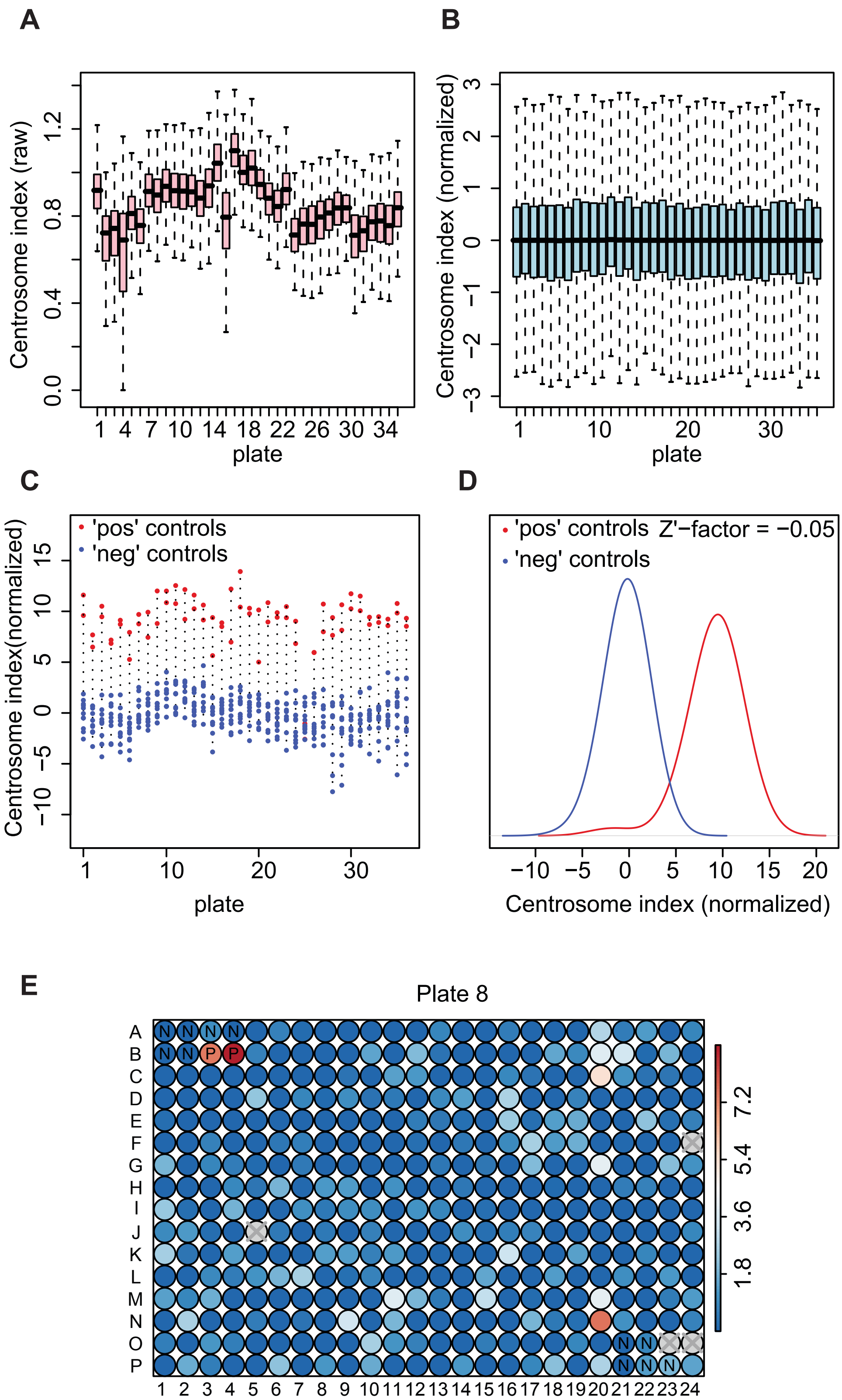
A
Centrosome index (raw)
plate
B
Centrosome index (normalized)
plate
C
'pos' controls
'neg' controls
Centrosome index(normalized)
plate
D
'pos' controls Z'−factor = −0.05
'neg' controls
Centrosome index (normalized)
E
Plate 8
A B C D E F G H I J K L M N O P
1 2 3 4 5 6 7 8 9 10 11 12 13 14 15 16 17 18 19 20 21 22 23 24
N
P
1.8
3.6
5.4
7.2
