# Supplementary material for: A Genome-Wide RNAi Screen to Dissect Centriole Duplication and Centrosome Maturation in Drosophila
Source: PLoS Biol. 2008 Sep 16;6(9):e224. doi: 10.1371/journal.pbio.0060224 (PMC2535660; doi:10.1371/journal.pbio.0060224)
Supplement: Supplementary file 5 [file pbio.0060224.sg004.pdf]

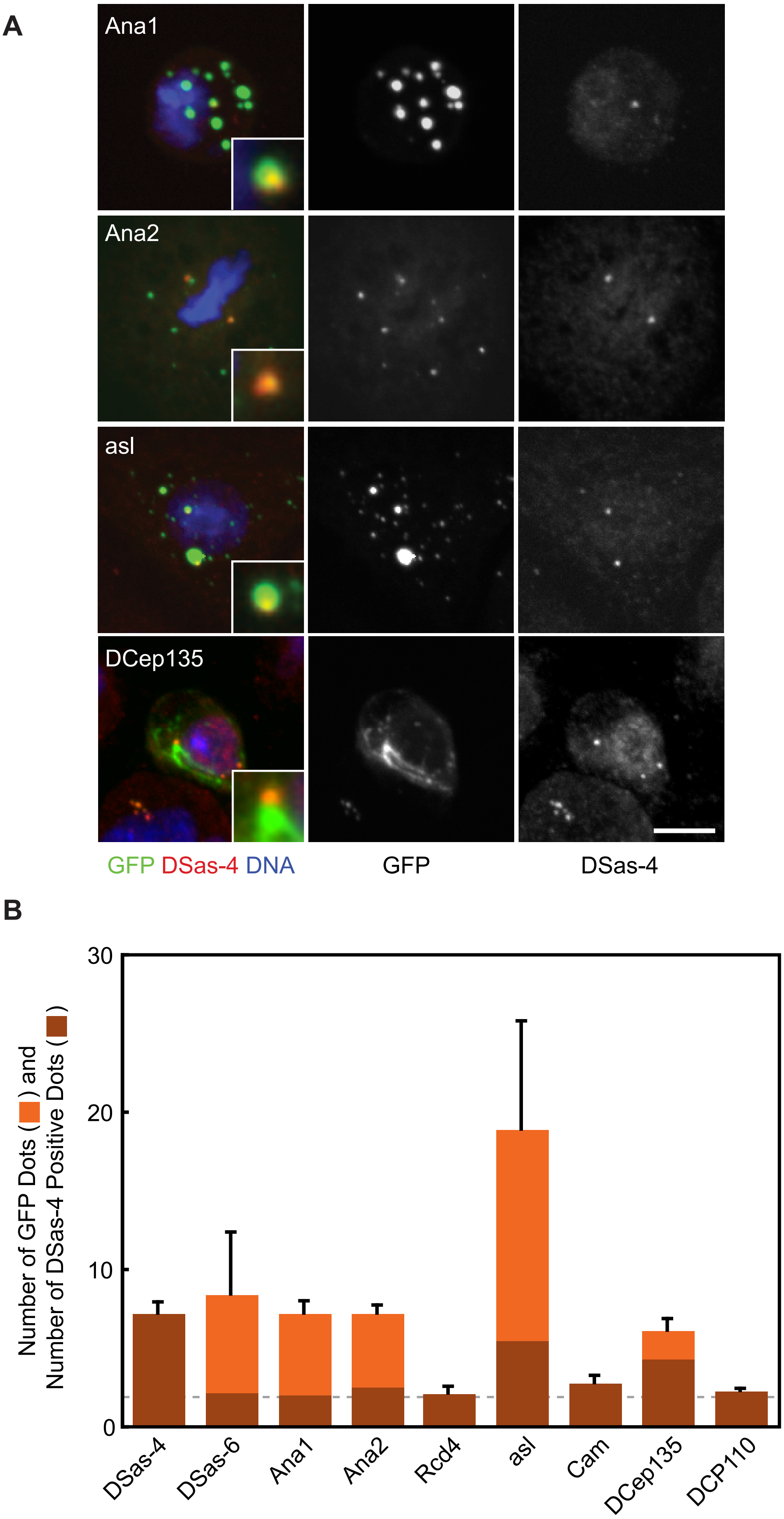
A
Ana1
Ana2
asl
DCep135
GFP DSas-4 DNA
GFP
DSas-4
B
Number of GFP Dots ( ) and
Number of DSas-4 Positive Dots ( )
0
10
20
30
DSas-4
DSas-6
Ana1
Ana2
Rcd4
asl
Cam
DCep135
DCP110
