# Supplementary material for: A Genome-Wide RNAi Screen to Dissect Centriole Duplication and Centrosome Maturation in Drosophila
Source: PLoS Biol. 2008 Sep 16;6(9):e224. doi: 10.1371/journal.pbio.0060224 (PMC2535660; doi:10.1371/journal.pbio.0060224)
Supplement: Supplementary file 6 [file pbio.0060224.st001.pdf]

Supplementary Table 1. Genes Scored in Primary Screen

	CG	Name	Function	Manual Scoring	Picture Scoring	Cell proflier	Selected (Yes/No)	384 count +C	384 size +C	96 count +C	96 size +C	384 count	384 size	96 count	96 size	spd2 count	spd2 size	γtub count	γtub size	spindle	Selected for Secondary screening
1	CG4832	Cnn	Centrosome	3	3	9.58	Yes	3	3	3	3	3	3	3	3	3	3	3	3	0	x
2	CG12306	Polo	Kinase/Phosphatase	3	3	7.73	Yes	3	3	3	3	3	3	3	3	3	3	3	3	2	x
3	CG18217		Unknown	3	3	6.59	No (Off targed effect Spd2)														
4	CG1483	Map205	Cytoskeleton	3	2	6.19	Yes	2	3	2	2	2	2	2	2	1	1	2	2	0	x
5	CG15929	lin-52	Transcription/RNA Polymerase	3	2	5.72	No (Transcription factor)														
6	CG2919	Asterless	Centrosome	3	2	4.33	Yes	2	3	2	2	3	1	2	1	2	2	2	2	2	x
7	CG13467		Unknown	3	1	8.07	Yes	1	0	0	0	0	0	2	2	2	1	0	0	2	x
8	CG17286	D-Spd2	Centrosome	3	1	6.28	Yes	3	2	1	3	2	2	2	2	3	3	2	2	0	x
9	CG8472	Cam	Other	3	1	5.3	Yes	2	2	2	2	2	3	2	2	2	2	2	2	3	x
10	CG5887	desat1	Lipid metabolism	3	0	3.51	No														
11	CG9045	Myb	Transcription/RNA Polymerase	2	3	9.05	Yes	2	3	2	1	2	3	3	2	2	2	2	2	1	x
12	CG1668	Pbprp2	Other	2	3	7.25	Yes	1	0	1	0	0	0	0	0	0	0	0	0	0	
13	CG31484	CG31484	Unknown	2	3	3.05	Yes	0	0	2	1	0	0	0	0	0	0	0	0	0	
14	CG4786		Unknown	2	2	5.64	Yes	2	3	2	2	2	3	2	2	2	3	2	3	2	x
15	CG6631	Ana1	Centrosome	2	2	5.26	Yes	2	3	2	2	3	3	2	2	3	3	3	3	1	x
16	CG13162	Ana3	Unknown	2	2	4.62	Yes	2	3	2	2	2	3	3	3	3	3	2	2	1	x
17	CG10061	D-Sas4 / l(3)s2214	Centrosome	2	2	4.01	Yes	2	2	2	0	3	0	2	0	2	2	2	2	0	x
18	CG11175		Unknown	2	2	3.83	Yes	2	2	1	0	1	0	2	2	2	1	1	1	2	x
19	CG14617		Centrosome	2	2	3.55	Yes	2	3	2	3	2	2	2	3	2	2	2	2	0	x
20	CG40397		Unknown	2	2	3.3	Yes	0	0	0	0	0	0	0	0	0	0	0	0	0	
21	CG12678		Unknown	2	2	2.89	Yes	1	0	0	0	0	0	0	2	0	1	0	0	0	
22	CG8308	[alpha]Tub67C	Cytoskeleton	2	2	2.87	No (Cells very Sick)														
23	CG10641		Unknown	2	2	2.66	Yes	1	2	0	0	0	0	1	1	0	0	0	0	0	
24	CG15524	Sas-6	Centrosome	2	2	2.33	Yes	2	2	2	1	2	2	2	2	2	0	2	0	0	x

25	CG3131		Other	2	2	0.89	Yes	0	0	0	0	0	0	0	0	2	1	0	0	3	
26	CG18377	Cyp49a1	Other	2	2	-1.4	Yes	2	1	0	0	0	0	0	0	0	0	0	0	0	
27	CG5461	bun	Transcription/RNA Polymerase	2	1	4.01	No (Transcription factor)														
28	CG1906		Kinase/Phosphatase	2	1	3.71	Yes	1	1	0	0	1	1	1	1	0	0	0	0	0	x
29	CG6235	Tws	Kinase/Phosphatase	2	1	3.59	Yes														
30	CG9476	[alpha]Tub85E	Cytoskeleton	2	1	3.3	No (Cells very Sick)														
31	CG8805	wunen-2	Lipid metabolism	2	0	5.92	Falls Positive														
32	CG32782	CG32782	Kinase/Phosphatase	2	0	5.28	Yes	2	2	2	2	0	2	0	0						x
33	CG11451	CG11451		2	0	4.81	No														
34	CG10353	CG10353	Unknown	2	0	4.2	No														
35	CG18572	r	Other	2	0	4.12	Yes	0	0	0	0	0	0	0	0	0	0	0	0	0	
36	CG15770		Unknown	2	0	3.91	Yes	0	0	1	1	1	2	2	1	0	0	0	0	0	x
37	CG9697	PGRP-SB2	Other	2	0	3.88	Yes	1	1	0	0	0	0	1	2	0	0	0	0	0	
38	CG9388	AP-47	Other	2	0	2.86	No														
39	CG14641	CG14641	Transcription/RNA Polymerase	2	0	2.85	No (Transcription factor)														
40	CG1135		Transcription/RNA Polymerase	2	0	2.63	Yes	2	3	1	1	2	2	0	0	2	3	2	3	2	x
41	CG8153	mutagen-sensitive 210	Other	2	0	2.57	Falls Positive														
42	CG1622	CG1622	Transcription/RNA Polymerase	2	0	2.54	No (Transcription factor)														
43	CG9696	domino	Transcription/RNA Polymerase	2	0	2.43	No (Transcription factor)														
44	CG1666	Helicase	Transcription/RNA Polymerase	2	0	2.32	No (Transcription factor)														
45	CG7639	CG7639	Unknown	2	0	2.32	Falls Positive														
46	CG8262	Ana2	Centrosome	2	0	2.27	Yes	2	2	2	2	2	2	2	2						x

47	CG4140		Unknown	2	0	2.24	Yes	0	0	0	0	0	0	0	0	0	0	0	0	0	
48	CG32176		Unknown	2	0	2	Yes	0	0	0	0	0	0	0	0	0	0	0	0	0	
49	CG10346	Grip71wd	Centrosome	2	0	1	Yes	2	1	1	0					2	2			3	x
50	CG30164		Lipid metabolism	2	0	0.08	Yes	0	0	0	0	0	0	0	0	0	0	0	0	0	
51	CG7186	Sak	Kinase/Phosphatase	2	0	-0.2	Yes	2	2	2	2	2	2	2	0	2	1			3	x
52	CG2791		Other	1	2	7.12	Yes	1	0	0	0	0	0	0	0	0	0	0	0	0	
53	CG8233		Other	1	2	6.25	Yes	2	1	1	1	2	2	2	2	0	0	3	2	0	x
54	CG11327		Unknown	1	2	5.97	Yes	0	0	0	0	0	0	0	0	0	0	0	0	0	
55	CG13387	emb	Other	1	2	2.8	Yes	2	2	1	2	2	2	3	2	2	2	2	2	1	x
56	CG8231	l(1)G0022	Protein folding	1	2	1.02	Yes	0	0	2	1	0	0	1	2	0	0	2	2	0	x
57	CG11052		Protein folding	1	1	1.99	Yes	0	0	0	0	1	2	0	0	0	0	0	1	0	
58	CG4913	ear	Transcription/RNA Polymerase	1	1	1.82	No (Transcription factor)														
59	CG8687	Cyp6a14	Other	1	1	-0.3	Falls Positive	0	0	1	0	0	0	2	0	0	0	0	0	0	
60	CG9078	ifc	Lipid metabolism	1	0	5.47	Yes	0	0	0	0	0	0	0	0	0	0	0	0	0	
61	CG6474	enhancer of yellow 1	Transcription/RNA Polymerase	1	0	4.82	No (Transcription factor)														
62	CG17295		Unknown	1	0	4.47	Yes	2	2	2	2	2	2	2	2	2	1	2	3	2	x
63	CG31175	Dys or CG7344	Cytoskeleton	1	0	2.91	Yes	2	0	0	0	0	0	0	0	0	0	0	0	0	
64	CG14098	CG14098	Unknown	1	0	2.7	Yes	1	1	1	2	0	0	1	1						x
65	CG32177		Protein degradation	1	0	2.62	Yes	0	0	0	0	1	1	1	0	0	0	0	0	0	
66	CG2046		Unknown	1	0	2.59	Yes	2	0	0	0	2	1	0	0	0	0	0	0	0	
67	CG6479	CG6479	Other	1	0	2.49	Falls Positive														
68	CG13830	CG13830	Protein degradation	1	0	2.22	Falls Positive														
69	CG8640		Unknown	1	0	1.66	Yes	2	0	1	0	0	0	1	0	0	0	0	0	1	
70	CG10042	MBD-R2	Transcription/RNA Polymerase	0	2	5.86	No (Transcription factor)														
71	CG11312	insc	Other	0	2	5.48	Yes	1	0	1	1	0	1	0	0	0	0	0	0	0	
72	CG9147		Other	0	2	4.75	Yes	0	0	0	0	2	1	0	0	0	0	0	0	0	
73	CG17081		Centrosome	0	2	4.52	Yes	2	3	2	3	2	2	1	2	1	1	1	2	0	x

74	CG1746	CG1746	Lipid metabolism	0	2	4.25	Falls Positive														
75	CG9797	CG9797	Transcription/RNA Polymerase	0	2	4.09	No (Transcription factor)														
76	CG7833	Orc5	Transcription/RNA Polymerase	0	2	3.69	No (Transcription factor)														
77	CG10863		Other	0	2	3.61	Yes	0	0	0	0	0	0	1	1	0	0	0	0	0	
78	CG8351		Protein folding	0	2	3.39	Yes	2	2	0	0	0	0	0	0	0	0	0	0	0	
79	CG18009	TATA box binding protein-related factor 2	Transcription/RNA Polymerase	0	2	3.37	No (Transcription factor)														
80	CG2714	crm	Transcription/RNA Polymerase	0	2	3.22	No														
81	CG9324	Pomp	Protein degradation	0	2	3.09	No														
82	CG3329	Proteasome ?2 subunit	Protein degradation	0	2	2.75	Falls Positive														
83	CG15259	nht	Transcription/RNA Polymerase	0	2	2.38	No (Transcription factor)														
84	CG11246	Rpb8	Transcription/RNA Polymerase	0	1	4.85	No (Transcription factor)														
85	CG1554	RNA polymerase II 215kD subunit	Transcription/RNA Polymerase	0	1	4.01	No (Transcription factor)														
86	CG3980		Unknown	0	1	3.92	Yes	2	3	1	3	2	2	2	2	2	2	3	0	0	x
87	CG3758	esg	Transcription/RNA Polymerase	0	1	3.58	No (Transcription factor)														
88	CG1865	Serine protease inhibitor 43Ab	Protein degradation	0	1	3.37	No														

89	CG2924		Protein degradation	0	1	3.2	Yes	1	2	1	1	2	2	1	1	0	0	0	0	0	x
90	CG9366	rho-like	Other	0	1	2.6	Falls Positive														
91	CG13459	cp309/D-plp	Centrosome	0	1	2.43	Yes	1	1	0	0	1	2	0	0	2	1	2	2	1	x
92	CG31302	CG31302	Other	0	1	2.26	Yes	0	0	0	0	0	0	0	0	none	none	none	none	none	
93	CG8302	Cyp4aa1	Other	0	1	2.07	Falls Positive	0	0	1	1	0	0	1	1	0	0	0	0	0	
94	CG31389		Unknown	0	1	0.68	Yes	0	0	0	0	2	2	1	1	2	1	1	0	2	x
95	CG4494	Sumo	Other	0	0	-	Yes														
96	CG32180	Ecdysone-induced protein 74EF	Transcription/RNA Polymerase	0	0	5.8	No (Transcription factor)														
97	CG8151	Tfb1	Transcription/RNA Polymerase	0	0	5.61	No (Transcription factor)														
98	CG2013	UbcD6	Protein degradation	0	0	4.41	Yes	0	0	1	2	none	none	2	0	none	none	none	none	none	x
99	CG32404	CG32404		0	0	4.25	No														
##	CG17321	CG17321	Unknown	0	0	2.35	Falls Positive														
##	CG2854	CG2854	Unknown	0	0	2.24	Yes	0	0	0	0	0	0	0	0	0	0	0	0	0	
##	CG32543		Unknown	0	0	1.86	Yes	0	0	0	0	0	0	0	0	0	0	0	0	0	
##	CG6187	RluA-2	Transcription/RNA Polymerase	0	0	0.37	No (Transcription factor)														
##	CG7109	Mts	Kinase/Phosphatase	0	0	0	Yes			2	2			2	2			2	2		x
##	CG3157	gTub23C	Centrosome	0	0	0	Yes	1	2	2	2	2	2	2	3	2	2	3	0	3	x
##	CG3068	aur	Kinase/Phosphatase	0	0	0	Yes	1	1	1	0	2	2	2	2	0	0	2	1	0	x
##	CG11509		Unknown	0	0	-1	Yes	1	2	0	0	0	0	1	1	0	0	0	0	0	x
##	CG13609		Transcription/RNA Polymerase	0	0	-1.8	No (Transcription factor)														
##	CG5649	kin17	Transcription/RNA Polymerase	0	0	-2.6	No (Transcription factor)														
110	CG1877	lin19	Protein degradation	0	0	-5.6	Yes	0	0	0	0	0	0	0	0	0	0	0	0	0	
111	CG1157	Osi15	Unknown	0	0	-5.8	Yes	1	2	0	0	1	1	0	0	0	0	0	0	0	
112	CG9903		Other	0	0	-5.8	Yes	0	0	0	0	0	0	0	0	0	0	0	0	0	
113	CG15194		Unknown	0	0	-7	Yes	2	2	0	0	0	0	1	1	0	0	0	0	0	

114	CG10955		Transcription/RNA Polymerase	0	0	-7.5	No (Transcription factor)														
115	CG3352	ft	Other	0	0	-9.8	No														
116	CG4320	raptor	Other	0	0	-14	Yes	0	0	0	0	0	0	0	0	0	0	0	0	0	
117	CG2595	RACGAP84C	Cytokinesis	0	0	-22	Yes	2	1	1	1	0	0	1	1	0	0	0	0	0	x
118	CG33206	l(1)G0168	Cytoskeleton	-1	0	-1.5	Yes	0	0	0	1	0	0	1	1	1	1	0	0	0	x
119	CG4162	lace	Lipid metabolism	-1	0	-1.7	Yes	0	0	0	0	0	0	0	0	0	0	0	0	0	
