# Supplementary material for: A Genome-Wide RNAi Screen to Dissect Centriole Duplication and Centrosome Maturation in Drosophila
Source: PLoS Biol. 2008 Sep 16;6(9):e224. doi: 10.1371/journal.pbio.0060224 (PMC2535660; doi:10.1371/journal.pbio.0060224)
Supplement: Supplementary file 7 [file pbio.0060224.st002.pdf]

Supplementary Table 2. Excluded Genes Primary Screen

	GeneID	Symbol	Mask	Plate	Well
1	CG12563	CG12563	No Cells	1	F24
2	CG13828	CG13828	No Cells	1	G24
3	CG2859	Taf10	No Cells	1	H24
4	CG9669	CG9669	No Cells	1	K23
5	CG31258	Cenp-C	No Mitotic Cells	2	B17
6	CG31300	CG31300	Scrathes On Plate	2	H17
7	CG33098	CG33098	Scrathes On Plate	2	H18
8	CG3193	crn	Scrathes On Plate	2	J19
9	CG8877	prp8	No Cells	3	B20
10	CG31611	His4:CG31611	No Cells	3	C10
11	CG31613	His3:CG31613	No Cells	3	E10
12	CG5844	CG5844	No Mitotic Cells	3	F14
13	CG31618	His2A:CG31618	No Cells	3	I10
14	CG10498	cdc2c	No Cells/No Mitotic Cells	3	P01
15	CG30374	CG30374	Scrathes On Plate	4	C09
16	CG14675	CG14675	Scrathes On Plate	4	C10
17	CG5925	desat2	Scrathes On Plate	4	D08
18	CG5862	CG5862	Scrathes On Plate	4	D09
19	CG32696	CG32696	Scrathes On Plate	4	D10
20	CG14589	CG14589	Scrathes On Plate	4	E08
21	CG30378	CG30378	Scrathes On Plate	4	E09
22	CG14684	CG14684	Scrathes On Plate	4	E10
23	CG30392	CG30392	Scrathes On Plate	4	E11
24	CG5931	CG5931	Scrathes On Plate	4	F11
25	CG6015	CG6015	No Cells	4	P01
26	CG12284	th	No Cells	5	M14
27	CG6058	Ald	No Cells	6	I10
28	CG10484	Dox-A2	No Cells	7	D20
29	CG10370	Tbp-1	No Cells	7	J18
30	CG4654	Dp	No Cells	8	F24

31	CG1395	stg	No Cells/No Mitotic Cells	8	J05
32	CG2807	CG2807	No Cells	10	I06
33	CG3523	CG3523	No Cells	10	L09
34	CG10923	Klp67A	No Cells	11	G09
35	CG5940	CycA	No Mitotic Cells	11	H10
36	CG2925	noi	No Cells	12	M06
37	CG10084	swm	No Cells/No Mitotic Cells	13	H05
38	CG7269	Hel25E	No Cells/No Mitotic Cells	14	B21
39	CG16941	CG16941	No Cells	14	D20
40	CG18174	Rpn11	No Cells	14	J04
41	CG6910	CG6910	No Cells	14	N04
42	CG13613	CG13613	No Cells	15	B15
43	CG12645	CG12645	No Cells	15	F09
44	CG4904	Pros35	No Cells	15	M16
45	CG16916	Rpt3	No Cells	15	N02
46	CG4264	Hsc70-4	No Cells/No Mitotic Cells	16	B13
47	CG1322	zfh1	No Cells/No Mitotic Cells	16	K06
48	CG6686	CG6686	No Cells/No Mitotic Cells	17	B11
49	CG6476	Su(var)3-9	No Cells/No Mitotic Cells	17	B24
50	CG6711	Taf2	No Cells	17	J11
51	CG8636	CG8636	No Cells	17	N04
52	CG6671	AGO1	No Cells	17	N05
53	CG8309	Tango7	No Cells/No Mitotic Cells	17	N08
54	CG8392	l(2)05070	No Cells	17	N12
55	CG18591	CG18591	No Cells	18	D23
56	CG9548	CG9548	No Cells	18	F06
57	CG1249	CG1249	No Cells	18	G22
58	CG6493	Dcr-2	No Cells	18	I04
59	CG8601	mus312	No Cells/No Mitotic Cells	18	I09
60	CG8609	MED4	No Cells	18	K09
61	CG7398	Trn	No Cells	18	N02
62	CG11981	Prosbeta3	No Cells	19	B06
63	CG18869	CG18869	No Cells	19	C07
64	CG6413	Dis3	No Cells	19	M21

65	CG12000	CG12000	No Cells	20	G12
66	CG12254	MED25	No Cells	21	F12
67	CG11295	l(2)dtl	No Cells	21	G15
68	CG8264	Bx42	No Cells	21	J02
69	CG7439	AGO2	No Cells	21	M22
70	CG7109	mts	No Cells	22	M07
71	CG7597	CG7597	No Cells	22	M09
72	CG3949	hoip	No Cells/No Mitotic Cells	22	P19
73	CG11132	DMAP1	No Cells	23	H14
74	CG11700	CG11700	?	24	G17
75	CG3572	vimar	No Mitotic Cells	24	I01
76	CG2163	Pabp2	No Mitotic Cells	24	L17
77	CG2674	M(2)21AB	No Cells/No Mitotic Cells	24	P07
78	CG18789	CG18789	No Cells/No Mitotic Cells	25	E22
79	CG13281	Cas	No Mitotic Cells	25	I19
80	CG18787	CG18787	No Mitotic Cells	25	M16
81	CG7292	Rrp6	No Mitotic Cells	26	G17
82	CG10800	Rca1	No Cells	26	J05
83	CG3780	Spx	No Mitotic Cells	26	N14
84	CG2038	CSN7	No Mitotic Cells	27	A15
85	CG6905	CG6905	No Mitotic Cells	27	J17
86	CG31805	CG31805	?	27	K14
87	CG10712	Chro	No Cells	27	N09
88	CG31992	gw	No Cells	28	G01
89	CG6620	ial	No Mitotic Cells	28	L12
90	CG13288	CG13288	No Cells	29	B22
91	CG5363	cdc2	No Mitotic Cells	29	E02
92	CG3938	CycE	No Cells	29	M09
93	CG6694	CG6694	No Cells	30	D23
94	CG15697	RpS30	No Cells/No Mitotic Cells	31	A23
95	CG3119	CG3119	Scrathes On Plate	32	I09
96	CG7708	CG7708	Scrathes On Plate	32	J08
97	CG9323	CG9323	Scrathes On Plate	32	J09
98	CG7979	CG7979	Scrathes On Plate	32	J10

99	CG31099	CG31099	Scrathes On Plate	32	K07
100	CG5648	Prosalpha6T	Scrathes On Plate	32	K08
101	CG3123	CG3123	Scrathes On Plate	32	K09
102	CG5850	CG5850	Scrathes On Plate	32	K10
103	CG15772	CG15772	No Cells	33	A06
104	CG7850	puc	No Cells	33	D02
105	CG1554	RpII215	No Cells	33	I10
106	CG14129	CG14129	No Cells	35	C09
107	CG4494	smt3	No Mitotic Cells	35	H13
108	CG17299	SNF4Agamma	No Cells	35	J01
