# Supplementary material for: A Genome-Wide RNAi Screen to Dissect Centriole Duplication and Centrosome Maturation in Drosophila
Source: PLoS Biol. 2008 Sep 16;6(9):e224. doi: 10.1371/journal.pbio.0060224 (PMC2535660; doi:10.1371/journal.pbio.0060224)
Supplement: Supplementary file 8 [file pbio.0060224.st003.pdf]

Supplementary Table 3

CG	Name	Forward Primer	Reverse Primer	dsRNA
CG8233		taatacgactcactatagggCGAATAAGAGCCCGTTGATCATCAG	taatacgactcactatagggTTGATCATCTGGAGGTAGCTCTTGG	TTGATCATCTGGAGGTAGCTCTTGGTGCCCACGTTGCCCGCTTGACGGATGGTGCCTTGAGCCAGAACGGAACCAGTAGGCTTGATTGTCTGCACATTGACGGTCTTGGCAACCTTGGATCCTGTGGCTGGATCAACCATAATGGGACTACTGGAAGCTGTGGTCGTCACTGCACCTCCGGTGTTTGGGGTGCTCACAATCCTTATAGGGACACGATGAGTACCAGGCGCCACATTTCCCATTGGCTTAATTGTACTCCGGTTGAGGTACACAACTTTATTCTGAGTGGCAAGTGTTCCACCGGGCGATGAATTTAGCAATTGCTGAAGGCCTTTGTTAAGCACAATATTCCCCGGCTTGCTGACGGCAGTAATGCTGCCACTGGTAACCATGCCCGAGGGCTTATTTGCCTCCTTAATGATTTTCTGACTGATGATCAACGGGCTCTTATTCG
CG40397		taatacgactcactatagggGGAAGGAAGCACGTGAAGAA	taatacgactcactatagggAGCTTCAACAACCGCAGAAT	AGCTTCAACAACCGCAGAATTCAAATGCATAGCTGCGTTATTGGAAATCTGCCATTTGTCCAACGATGAAAGAACTCGGGATGTTAAAAAGCTTTTTCTGCCTCTTTTTATAGGGGATAATTCTAAATCTGAACTGGAAATGTCCATATATTCTTGAACAGCAGACAAAGCATCACTTTTGTCACTTTTAGAAGATCTAATACAATCTGTAATACAATCTTTGTATGCCTGACTACCTTTGTCTGCACTATTAGCAACACAAATGGTGGTTTCTCTGCAATTGCGAGCTCGTTCCATTTCTTCACGTGCTTCCTTCC
CG9078	ifc	taatacgactcactatagggATAACCGCAACCAGTCCAAG	taatacgactcactatagggAAGATCCGGTTGTGCATAGG	ATAACCGCAACCAGTCCAAGCATCCAAGATGGGACAGAAAGTTTCGCGCACCGACTTCGAGTGGGTGTACACGGAGGAGCCGCACGCCTCGCGGCGCAAGATCATACTGGAGAAGTACCCGCAGATCAAGAAGCTCTTTGGCCACGACCCGAATTTCAAGTGGGTGGCCGGCGCAATGGTGCTCACACAGATCCTGGCGCTGTTCGTCGTGAAGGACCTGTCGTGGTCGTGGCTCATCGTGGCCGCCTATTGCTTCGGCGGCATCATCAACCACTCGTTGATGCTGGCCGTTCACGAGATCTCGCACAACCTGGCCTTTGGACACAGCAGACCTATGCACAACCGGATCTT
CG2046		taatacgactcactatagggATTGGAGAACCTTAAGCCGGTGGAG	taatacgactcactatagggGAGGGGCAGCTTGTCGGTGTAAATA	GAGGGGCAGCTTGTCGGTGTAAATAACGAAAGAGGTAACAGGCAATTCCATCTGATCCCGCCAACTGGCAACTGCAAAAAAAGGTGAACAGATGGTGAGGTGATTCAACCTTCTCCTTTTCCACTCATCACTCACCGCCTGCTGCAACTCCAGCGATAAAGTTGGGAGCCTCTAGTTCAGTGATATCCTTCTGGTTGGCACCTATGCCGCGGACAACGGCAATGTGATCCCGGTAGAGCTGGATGTCCTCGCTTTTGAACTCGACCTTTGGCTTAAGCGTCAGAGTGATCACTTTCTTGGCAAGCTTCACGTACGGCAGTAGGAGCTCCGCTACCTCGCCGCTACTGGTCAGGTCCTCGTTGATTATGGCCAGCAACTGTTTGGACGAATGACTCCAGTGCAGGGTCGAGTTATTGGCAGGAATGGCGCACACACGCTTTGCATCCTTTTCCAGCAGAGAAGAGGCGAAGTGAGTGACGTTGACGCCCTCAATGACCACCAGCATCTCGCTCTCCACAGGGGCAGCTTCCCCATCGCAATCATCATCAAACTTCAGCTGCAGCCTGCGAATCCAAGAAATTAGGACTACCTACGTAAATAACATTAGATCACGCACTTCTCCACCGGCTTAAGGTTCTCCAAT
CG31175	Dys or CG7344	taatacgactcactatagggTGCTCTGGAGAGTCCGG	taatacgactcactatagggGCCCTGGCCAGAAAGAC	TGCTCTGGAGAGTCCGGCGCCAAGTCCGCAGCACACAACCCACCAGCTGCAGAACGACATGCACTCCCGCCTGGAGATGTACGCCTCGCGACTGGCTCAGGTGGAGTACGGCGGAACGGGCTCGAATTCCACGCCGGACAGTGATGATGAGCACCAACTGATCGCTCAGTATTGCCAGGCTCTGCCCGGAACTTCAAATGGAAGTGCACCCAAGTCGCCTGTCCAAGTAATGGCCGCCATGGATGCCGAGCAGCGGGAAGAACTAGAGGCGATCATCCGCGATCTTGAGGAGGAAAACGCCAACCTGCAGGCAGAGTACCAGCAATTGTGCAGCAAGGAGCAGAGCGGCATGCCCGAGGACTCCAATGGCATGCAACACTCCAGTTCCAGCATGACGGGTCTTTCTGGCCAGGGC
CG8640		taatacgactcactatagggCCAACCAGGATACGGACGGCACCTAC	taatacgactcactatagggCGCGAACAGCACGATCGGCCAGCTCCTC	CGCGAACAGCACGATCGGCCAGCTCCTCGGGAGTGGGATGCTCCTGGATGTAGCGGATGGAGCGCAGGATCTCCTCTGGGATTGGTGGTGGAGTGGGCAGGAGGTTGGACTGTGGGTGGAAGCCGAACTCATCGGCGGTGTACACCACCTGGACGGGAATACCCTCGGGAGAGATGTAGGAGTACGATCCGTGAGCTTCGTGTCCGGCCAAACCCTCCTCCTTGATCTGGATACCGCTGGCCTGCTCGATATCATAGGCGTAGGTGCCGTCCGTATCCTGGTTGG
CG15770		taatacgactcactatagggACAGATATGCGATCCGTGGATGAAT	taatacgactcactatagggTATTATCCTGAAGCTGCTGGAGTTG	ACAGATATGCGATCCGTGGATGAATTGCTGCAGGAAGCGGAGCGCCTGATCCAACAGCAGTTGCGTCTCGGCGGTATTGTTCCAAAACTGGACGCTTCCGGGCCCCCTGCTCCGACCAAAACCCTCGGAGAAGAAGCTGCCACCGGTGGTGATGGCTCTTCCTCATCCCCGCAGTCCTCGCCGCATAGCAGCATACGGCTGAATACACGCTATACCCATCGCATCGAGCATTCGCCTGCTCCCTTGGCGCCAAAAACCGTCGCAGTCAATGGCAGAGTCCGGCCCAGATCCGGTTCCAAATCGTCGGCCTCGCCAACATCGGTGCAGACGCCGCTAGAAAGCAGCAATGGATCGGCTTTGCCGCCTTCGTGGATAACCTGCCATCGGCGTCGGTCATTTCCGAGGAGTCCACGCCCAAGGACATGGACATGGAGAACCATACGGCGGCCATGGCAAAGCTGCAACTCCAGCAGCTTCAGGATAATA

CG4140		taatacgactcactatagggCAGCATTGAACGCATGATTCTGTGG	taatacgactcactatagggCCGGACTCGAAAATGGGAAAACTCA	CCGGACTCGAAAATGGGAAAACTCATGCCAGTACGACGGGCAATCACCGAGGCCAGTTCCTCGGAGTTGTTCTCCTTGCGCGGCTGGATGTATCGGTCCAGCAGAAGGACCGTGGCCGTATTCCGACAATTGGACACAGTGGCGGTTATGTGAAGCGGCGTCTGCTTGCCGTCCGATTCCGCATTTACATCGGCGCCAAACTGCAGCAGCAAATGGGCGCAATCCGCATTGTTCCACTTGCAGGCGGAGTGCAATGGCGTCCATCCCAGCTCCGTGCGGGCGTTGGGATTGGCATGGTACTGCAGCAGCAACTTGGCCATATCCACAAAGTTATTATAGGCAGCTCGGTGCAGCGGCGTGTATCCGTCGTTATCCTTGGCGTTCACGGTGTCTGCGTCCAGTTTTAGGATTTCCCGCACCTCGCTGATGCGGTTCTCGTTCACCGCCCACAGAATCATGCGTTCAATGCTG
CG32176		taatacgactcactatagggATCGCAGGAATCAGCTAGACCAACA	taatacgactcactatagggCGATACTTCTGGCTTATTACTTTCC	ATCGCAGGAATCAGCTAGACCAACATCTCCATCCCAGCCTGATAGCAGCAGCAAGGAGCAGGAAGCGCCCCGCAAAAGAATCAAGATCAAAAGAAACTATAGGCGGACTTCAAAGTCGCCAGAAAAGGAAGTCAAACCTCCAACATCTACTACTTCATCCGAAACTTTGGTTGCATCTAACGATTTAAACAAACTGCCACGTTCCAAATCACGTTCAGCCTCACCAGATGTGATACCTATACAAACGGAACCCGAAACACTGCTGATCAGCGACAGTACGGATGATGAGGCCACCAAAAAGAAGACAACTGAAACAACTACAACTAAGCAAGTCGAATCGGACCCACCAGCCCCAGTCGTTGAGAGCGAGCCAGAGGAGGGAGAAGTGCGCGATGAACCCGAAGAGCCAGCTAAGGTAGTTGACCCAGCCAATCCAGATGAGCCAACAGAGTCAGAAGCAGCAGCTATTTCGAAAGAACTGGAAAGTAATAAGCCAGAAGTATCG
CG1135		taatacgactcactatagggCTGCCAAATCCTTGCAAAACCATTG	taatacgactcactatagggATAGCAAAGGAGTGCCGTCGATAAA	CTGCCAAATCCTTGCAAAACCATTGGCTTCTTCTCAAGCAGTACACCCTGTTGCCGGATCAGTCAGTGAAGCCTATATATGGCACGGATCAGCAGCCACTCAGCTTCTCGGATGCAGAAGATCAAATCTTCGAGCACGACCTGAACGAGCCACGTGACGAAGCTCTCGAGATGGAGCGAGCTCTGGCAGATCGTCGCAACAAACGTAATATTCGCCTGCTGGAGAACGAGCTCTCGCGCTGGGCCGTCCTGGTGGATTCCGTGCTTAGTCCGACCGCTGCCTCCGAGTTCGACAACCAGACACTGGCCTGCTTGTGTGGCCGCCATGTGCGGTACCTAATGCGTTCCAAGGAAATAACATTTGGTCGCGATGCCAAGGACTGTGTGGTGGACGTGGATCTGGGGCTCGAGGGACCGGCTGCGAAGATCTCTCGCCGCCAAGGAACCATTAAGCTGCGCAGCAATGGGGATTTCTTCATCGCTAACGAGGGAAAGAGGGCCATCTTTATCGACGGCACTCCTTTGCTAT
CG9697	PGRP-SB2	taatacgactcactatagggGCTCTCGTTCTATGTGGATTGACTC	taatacgactcactatagggTTGGCAATGACCCACCACGGAATAG	GCTCTCGTTCTATGTGGATTGACTCTGGCCTTGGGCCAGATTGTTCCCCGCAGCTCCTGGTGTCCTGTGCCCATCTCGCCGCGAATGCCCAGGCTCATGGTACCGGTGCGACTGATTATCATCCATCATACAGTCACGGCCCCTTGCTTCAATCCGCATCAGTGCCAGTTGGTACTTAGGCAAATTCGAGCCGATCATATGCGCAGAAAGTTCAGGGATATTGGCTATAATTTCCTGATCGGCGGGGATGGTCGAATCTACGAGGGCTTGGGTTTCGGCATCCGTGGCGAGCATGCTCCCCGCTATAACAGCCAGTCCATTGGCATTGCTTTCATTGGCAACTTCCAGAGTAAGTCATCCCATTCAATTACCCTGTGTCATTTTCTTTAATACTATGTCTGTATTTCAGCTGGATTACCTCCTTCGCAGATGCTCCAGGCTGCCCGAACCCTCATCCAAATCGCTGTGCAGCGTCGCCAGGTTTCGCCCAACTATTCCGTGGTGGGTCATTGCCAA
CG30164		taatacgactcactatagggCATTGCTCTGATGAGCGTGT	taatacgactcactatagggACGTGGAAGCCATTATCCAG	CATTGCTCTGATGAGCGTGTTCGCCATCCTGGCCCTGGTTCCCCTGATTGCCACGTGTGCCTGGTGCTGCGGTCGTCGCTCCACCCAGGAGGAGGTCGACCACATACGGAATGCACCCTGTCACATCCGGGACGAGTCGCTGGAGGAGAGCTTACGATGTCGCAAGAGCGCCGGACTGGTCACCCTGTGGGTCGTCTTCATCCTGCTGACGTGCGTTCAAACTTAGAGCTTAACTTTTGAGGGCAATGGAGAGTAAATGCCGGGGAAACCCATTTTGCAGGCTGAGCGACTTCAGCATTTTTACGCCAACAGTCGCTTGTCCGCCGGAATCGAGATGACGCCCGAGATGGTCAAGTCAGCCGTGCACGACGTGGAGGTCTTCCTGAAGGACACGCATCTACAAATCAAGCAAAAACTGGATAATGGCTTCCACGT
CG17295		taatacgactcactatagggCATGCGAACCAAAGGAATTTCACAG	taatacgactcactatagggATCCAAGTGGAGTACTGGTTCCTTA	CATGCGAACCAAAGGAATTTCACAGCGCGACGCGTTTCTGTGGCCAAACTGTCGTCTGCACCGCCGAATGTGCTCGTCGAATCCTGCAACGGTTCTCATGCGGATAACAGTTCGCCGGACAGTCCGAAAGCCAAAGAAGGTGCAATGACCAACGGGAAAAACCCGGAGATCAAGTTAGCCGTGCCCAAATGCAACACCACCGTGCGCAAGCAGAACGAGGCCCATATCATATCCAACATTAAGCTGGATATGAACCTTAATAAGGATTCTAACTTTATCGCCCCACAGGTTAATTAAGGTTTTCAATTATGTATACTGTTTTATAATAACATATTCTGTAACTACCCCAAAGATCACCAAAAAGATGAACTTCGCTCCGAATCGTGTTGTATTCGGGGTCCTGGTGCCTCTCAATGTCAACGATTCTGTGCTGGTGCCGCACAATAGAAAACCGGATGCCTTAAAGCACACATCGAAAGAATCG□ GAATCATGCAAGGCCATAAGCGAGCCCCAGCTAGCCGACTATGC□ GGAGAAAGTAGATCCCATGATCATGGCTGTTAAGGAACCAGTAC□ TCCACTTGGAT
CG32177		taatacgactcactatagggAATGCACAACTGCCGCCAGAAATAC	taatacgactcactatagggTAAAGCGCGTATGCTGATCAGGAAG	TAAAGCGCGTATGCTGATCAGGAAGCCAAAGGCACAGATCAGCCACCAGAGCCAGGAGTTTTCGTGCGAGGCCAAGTCCGTCGAGTGCTTCACGATCAGCGTCCATTTAGCCAGCGACAATCCGAATCCCGACAATGCTCCATATCGGGCGGCGATTGTGTGACAGAAACAGGTGAGCATCAGGAAGCCGATCCAGTTAAACAAAAAGGCCACAATGAAGGCCGTGATGAACATGATGTCCGTGCCGAGCAAGTTGTCGGTGGTGGACAGACTATTTTCCGGATCGCTGGCATCGATGGCGATGAACGTGAGTGCCGGCTGGGCAGATCGCACAGCAGCGGCTCCATCGCGCAGCGGATTTGGCGGCAGTAGCGGATTCGGCGGCGGCGGTAGCTGCTGCGAGGAGGGTAAAGTCAAAAAGGCGGGCGGCAGCTCTCCGTTCAGCGACTTTTCCATTTGAACCTCCTCGTACGTGGGCAACTTCCCGTGTATTTCTGGCGGCAGTTGTGCATT

CG18572	r	taatacgactcactatagggGGCTGCTCTACTTACAAAATGAAGT	taatacgactcactatagggTTCGGATTGATGAGCACTGTTTGAA	GGCTGCTCTACTTACAAAATGAAGTACGGCAATCGAGGACACAATCTGCCCTGCCTGCACCGAGCAACTGGACGTTGCCTGATGACGTCGCAGAATCACGGATACGCGGTCGATTTGGAGCAGTTGCCCGACGGCTGGTCGGAGCTATTTGTGAATGCCAACGACGGCACCAACGAGGGCATTGTCCATGCCAGCAAGCCCTACTTTTCGGTTCAGTTCCATCCGGAGCATCATGCTGGACCGCAGGACACGGAGTTCCTGTTCGATGTCTTTATGGAGAGCATTCAACAGAAGGATTG ACCATTCCGCAGCTGATCGAGCAGCGACTGCGTCCGACTACGCCTGCCATAGATTCGGCTCCAGTGATGCCGCCAAAGTCCTAATCCTGGGATCCGGTGGACTGTCCATTGGTCAGGCTGGCGAGTTTGACTACTCCGGATCGCAGGCCATCAAGGCCATGAGGGAATCAAACATTCAAACAGTGCTCATCAATCCGAA
CG7186	Sak	taatacgactcactatagggCGCTATATGAACCACATCGCCAGAC	taatacgactcactatagggAACATAAAGGGATGGCAGAGAACAG	AACATAAAGGGATGGCAGAGAACAGCCTCCAGGGTAATTCGTTCGTGCGGGAGCTTTTTTAATAATTTGTTTATCAGGTCCTGCGCCTCGTAAGACAAGTGAGCCGGCATAATGTACTCGGACATCACAACTTTGTTAAGGGTTGACTGCACTGCATCGGTTTCGAAGGGCGGGCGTCCAACCAGCAGGGTGTACAGCATGCATCCAACGCTCCAGACGTCCGCGGGCAGTCCGTGAGACGTTCGCGATACCACCTCAGGCGAAATATAGTTCGGAGTTCCACACATGGTCATATGGCGCTCATCAGGTCGCTTTAATTGGGTGGCCAGTCCAAAATCGGCTATCTTGACGTGCATTTCCCTGCTAAGCAGTAGGTTGGACAGCGAAATGTCGCGGTGCATGATGTTGTGGGAGTGCAGGTACAAAAGTCCCGCCACCACCTGCTTCAGAATGGAAGCAGCTTCCGTCTCTGTGAAGGGTCTGG□ CGATGTGGTTCATATAGCG
CG10641		taatacgactcactatagggGTGGCTCAGTTTGGTTGGTT	taatacgactcactatagggCTTCTTCGAGGCGAAAATTG	GTGGCTCAGTTTGGTTGGTTTTAAAAAAATATATATTAGAAATTGAACGTTATATTTTGTTACTTGATAAGTTGTGATGGGTACTTCAAAGATCCAAAACAAACATTAGTCAAAGATAACATTCAATTCCCAAGATGTTTTTGCCAGCAAATCGAGTTTTTATATGTATGGGGCGATAAGTATGAATATAATTAGAGTATGTGATGCTTGAAAAATGCTTGTACGAAGCGTATCCTCTTCTCCCGGGATTAGTTCCACATTCGATTAACCTACGCCAGATAATTACTCGCTAGACTAAGGGCGAACCTAAGTCCTCTACTGGAAGATCGCTGCCCGCTGCTGGAACTGCTGACGCCGCTGGGCCCGCTCCTCCTCCTCTCGCCGGCGCTCCTCCTGCTCCGCCCGAATCTCGTCGTGGAACTTGTTCGTCCGCAGCTGTTGCTCAATTTTCGCCTCGAAGAAG
CG11052		taatacgactcactatagggCCAAGAAACGCAGACTGAG	taatacgactcactatagggTGCACAATGCCGAACACT	TGCACAATGCCGAACACTTCGAATTGGCAGCTGAAGACCGGCTCATCGCTTCTGTCCGATGTTACTCTTTTTTGGAACTCTGTGTTCGAAATTCGGTTGGGCTCCTCGCTGCCTTTTTTGGATGGTGTACCTTTATCTGGTGTCGAAGATGGAAGAGTTACCCTCCTACGTTCACCCTCAGTCTGCGTTTCTTGG
CG11327		taatacgactcactatagggCTGCACGTGAAATCAGGAGA	taatacgactcactatagggAAGAAAAGTGGAGCCGGAAT	CTGCACGTGAAATCAGGAGACATTGCCTGTCAATCCATTTAGTTCAATTGCCACAGTTAG□ GCGAACACACTTTCGCTAGGGAACGTTTTGATCACCGAAATAAGGGAAAACCCATGTTGA□ AAACATTGATATAATATTTCACGGATGTCGAACTAGTTAACCAGGTTAAATTCAGTCAGT□ CTTGCGTAAATTTATAAAGGGTAAGTTGGAGCACAGTGCTCTACCAAATTGTGCTGACCT□ CATTCATTTATGCGTCCAAATAGCCACGATGGCCAGCCGCAATGTGCAAGCAACTCCATC□ CCAGGAAGAGCTGAAGCGGACTGAGAACAACAAGAATGTGAAAAACTCCGAAGAATGTCA□ ATCAATGGAAAACGATGTGGACGCGGTTGTCCCATCTTCGTCGGAGAGTGAGCTGCGGCC□ TCGCAGTTCGGGAGTTAGGCCCACTAAAATTCCGGCTCCACTTTTCTT
CG9045	Myb	taatacgactcactatagggGTGTTGCTCAAGCAGCTGGTGGAGA	taatacgactcactatagggCCACCGGACTTGATCAGCGTGATGA	CCACCGGACTTGATCAGCGTGATGAGATGGGTTCGCGAACTCTTCAAATCGCTACCAGATGCATTAACCGATCTGCGTTCGACGTCATATTTGCGACGCATTGTTGAATTCCAATGGTTTTTAATGGCGTTATCGGTGCGTCCGGGCAGACGTTTGGCAATCTTTGCCCACTGGTTGCCCAGCTCCAAGTGAGCCTGGTAGATAATCTCGTCCTCCTTTTCGGTCCAGGCCGTTTTCTTGATATTCGGATTGAGGTGGTTGTGCCATCTCTCGCGGCACTGTTTCCCAATTCGTCCGTTCAGATATCGTGCAATTAGTGTCCATTTCTTGGGCCCGAAGTTGCGCACCAGCTTAATAACCATGTCGTCCTCGTCCCGCGTCCACGGGCCCTTGATTAGCTCCGGATTGAGGACTTTGGCCCAACGCTGCTGCACTTGCTGTTCCAGGCGATCCTTGAAATGCGGTCCGATAATCTCCCAGTTCTCACCGTGCGTCTCCACCAGCTGCTTGAGCAACAC
CG12678		taatacgactcactatagggTATAAGCCGGACACGCTC	taatacgactcactatagggCCGTTTTGGCTGGACAATC	CCGTTTTGGCTGGACAATCTTTCTTTCTCCGTGGCGAATCGAGGCGTTGGGCCCGTACCTTCCGAGGAGGGCGCTCTGCGACTGGGAAACGCCGCCGGCGGCGGAAGCGGACGCCGCGGTGATCGCCGCAAGGCCGCTGCGCAACGAGGTGCAGGTGGCTAGATCCCGGGGGTGGGCTAGCCCGTAGGCGCCAAAGGCCGACTGCGAGTGGACCGCATAGTCGAGGCCGGGTAGCTCCAAGGAGGCGATCGGGTGAATGCGGGTCAGTGAAAGCGTGCTTTCGAGCACGGTGCTCAGGAGCGGGGCGCTGCGCAGGGATCTCAGGCTGCCGCGGCGCGTGAGCGTGTCCGGCTTATA
CG4786		taatacgactcactatagggACCACAGAAATGGCGATATTTGGAT	taatacgactcactatagggCATTGCAGAATCAGAACTTGAGGTT	CATTGCAGAATCAGAACTTGAGGTTTCTTGTTCTTCTGAGTCGGTTCTGAGATCCGGCGACTCGGAAGTGGCAAATTCTCCAAGTTGTTCATAACTCGAATCCGTACTTGTCCTCTCTGTGGAACTGGATAATTCACTTGTGTTCGGAGTCGGTGTGGCCAGTTCAGATGATGTAGATGAGCTATTGGGTGAACCGCTGGTGTCTGGAACGGAATCAGAAGTATCTGTTGGCACAGTGGAGCTCCACCCTGAACTGGAGGATTCGCTTTCGCCCAAAATTGGTGCTGAAGTGGTGTTTCTATACTTGTGATTACCATCGTCATAGATACTGTTACTCACACCCAGCTCGTCCGGACAAACCGGACAGCACTGTCCCTTGACCTCAACGGGATTGTTGCACCGATAATAATGATTGCACTGAACAGCATTGCGGGTGACATATCCACCTTCGCACATGCATGTAGTACAACCATTGTTTTCCATCCAAATATCGCCATTTCTGTGGT

CG6235	Tws	taatacgactcactatagggCTGATCCGGGATCCACAGAATGTAA	taatacgactcactatagggCACACTTTGATGCTCAAGTAATCCC	CACACTTTGATGCTCAAGTAATCCCTGGAGATCATGTAGCGACCCGAGTTGCTTAGCTTCACATCGCTGATGGAGCTGATTATTTCGCTGAAAAAGCTGCGATTCGTTGGATTCTCGGGCTCCTCGAACTGTTTGCTGTGCCGGTCGCATAGCGCCGCCGAACGCATATCACATAATCGTATTGTGCCCTTAGAGCTCGAGTAGACAAACACATTGCACTCGGTCGGATGGAATTCGGCCGCGGTGATCACCTCTGTTAGCTCCTCCATGTTGGTTGGCTTGATGTCGACAATGTTGTAGCTCTGGTTGACCACCTCCAAGTGCCACAGATTGATCCGCAGGTCGTCGGCCGACAAGAAGGTCTCCTGATCCGAATTAACGCTTATTGAATTGATGTGATAGGTGTGTGCATTGGCGAAGGTGCGCCGTGGGGAGGCCTCCACCAGCAGTGGTATCTGCTTCACGGATGGCACTCGGAGGGCCGTTACATTCTGTGGATCCCGGATCAG
CG2791		taatacgactcactatagggTCTCGTCATCGCCGAAGGACTTCAG	taatacgactcactatagggGTAGGATGCAGTCTTGCGCAGAGAG	GTAGGATGCAGTCTTGCGCAGAGAGGTGATCTGCCTGTAGGTCTCCTCGGTAACGTTTTGATAAGCCACAGCATCCACAGCCACCACGGGAACACCGGGCAGCAGGGATAGGAACATGGCCAGAGCAGATGGATCCTGTGGGGTGTCGACATAGACGTCCGCTAAGTTCCACTGCAGCCAGCTGTCCGTGCCCGATTGGGCGAGTGTGTTATCCAGTTCCTTCTGTAGCGACGTGTCTGGCTTAGATTTGCTGAGCACCTTTACAAAGTTTCCGTACATAGGCAGATCGATGCCGTATGCTCCAGAATCTCCGCTCAACTGGTACACCTGTGGTTCCATGACATCTTCGGCAACCGAGAAGAAAGCCTCGTCGGAGGCATTCTTCACGATGCTCAGGTAATCATAGAGCACATCGCCCAGGCCGGATAGGAAAGTTGTTTGTCTGTGGGAGTAGAAGCCATATTCATTGGGTCCCAGGCTGAAGTCCTTCGGCGATGACGAGA
CG1668	Pbprp2	taatacgactcactatagggCTCCTAGTGGGCATCCTCTG	taatacgactcactatagggAAGGGCGCTTGTGATACAGT	CTCCTAGTGGGCATCCTCTGCCTGGGCGCCACCAGCGCCAAGCCGCACGAGGAGATCAACAGGGACCACGCCGCCGAGCTGGCCAACGAGTCCAAGGCTGAGACGGGAGCCACCGATGAGGATGTGGAGCAGCTGATGAGCCACGACCTGCCCGAGAGACACGAGGCCAAGTGCCTGCGCGCCTGCGTGATGAAAAAGCTGCAGATAATGGATGAATCCGGTAAGCTGAACAAGGAACACGCCATCGAGTTGGTCAAGGTCATGAGCAAGCACGATGCAGAGAAGGAAGACGCTCCCGCCGAGGTGGTGGCCAAGTGCGAGGCCATCGAGACACCCGAGGATCATTGCGACGCTGCCTTCGCCTACGAGGAATGCATTTACGAGCAAATGAAGGAGCATGGACTCGAGCTGGAGGAGCACTGAGAACAGATTTGAGACCCATGACGACCCCCCGTTACTGTATCACAAGCGCCCTT
CG15524	Sas-6	taatacgactcactatagggAGGGAGGTCCACGACTATTATCTTC	taatacgactcactatagggCACACTCTGGCTTTGCAACAGGACA	AGGGAGGTCCACGACTATTATCTTCAGTTTGTTGAAATTCGGTCCTTTAAGAACCTAGTTCACCTTAGCCTGCCTTGTCGTTCTGCTC CCTTGAACACCGTACTCTTCTACATAAACAGCATGCTGGAAGCCTCCCACAAAAAGCAATATATTCTCGAGCAAAGTATGCAGCAAAT GCAGGCTGAAATCAACGCACAACGCGCGCATGCGGAAAGGTTGACCACGGAAAACACAAATATCAGGGAAGCTTTGGCCGAAAATACT CGCATCTTAGAGGAAAAGCATGCTGCCGAGGTCCATCAATACCAGGAAAAACTGTCCAAAATAAACGAGCAGCGCAGCAATGAGCTGG AGAGAAATCGTAGAGCGATCTCCGGCTTTCAGGCCCAATTGGACAAGGCTTCGTTGGAGAAAGCAGAACTAAAGTCGGCCCAAGAGCA GGCCGAGAAACGATGTCAAACCCTGTCCGAGGAGTTGTCCTGTTGCAAAGCCAGAGTGTG
CG13162	Ana3	taatacgactcactatagggCCTGGATGTTCAAGAAGATAGTTGG	taatacgactcactatagggTGCTGACAGAGTAAAGCGCTGAAAT	CCTGGATGTTCAAGAAGATAGTTGGTGAGCGGCATTCGTGTGAA GTAATTAGCTGCTATGTTGCCATATGCACCAAAATGGTGGCCATGAAGCCAGAGTGGTGTGCCGCGCTCTGCGGCCACAGTTTGATGAGTGGTCTCAGCGATGTGATGAAAAAACTCAAGTCGCAGGCGTCCTGTTCGATTCCGCTTGTGGAACTGTGTGCATCCCAAATTTGTGAATTGTACTCGATGTGCTACACTAATAACTTTGAGTTCCTTAAGATGACGATTTGTCGAGATTCTGTATTCTTGCAGAATTATTTAACGCTGATCAACGATGTTTTGAGTTTGGAATGTCCCGAGTACATGGTGATTCCCCTATTAAAGATGTTTCTCATATTCTGCACGGACTCGAATTCGAACTCATTTCTCATCGAACAGATAAAGAATAAGCCATCTCTATTCATGGATTTCTTCCTGTTTGGATTGCATGTGAAGCTTATTAATTCGCCATTTCAGCGCTTTACTCTGTCAGCA
CG1483	Map205	taatacgactcactatagggCGAAAGTCACTAAGCAGTAATGTAG	taatacgactcactatagggCGAGCTGTGAAAGATTTTGTGATAG	CGAGCTGTGAAAGATTTTGTGATAGTTGCGGTAGCAGTGGAAGTGGTGGTAGAGCTGGTATTCTTGCCCAATCCATTGGATTGCTGCTTGTTAGGGCTACGAGTAGAAAATGAAGGGGTACTTGTGCTAGGCACCTTTCGCACAGTGGTGGTGGAGGAAATGGTGCTGCGCGGCGACAAAACTTTAGGCTTGACTGGGGCTGTCATTGTGGCCGCAGGGCGAACTTTTGTTGAAGCAGCGCTGCCAGGAGCAGATCCTTTTGCATTTGTAACTGGTCGTCGAGCAGTTGAGGAGGCTACAGATCCTGTTCCACTTGTTGCTGGTCTGCGAGCGTTGGTACTGACCAAAGGGCGAAGTGTTGTCCTGGTTACATTATCAGATGCAGTACCACTTGCAGTCGGCTTGTTAGTTATGGTCTTGGCCCCTAAAGTTACCTTGCTCACTGGAGCTGTAGCTGGTCGTGTGCCGCTTAACTTGGTTGGTGGTTTAACTGTTGATCCTACATTACTGCTTAGTGACTTTCG
CG18217		taatacgactcactatagggGTAACGTGACACTGGATTCGGTTGG	taatacgactcactatagggGATTGGATCTGCGGCAGGATTTGGA	GATTGGATCTGCGGCAGGATTTGGACTGAGCGGCTTGCGCGAAATGCTCTGCTGCAAATGACGAAAATCCGTTGAGTTTAGCACAGAGTCCGTGAAACTCATGGTGTCGGTAAGCTTGTTGGAGCCGCACAAAGTGCGCTCCGCATGGGAGTTGCTGCTCTGGTTCTCCTTGTTTTCCCCATCGTCATAATTGTTTCCACGACCATGCTCCCACAGGTGGTTGACAACCTCTACAGTGGATGTATCCGAATTGAGATCTATGTCCCTTAGCGCCATGTCGATCGCGGAGAGACTTAGGATCTCGGAGCTCGACGGCGGACGCTTGGTCTCTTTGGCTGCCAGACCATTCCTGCTCTTTAGCGCAGTTTCAATCCGATCCTGTTGCATCTTACGCATAAGATTGCGTCCGAGATTCGAGCCAGTTATGGCATCCGTTTTAGTGTATGTTTTATTGTCTGGCTGTGGGGTCTTCTCGCCAACCGAATCCAGTGTCACGTTAC

CG6631	Ana1	taatacgactcactatagggTGATCACGAAAGTGCGCGAGGAGAA	taatacgactcactatagggTGATCAGCATCCAACCGCGACAGAT	TGATCACGAAAGTGCGCGAGGAGAAGCAGCGACTGGTGGAGGTCACCCTCACATCGAACAGCGATGGCGAGCGGCAGTCCACTGAATATCTTGAGCTTCCAACAGGACAACAGCAAACCAGATGCCGGACAATTTCCGATCGCAGCGATTCTCAAACACCTTCGACCTCGGAAGCCTTGCCGTTGCAGAAGCACAAGCCGACAGCGGCCAGCAGGGATAGTGGCATTGCGGACTCACGTCCAATTACGGCACAGGGTCAGGTTTGTATCGATGTGGAACCCATCTCGCTTGGGTCATCAACGCAGAATAATACAGCACGCGGACGAGTAAGGGCACCTCCGGCCACGATTCGTCGGTATAGTCCTCAACTAAACGCCGAGGATCTGGCGCACGAGCTGTCGACCATTACAGAAGTGGAAACGCCAGGACAATCACATATTGTGGCCGCCACACCGGTGCCCAAGCCATTCCCTAGTTTCGATCAGTATGCCAAGGAACTGCACTTAGATCTGTCGCGGTTGGATGCTGATCA
CG9476	[alpha]Tub85E	taatacgactcactatagggCTCGGACTGTGCGTTTATGGTTGA	taatacgactcactatagggGATAGAGCGCTTGGTCTTGATGGT	GATAGAGCGCTTGGTCTTGATGGTGGCAATGGCTGCGTTCACATCCTTGGGCACCACATCACCGCGGTAGAGCATGCAGCAGGCCATGTACTTGCCACGACGCGGATCACACTTGACCATCTGGTTGGCCGGCTCGAAGCAGGCATTGGTGATCTCGGCCACGGTCAGTTGCTCATGGTAGGCCTTCTCCACGGAAATGACGGGAGCGTATGTCGCCAGTGGGAAATGAATGCGTGGGTAGGGCACCAAATTGGTCTGGAACTCGGTCAGATCCACATTCAGGGCGCCATCGAAGCGCAACGAGGCCGTAATGGAGGAAACGATCTGGCCAATTAGGCGATTGAGATTCATGTAGGTGGGTCGCTCGATATCCAAGTTGCGCCGGCAGATGTCGTAGATTGCCTCGTTGTCAACCATAAACGCACAGTCCGAG
CG8472	Cam	taatacgactcactatagggAACGGCACAATTGACTTCC	taatacgactcactatagggACCGTCGCCATCGATATC	AACGGCACAATTGACTTCCCTGAATTCCTTACCATGATGGCACGCAAAATGAAGGACACCGATAGCGAAGAGGAGATCCGAGAAGCCTTCAGAGTGTTCGACAAGGATGGCAACGGCTTCATCTCGGCGGCCGAGTTGCGTCACGTGATGACAAATCTGGGCGAGAAACTCACAGACGAGGAGGTCGATGAGATGATCCGGGAGGCTGATATCGATGGCGACGGT
CG13467		taatacgactcactatagggACGACGACTATATGGCCAGCTTGAG	taatacgactcactatagggCGAGTTCTCGCTCATGTTGTCCAGA	ACGACGACTATATGGCCAGCTTGAGTCAGGCCCAGACCGGCGAGGATGAGGTCGATGGCGGTGGAGAGGCAGCTGGCAGTAACGACTCCGTTCTGGGCGGCCAAACAATGCCACAAATTAGTCCGAACGCACCGCTCGAGGGAAACGTTTCGCCGCCACCGACGCAGCCCCATTTGGATGATAATCTGCACAACGGGAACGGGAACGGGAACGGCATCCACAGCAGCAGCAGCAACATTAACAAGCACGGCAGCAACATCAACGGCAACAACGATAGCAACCGCAGCAGTCCTGCGAAGAGTCCCAAGCGCCCAGTGTCCTCCAGCTTCCGGATTCAGAACACCAGCCGGTACGATAATGATGAGGAGGACTTAGCAGCCAGCGAGCAGGTGGCTGGTGGAGATCGCCAGGTGCCGGATTATTGGCAGCAACGGAACGGTGCGACCACTGGTGCAACCAGTGGGCGGCAGAGTCGCGCCATTAGTCCCGGGTATCTGGACAACATGAGCGAGAACTCG
CG12306	Polo	taatacgactcactatagggGGAGTTCGAATGCCGCTACTACATT	taatacgactcactatagggTCAGACAAGAGCTGGGCAAGAACAT	GGAGTTCGAATGCCGCTACTACATTTACCAGATAATCCAGGGCGTTAAGTACTTGCACGATAACCGCATTATCCATCGAGATCTGAAGCTGGGCAATCTCTTCCTCAACGATTTGTTGCACGTGAAGATCGGGGATTTCGGGTTGGCCACGCGCATTGAGTATGAGGGCGAGCGAAAAAAGACCTTATGCGGAACGCCCAACTATATAGCCCCGGAGATCCTCACCAAGAAGGGCCACTCCTTCGAGGTGGACATCTGGTCGATTGGCTGCGTCATGTACACACTGCTTGTGGGCCAGCCGCCGTTCGAAACCAAGACTCTGAAGGATACGTACTCGAAAATCAAGAAGTGCGAGTACCGCGTGCCCAGCTACTTAAGGAAACCGGCGGCGGATATGGTCATCGCCATGCTGCAGCCAAATCCGGAGAGCCGCCCGGCAATTGGTCAGCTGCTGAACTTTGAGTTCCTCAAGGGCTCAAAGGTGCCCATGTTCTTGCCCAGCTCTTGTCTGA
CG4832	Cnn	taatacgactcactatagggTACATGAGGGCATTGGAGCGAAACA	taatacgactcactatagggCTCGGCCTTTAAGTTCTCTACAGTC	CTCGGCCTTTAAGTTCTCTACAGTCGGAGCCATCGAAGTAGCGGCGTGCAGCTCTTCGTGGGAATTGAAAGTTTTGTGGCTTGCATCACCTTCGGTGTACAAGATCTCGGATAGATTGCACAGCTGAGCGAGGCTTTGATCAGCCAAGGATGTAGCTGTTATATTCAGAGTCATATTAAGACTCTTGGAAAGATCCAAGCTGCGATCCACCGCCTTACGCATGGCATTGCGTCGATCAGCGGCCAACACGCCAAGAACATCTTTGTGCTTCAGCAGAGAGTTGAGGAAACCAGCCAGCTCTTCCAATCGGTTTGTCAGCACACTGCAGACATCGACGCATTCAAGACGCCACTTATCCGCCGTCTCGACTTCCGTGCGAGCTTGTTGAAGTTCCAAACGCAACTGCCCAATCACAGCGCTGTTCTGTTGATCGTCTGCCAGGCGCTGGAAGAGCACACCACCTTTCGCTGCAGCAGCTTGTTTCGCTCCAATGCCCTCATGTA
CG4162	lace	taatacgactcactatagggTCGATCAACTACCATCCGGAAAAGA	taatacgactcactatagggCTTTCCTCTGAATCATCAGCACACT	TCGATCAACTACCATCCGGAAAAGAAGTCGATCCCCAATGGGCATGCCTCCATTCTGGATCAGGAGTCCAAGCCCAACCAGGCAGAGGGCAAACTTTCGCCGGAGCTGGAGCACTTCCAGAAAACCTCCTTTGAGGAGGTGCCTTTGCACACGGCCTGCCTTACGTATTTGGGATTCTATCTACTGATGATCCTGGGCTACATCAACCAGTTGCTCTTCGTGCCCAAAGTGGCCACCGAGAAGGGTCGGGATGGCTATGTAGCCCTCTACGATGCCTTCGAAAGCTTCTACTCGAGGTACGTTTACAGGAGAATCAAGGATTGCTGGAACCGACCCATCTGCAGTGTTCCGGGAGACGAACTCACTCTAAAGGATCGCGTAACCGATGATTATGGATGGAGTTTCAAGTTCACTGGAACGGAGACACGTTGCTTAAATCTGGGATCCTACAATTACCTGGGATTTGCCGCCGCTACTGGACAGTGTGCTGATGATTCAGAGGAAAG
CG33206	l(1)G0168	taatacgactcactatagggGCTGCTCAACGAACTGGACAAATAC	taatacgactcactatagggTGGAGTTTGTGCTGATCCTGACTGA	GCTGCTCAACGAACTGGACAAATACAAGCGCAACAAGCTGGAAACCATTGAGGAGCATCACGAGGAGACGATTGTGCAGCTGGAGGCCCAGTTGGAGGAGGCCAGACAGAAATTGGAATTGGCTTCTCTGTCTAGCCAACAGCAGATGGAAACCCATCTGATATCCAGTCCAGAAAAAACTCCCGTAGATAGCGAGCTTCTGGCCAAAATGGAGCAAAAGGAGCAGGAATACTTACAATTGCAGGAGCAGTTGGCCTTCGCCAAAACGGAACTGGACAAAAGAAATAAACTGCTGGAGAGGAATGGGGAACAGCTGACTAAACAGCAGCAGCAAAATCAGGCAGATCAAAAGAAATTGGAAGAACTTTCACAACTGCGGGAGACATTGCAACGTAGGGATGAGGACCTAAAGGAGCTGGAAGAGCAATTAAGCGCCGTTAGACAGGATCTAGATGAGAAGTCCATTCAGATGAAGATCAGTCAG□ GATCAGCACAAACTCCA

CG1877	lin19	taatacgactcactatagggCCTGACAGATCTCAAGTCCATCCTT	taatacgactcactatagggTCCTCTGAAGCTTGACGGTGTATTC	CCTGACAGATCTCAAGTCCATCCTTGAGAACCATATACTCCACCAGGGCACGGAGGCCATTGCCAAGTGTTGCACTACCGACGCGGCCAACGATCCCAAGACGTACGTACAGACCATCCTGGACGTGCACAAGAAGTACAACGCCCTCGTTCTGACGGCGTTCAACAATGACAACGGATTTGTGGCCGCTTTGGACAAGGCGTGTGGAAAGTTCATAAACTCGAACGTGGTCACGATCGCAAACAGTGCTAGCAAGTCGCCCGAATTGCTGGCCAAGTACTGCGATCTGTTATTAAAAAAATCCTCGAAAAATCCCGAGGACAAGGAGCTCGAGGACAATCTTAACCAGGTGATGGTCGTGTTCAAGTATATCGAGGATAAGGACGTCTTCCAAAAATATTACTCCAAGATGCTTGCTAAGCGCTTGGTTAACCACACATCGGCTTCGGATGACGCCGAGGCTATGATGATCTCCAAACTGAAGCAGACATGTGGTTACGAATACACCGTCAAGCTTCAGAGGA
CG4320	raptor	taatacgactcactatagggTTGGGACGTGGAAAGGGAACTGAAA	taatacgactcactatagggGGGTGAAAATACAGATACGTGGGGT	GGGTGAAAATACAGATACGTGGGGTGACCGATCTTGGGACCAATGAAACCCTCGTTCGTCCGCAGCACCTTCTGCTGCCGTCCGTCCAGCGAGTAGATCACGATCTTGGAGGCATTGCCGCAGGCCACCGTGTCCTCCAACTGGTGGCTGGTTATGGCCGTCACATCGCCACCGGCGTCCCACTCGTACACAACATCGAAGGGGTTAATGGAGGACTGGCTTCCCCGCATGTCCGCCACACTGATCCGACCTTCGGTGCAGCCGGAGACCAGCACCAGTCCGTTTGCCCGCAGGCTGGCGTGGAGGATGGGGGCACTGTGTCGTCGATAAACGGAGATCCCGGCGTCCTGCGGCGCACAACGCTTGTCAAACAGTCGCACACTGCCGTCGTCGCAGCCGGCGAGGATCACATCGGAACGCATGTTGGGCAGGTAGGGTGACAGCACCCGGGCGCAGGTTTCGCTGCGACCCAATGGAATATCTGCCAGTTTCAGTTCCCTTTCCACGTCCCAA
CG2595	RACGAP84C	taatacgactcactatagggGCCGATAACTTCTGCGTAGC	taatacgactcactatagggGCAGTTCCCCACGTTGTAGT	GCCGATAACTTCTGCGTAGCTGACTCAAGCCGAAGTGGCCTCTCTTCGGAAATTGCCAACTGACCAGAACAGCTGAATCAATTCGCCAACTTTATGATTAGTGGCTCTGGGAGTCGGACGCCCTCAAATCGACTTTATTTGTCGCCCGTTCGCCCCACGATGCAGAACAAACGGAGGCTTCTGCGGGAGTACAGGTCCTACGATGACCTCAGCGAGCACTACCGCATGTTTGGATCGCAGTCCCTGGACTCGCTGCAGGATCGCGTGGACATGAATCCCAGTGGCTGCGATGGCTTATCCACGGACGGACTCGACTTCTGCTCGCAGTCCCACTCGGGGCTGCTGAGGGAGCACAATTTCAAGATTAAGTCCTACTACTACAACGTGGGGAACTGC
CG6187	RluA-2			
CG15194		taatacgactcactatagggGAAGAAGGTGACGGTGAAG	taatacgactcactatagggCCACGCCGCAGTAAATG	AAGAAGGTGACGGTGAAGGAGTACGATTCGCAGGATGAGGCCAGCACCGAGCTGCTGGAGAAGCTCAAGCATCTCGACCGTCGTGTGGATGCCGCCGAGGGTGGTGGCTTGGCCAAGGGAACCGCCGGCGGACGAAGGAGCACCGCCTGCTGGCAGTCGATGAAGTGGAAGTCGGCGCTCAATCACATCGGACTGCTCGTCTCCCTGTCCATTTACTGCGGCGTGG
CG11509		taatacgactcactatagggTGGTGTTCCAAGATGTTCCA	taatacgactcactatagggGAAATTGTCGCCAAAGGAAA	TGGTGTTCCAAGATGTTCCATTCGGCTCCTTTCTACGCTCAGGATCAAGGACAAGCCAAATCAGTTAAATATCTTATCTAGTTGGAAACACTCACTCGTACACAAACGCTTGCATACTTATAGCTGTAGAAATATAACCCATACCAATATCAGTAACTACACCCACACCCACAACCACAGCCACAGCCACACCGATCGAAGATCAAGTCAGAATTCAAAAGGGAAAACCAACAGCAGTAGTATGAATACAAAAAGCAACAGACAGAAGTTTCCTTTGGCGACAATTTC
CG1157	Osi15	taatacgactcactatagggTCAACATGCTGAACCGCTTAGACAG	taatacgactcactatagggTAGAAGGGCTGGGCCAGACCATAGT	TCAACATGCTGAACCGCTTAGACAGCGAGGAGTCCGTCGCGCTGTTCGGCGGTCTGAGGATCGACCGGTCGGAGTCGGGCCGTAGCTTCGGTGCATCCAAGGCCGTCGAGTCCTTCGAGGACCGCGCCGAGCGCTACCTGGAGACCCACGAGCTAAACCTCTCCTTCTCCGGCGATGAGCAGGACGAGAACTCCGAGAACGAGTACACCGGACGCGCCATGGATGGTGAGTGATCTTGGTTTCCTTTATTTGAAAAACAGAATATAGTTTAATCCAAAGAAAAAAAAGCTATTTAAGCTACTTGCTTTTAAAGTTTTCATATTGCGTTGAATATCTTAGATACTTAATATTAGGTCAGAATTTAAAAATTTGTATGTTTTTGAGAGACATAAATCGATAGAATGGTAGTAAATACGTGAGTTGATAGCCAAAGAATTTTGAAATAATTTTAAACGAGTTATAAGTTACATTTTTATTAATTACTACAAAAACAGAGGCATTCGTGCGCATACGAGAATAGCTTTGGTAAACCGTCCTGGCTCTCAAGTCGTTCCTGTTCTGTACGGTAAACGAACACTTCAAAGTCTACTTACTAAGGCTAAACGTACTAAGGTCCACTCTGCTGTGCAGAAAGCCAACTGCCAAAAGCAAAACTACTAGCATACTCAGTTGTGCTGACGGGAAGCGAAGTGAAAGCTTTACCTCGACTGCCAGGCCTTGAATACTTTTAGGAGCATGGCTAAAACCCCGATTTCTCTTTACTTTCTCCCTCCCGGAATAAACAGAGTCCCGCAGCAAGCGCATGAAGAAGATGCTGCTGCCCCTTCTTTTGGCCCTGAAGCTGAAGAAGGCCGTCGTTGTCAAGATTATGTTCACCATCATCAAGTTCATCTCGCTGAAGGCTCTGGCCATCTCCTTC[illegible]TGGCCCTCATCCTGGCTGGTGAGTTTACTTTTAATGATCTCAGGAATAGCACTTTTTACCCACGTTTCCCGCACAGGTGCCACCTTCTTCAAGGATCTGTTGGCCAAGAAGAAGGAACACATCACCACTGCCTACATCACCGGCAGCCCGCTGAACGCCGACATCGTGCACTCCGACTGGAGCCGCAATGGTCAGGCAGGTGCCGCCGATCTGGCCTACAACCACTATGGTCTGGCCCAGCCCTTCTA
CG10955				
CG9903		taatacgactcactatagggTAAAAATATCGGACGGCTGC	taatacgactcactatagggCAAAGCGAACTCCAGTCACA	TAAAAATATCGGACGGCTGCTTGTGGCGGTGGGAGTGCTGATGATTCTGGGATTGCAAGGTGTCCGATCTGAGTATGGCGACCTGGCCGGCAATTGGCTGGTGGATTATGATCGAGACGAGCTGAATTTTTTTGAGGATTCGACTTGGAGCGTGGATTTGCACATATACAATGTGTTACCGAAGGACTCTTCGCTGGATTACTACTTTGTGGTGTACTCCAAGGATGATCGGAGGGCGTCATCGCCACCGCTGGAGATAAAGAAGGAGGATTTCGATCTGTTGGGCAGCTGGCGGGGAGCGCTATATGTGACTGGAGTTCGCTTTG

CG31389		taatacgactcactatagggCCCGAAGAAAGAAAGGAAGG	taatacgactcactatagggTTAGTTTCAAGCCCCACGAC	CCCGAAGAAAGAAAGGAAGGTAACCTTAGTCAGTCAGTCAGTCGCTGGATTGTGGGCCAA AAGGGGAAGGGAAATGGGAAAATGGGGGAAAAATGGCATGAATGGAACGCGAGGCGCTCA TGAATGGCGTGTGATGGTTTTCGCCGGTGCCCAGAAATTTGCATATCGCATGCAAGGCGA TGAGCAGCAGCAGCGGCAGCAGGAAAACCACAAGCAGGAAGAAGGAAGCCAAGCCTACAC CTGTGCAGAAACACCGATACATGTATCAGCTAATTACACGGTTAGCTGCCCCATGTGGTT GACCCTGAGCGAAGCGGAGTGCAGTTTGTGCACCAGAAGAACCCTTCTCCTTCTTCGAAT CTACCTCCACGGATCTTGGAGCAGCCGCAATTGGACACCGCCAGTTATTGCCCAACGGTT AGACCATAATCCAATACCCATGCCCAATGTCGTGGGGCTTGAAACTAA
CG13459	cp309/D-plp	taatacgactcactatagggACTCTGCTCAGCTGGATTCAAACAT	taatacgactcactatagggGAGCAACCAGCAGTTTAATCTCACT	GAGCAACCAGCAGTTTAATCTCACTGCGCAGACGATCTCCGAGTTCGGATTCAAAATCAGCCCGTGGAGATTTCCTGGGCGAGTCGCCCGGCGACTTCAGTAGCAGGGAACTGTTTCTTCTACTTTCCGCTCGTTGCAGCGATGTCAATTGCGTTTGAAGACGCTCACAACGTTCCCGTTCCTGATCGAGCTGTTTGTGGAGCTCGTCCTCGGCATCAAGAGCTGTCTCCAGTCGTTTCCTCATGGCTTCTATGATAATCACATCCTGCTCTTGAATATCCTGCGAATGCTGTTTCTCTCCCAGTAGCTCCTTGCTCATTGCTTCGCATTGCTCACGCTCCACGGCCAATTGAGCACGCAGGGCTTCCAACTGATTTAGTAGTTCCCGCTCGCCAGTGAAGTTCTCGTCGTCGGTTCCATTGCCAGTTCCAGGTAAAGTCTCCTCCTCCGTTTGGACACCTTTCTGCAGCTTCTTGTCCAGATTGTTTTCCTCCTCGCTTTCAATTGCTTTAAGGATGTTTGAATCCAGCTGAGCAGAGT
CG8351		taatacgactcactatagggAGTGCGTCCTCCATGGTAAC	taatacgactcactatagggACCTGAAGCTGGAGCACCTA	AGTGCGTCCTCCATGGTAACCCTCCATTAGTTTCAGGACAATCTTGTTTCGGTGCTCCACCAACTGCCGGGAATTGCGTACCCATGCCGGTGCGTCTTCCTTCCGCACAATCTCCGGTAGAATCTTCACCACCAAACCGCTCTGCTCAGTTAGCATATGCAGCAGTAGTGCGTTGGTGCGGAATTTGTTTGGAAGTTCATCCTGGTATCTTAATGCACCCTTCCACAGAGTGATAGGTTCATTGAGCACCAGTTGCTGGCCACAAATGTATATCTCTTCCATACGCTCGTTGATAAAGTCTAGCAGTAGAGAGTTCTTCACTTTGGCTCTGCCCGCTTGTAGCTGCGGTGCATCAAAGTTAAGGCAGTGCAGCAGCTGCTGATACCGTTCCAGAGGAATCTTCCCAGAGAATCCGACGAGACCGTAAAACATGTTAGTGCTCCAGTAATCCGATAGGTGCTCCAGCTTCAGGT
CG2924		taatacgactcactatagggGTGCAGGCAACCGATCGCTTAATGAA	taatacgactcactatagggCATTCTTTTCGTGAATCTGCACCAGA	CATTCTTTTCGTGAATCTGCACCAGAGATTTGAAGCTCTGCTGGGCTCGAGCCAAACTGTATTGGCCCTGGGTCAGTGCCTGTTTATAGAAGGTTGTTAAGAGTATTAAATAAAAAGGTCGTCCATTTTGTCGACTCGAATTCTATGAATTTAACATACTTTGGTAGCCCCGAATTGGATGCGCGCCTTTCCCTTGACGAGAGTGGCTGCAATTTGCATGATAACCGCTTCCACGGTATATGCCGAGCTCCAGCCCTGTTTGGTCAGCAATTCCATGCAGATGGCACCACCGATGAGCACATAGCCGCCTTGTAAACACAAGAATCATTTAAATAAACCTCCATGATTTACTTATACTCAGCAAATCCCACCTGAGATAATCGGATGGACAACGCGCACAAATGGCGGCTCGAAGGGATACGTCTCCTTGAACAGGATGTTGAGCAGTATGCTGTCCTTGCCCTCCTTCTCCTTGAGCATTTGCAGATCACTGTGCAGCGGACTGTCCGGGTCGACAGACTTGAGGCGAATGTTCCATTCGTAAATCGACTCATTGACGAGCTCAATAGAATACATGTTCTTTTTGAAGGCGTCTGAGCGATAAATGTCGCGTAATTCCTTCATTAAGCGATCGGTTGCCTGCAC
CG8302	Cyp4aa1	taatacgactcactatagggCGCCGTGGCTTTCACTCTCTTTCTG	taatacgactcactatagggAGGTGGCCGCAATGGTTGTCTTTCC	CGCCGTGGCTTTCACTCTCTTTCTGCTGACACAGAATCCCGAGTGCCAGGATCGCTGTGTCCTGGAGCTGGCGACCATATTCGAGGACAGTAATAGAGCTCCCACGATGACGGATCTGCACGAGATGCGTTACATGGAGATGTGCATCAAGGAGGCACTGCGTCTGTATCCCAGTGTCCCGCTTATTGCTCGTAAACTGGGCGAAGAGGTTCGCCTGGCAAAGCACACATTGCCCGCCGGCAGCAACGTTTTCATTTGCCCCTATGCCACCCATCGTCTTGCCCACATTTATCCCGATCCGGAAAAGTTCCAGCCGGAGCGATTTTCGCCCGAGAACTCCGAGAACCGTCATCCCTACGCCTTTTTACCCTTCAGCGCTGGACCGAGATATTGCATTGGCAATCGATTCGCCATCATGGAGATCAAGACCATAGTGTCGCGCCTGCTGCGTAGCTACCAGCTGCTTCCTGTAACCGGAAAGACAACCATTGCGGCCACCT
CG10863		taatacgactcactatagggCGCAAGACTCTGCAGAACTTTGGTC	taatacgactcactatagggGAGGACTTTGGCAATGGGATGGTTC	GAGGACTTTGGCAATGGGATGGTTCCAATCTCAATCTATTTCGATAAACATTATGTTAATAAAGAGTTTTTGTTCTCATTAAACCTAGTTCGTAATCCCTACCAGATATCGGAGCACCACTTGGGCGGTGGACTTCTTGTACTTGTCTCCAATTGCCTGCACCTTGGCATCATAGATGTAGTTGGGCGTCTTTTCTGCTGGATTGGGTCTGCCCAGGGGACAGTAGGCGGTCACTACAATGTCATTCTTCTTGCACAGAGCAATCAATTTCTTCTGGTTCAACGCGGGATGACATTCGATCTGGAAAATGGTCTATTAGGAATATGAAACCCAAGGCATAACCACAATTGACGCACCTGATTGTGGATTGGCTTGATCTTGCAGTTGGCCAGCAAGCGAGTCAACTGTTCCGAGTTGAAATTGGATACGCCGATGCTCTTGGTCAGGCCCAGTTCCACGAGCTTCTCCATTTCGCGCCAGGTGTCTAGGTAATCGATATCGCTGTAATAGCACAAATTTGGTCATTAAGAGTCAAGCAAATACAAACGAAGCACACCCGCATTAGAAAAGTAATTGACTAAATTAAAAACTCACTTGAGTTCCACCTCCCCCTTGGCATCGGTGGGCATCATCTCGTTGTCGCCGCGGTAGACGTAGGAGTAGGGCCAGTGCATCAGGTAGAGATCCACGTACTGCAGACCAAAGTTCTGCAGAGTCTTGCG
CG9147		taatacgactcactatagggCAAGCATTGATCAGGTGGAG	taatacgactcactatagggTCGGACTAGTTTAGCGCCTC	CAAGCATTGATCAGGTGGAGGGCCTTCCGTTGCTCACTTTGTATACCAGGGAGCCCTGTCCACTGTGCGACGACCTTGTAGAGCAGTTGGAACAGGGCTTTGCAGGTCAATACCGTCTGGAGAAGGTTTACATCGACCGAAAGGAAAACGTTAGGTTCCTCCGCCTCTTCAGGCACGACATTCCCGTTCTGTTCTTCAACGGCCAGTTCCTATGCATGCACCGGCTAAACGAGGAAGCACTAAGAGAGCGGCTGGAGGCGCTAAACTAGTCCGA

CG11312	insc	taatacgactcactatagggGGATAGCATGAAGCTGGACGATGGA	taatacgactcactatagggAACTTGCAAACCGCACTGGTGGTGA	AACTTGCAAACCGCACTGGTGGTGATGGTGTAGTTCTTGTATGCCTCCTGCGCAATCGATTTGTGCTGCAGGGTGCACATCACCTCCGGTTCGCCGGCCCAGCGCTGTTCCGACATCCAACTGGCCACGGGTTCTGGGTGCTTCTTGCCCCGTTTCGCAATCTTGGACCCACTTTTTGGTGTGCTGGTGCTAGCTGTGAAACGCAAGGATCTGACTCTTAAGGGGGAAGGTTGCTGCTGGAGGCTTTCATCCTGCTCGGTGGCTCCAAAAGTCACCGTTTCATTGTTGTACGGCGGTGGCGCCTCACCCAAGTAAACAGTTTCATTGTTGTACGGCGGCGGTGGTGGTGGTGCTATGACCACTTCCTCCTCCTCGCTGGCCGAAGCCGCTTCCGGTTCCGGCATGGGTTTACGCATCAGACGCCTGCGGCGTCTTATGGCCGGACGCGGTGGTGAATCCTCCTCGGCCTCTGCCTCTCCATCGTCCAGCTTCATGCTATCC
CG15259	nht	taatacgactcactatagggTCTCAGGATGCAAGCAAGGCAAAAG	taatacgactcactatagggACTTTCGAACCACTTGGAGCAGATT	ACTTTCGAACCACTTGGAGCAGATTCTGGGGCTAGTTGTTCTCCCGGGCGCTTTCGACCGGAGATGGCTAGCAAGGCCGTATCATTCACCGATTCCAGACGAGCCACCTTTCTCTCCTCGTCGATGTTCTCTGCCCGGCGCTTGCGATAAAAGCCGGTCTCGTCATCCGATGATCCATAGTCGGCAATCTCGAGATCGTTGAGGAACATCATCGTGACCCTCATATCGTTCTTCATCACACAACGTTCGTCGTTGTGCAGATGATAGCTTGTTCGATGCTCGCACAATTCGATAGTCTTCTTGACCACATGTTTCATGTAGGTCTCCAAGGCGGCCAAAAAAGAATCCAGGAGCCATTCTTGCTCGGAAATGTTGATATCATCCGCCTGGTTGCCCTGGGACAAGTGCTTCATTATCTTTTTGTAGATACTGCTGCGGTCAAAGAAGGATGCACGTTGATTTTTCTTTATGGAGACTTTTGCCTTGCTTGCATCCTGAGA
CG17081		taatacgactcactatagggCAGAAGACCACCACAACGACCAAAG	taatacgactcactatagggATTGGAAGCAAAGCCACCGGTCAGT	CAGAAGACCACCACAACGACCAAAGACGGTGTGACAGTGACGCAAACCAGTGGACAGGAGGAGTTAGACAAGATGCAGAATGATCTTACGGCAGCCGGAGAACAACTGGAGTTCTTCAAGCGCAAAGTGGAGGCCAGAAATCGGGAGATTCGCCGCTTAAACGACATGCTAGCAGGAGGGCGTCCTTTAGCGGCTTTGGCCAAGGATTGCTGTTACAAGGACGTGGGCGCCCTCTCCCAGGACATCGATCTGTTGCAACGCGAGAAGAGCGATTTAATGATGCAAGTTCGAGAATTCCAGGACAAGATGCACGATGCTATGCAGCGGGCTCTAAGCTCCGAAGAGGATAAAATAAAGCTCCAAACGCAGCTGGAGGAGCTCAAGGAAGCTGCGTTGCAAGTGGAACAGCAGGCCAATGCGGAAATCGATGCCAAGGAATCGGAACTTAGGCAACTGCAGTTGGAGCTTAAGAAGAAGGGAAAGGATCACCGACTGACCGGTGGCTTTGCTTCCAAT
CG4913	ear	taatacgactcactatagggGTTCCGGCAAAGAGACGAAGTCTAC	taatacgactcactatagggCTCCTGAAGCTCGCTCAAATACTCC	GTTCCGGCAAAGAGACGAAGTCTACTGTCAAGGAACACAAGGGCAAGTCCAAGCAGAACGACAAGACGCTCGAGAAGAACACTGACAAGACAGAAAAGCCGGACAAGACTCCGGACCCCAAGGTGGACAAGTCCGAGAAGTCGGAAAAAGAGGACAAGAAGCGCAATCGAAGAAGCTCACAAAGTAGTAACAGTGGCAGTGGTGCCGGAGTCACCGGTGCTTTTATAGAACCCCCTGTTAAGGCCTCGAAAAAGGAGCAGGGCAAGGCGGCGGGCGAAAAATCCAAGTCGCCCTCGTTGCGTCCACCTAGAGAAACCAACTCTACAGGCACTGGAGGCGGGACGCCCAATGCCGGAGCCGGTTTCCTTGCTCCACCATCATCTTCGCCCAGCAACAACCACAGCGCTGCAGTGGCGGCGAGCTTAAATAATAATAATAATAATAACAACAATAACACGAACAGCAACAGCAGCCATTCGCCCGGTGAGATGACGACACCAGGACAGCTCCAGCTGCCCACGGAGTATTTGAGCGAGCTTCAGGAG
CG13387	emb	taatacgactcactatagggCGGAGTTCTCTCAGATCTTTACACT	taatacgactcactatagggAGATCTTGAATACTTCCACGTCCTC	CGGAGTTCTCTCAGATCTTTACACTATGCTCCTTCGTGCTGGAGAACTCGATGAATGCCGCCCTAATCCATGTCACGCTGGAGACTCTGCTGAGGTTCCTTAACTGGATACCGCTGGGCTACATCTTTGAAACGCAGCAAATTGAAACGCTGATATTCAAATTCCTTAGCGTACCCATGTTCCGCAATGTGACCCTGAAGTGTCTGTCCGAGATCGCCGGCCTGACTGCGGCAAACTACGACGAGAATTTCGCGACGCTCTTCAAGGACACGATGGTTCAGTTGGAACAGATAGTCGGCCAGAATATGAACATGAACCATGTGTTTAAGCATGGTTCCGACACAGAGCAGGAGCTGGTCCTTAATTTGGCCATGTTCCTGTGCACCTTCCTCAAGGAGCACGGCAAACTGGTGGAGGATGCCAAGTATGTGGACTACCTGAATCAGGCGCTCATGTATTTGGTGATGATTAGCGAGGTGGAGGACGTGGAAGTATTCAAGATCT
CG11175		taatacgactcactatagggACCACGCAGCAGATAATGAA	taatacgactcactatagggACTGGCCTCCTCCTTTGAA	ACCACGCAGCAGATAATGAATAGATTCGCCTTTATGAAGCGCTCCATAATGCGATCGTTCCAAAAGTTTTGTATGCTATTGCCGTTGCCCGGCGGCCGCTTCTTCAATGAGGACGACGACTGCTTTTCACTGGCCTTCTGATAATTCATTATTCGTGTTATTCGGTTGTTGCGGTTGTGGTGTGAAGCAAATGTGTGAATGCGGTTTTTCAAGAGAAATTATGCTGGCTAGCGGGAGGGTGGCCTTGGTTCCCCTTTTTCAAAGGAGGAGGCCAGT
CG14617		taatacgactcactatagggAGACCAGCAGGATCAGGATACAAAG	taatacgactcactatagggTCTATGAGCTGACGCTGCTGATGAT	AGACCAGCAGGATCAGGATACAAAGGACACAACGGCAAGGGAGCTCGGCAGTCCACTGGCAGAACCAGCTAATTTTCAGCGTTCGAGAAGTTTCACACTAGAGGAGCCCTCCCAGGTTCTCGTGGAGCACATGGAGAGGGAGGCCCTGGCTGTGAAACCCAGCCAGAGTTTGGCCAACTTCGAGAAGCAGACCATCGAGTCTCAGGCCAAGCGGGTGAACCGTAGTGCAAGGAGTATTAGCAGCAAAATTAGCCACAACAGCTACAATAGCAACAGTAGCGCCGCAACCAAGAGCACCATCGCCACTAAAACCACAACCGCCGGCAGCAGCTGTTCTAGGCGGGATCGCGAGGTGGAGCGTATAATAGAACGGGCGCTGGACGAGCATGGCGCTTTGGAAGCCAGCCGCAAGGCAGGAGTGCGCAACTATCTGCGTGGCCATCGGGAGCGAATGAATCAATTGGTCATTCACCAGGAGAAGGAACGTCGTCGCATGCAGGCCGAGTTTGATCATCAGCAGCGTCAGCTCATAGA

CG5461	bun	taatacgactcactatagggGCAACATGTTCGATCGCACGGTGAA	taatacgactcactatagggGCTCCATTATTGTTGGGCGAACTGG	GCTCCATTATTGTTGGGCGAACTGGCCTTGGTGAAGCTGTTGTTGCTGCTGCTACTGCTGTTGTTACTGCTCACTAGCTGCTGCTGTTGCTGCTGCGGTGTCGAATTGCTGCCACCACTGCTGCTGCTGGACTTGGGTGCCAGCGATGTGGAGGTGGATGTGGCCAGGCTGGAGTTACTACTGGCCCTGGACACCGTTGTGGGTGTCGCTCCAGCTGCCACGCGACTCATCATGTTCAAACGCAAACTTTCGCTGGTGGTGCGCTGCAAAGCCGAATTGCTGCCGGCATTGTTGCTATTCTGATAGATGTTGCTGGGTGGATAGCACACAGCAAAGTGATGGTTGCTGGTCAGTGAGCTGACCTTGGTCAGCTTGCGTATGTTACCGCCAGTGCCACCGATAGTGACTCCACGGGATGGTGTCAGCATCGAGTTCCGACGGACCGGATTGTTGCCAGGTCCGGCATTTGAGGAAGCCGGCTTGAACTTGGCATTCACCGTGCGATCGAACATGTTGC
CG17286	D-Spd2	taatacgactcactatagggCATCCGCAAATCACCATCA	taatacgactcactatagggGTGGAACTGGCCTCACTAC	GTGGAACTGGCCTCACTACCTGCGGGCGAGGAGTTTGCTTTGCGGGCTGCTCCACTCATGAGCTGCCGGCGGTCGGAACTGGTTGTGCTGTCCAGCAGCGGCGACGAGAGACAGCTTCTTGGCGAGGACAGGGGCGATAGATTTTTGGCCAGGTTCGGTGAAATGCCAAAATCACGTTCGGTAGAAGAGGCCACGGCAACGTCACAACCATCCAAGCGAGTGCTGCTTAAGGAGGAGGCTTTGCGCGGAATCAAGCCCATTGTAGCGATCGAAACCCTCTTCTTGTTCACTGGCGTCTTGTCCTCGTCCTCCTCATCGTTTTCATCCTCGGTGCAGGTCAGTGGACGTACGCCGTCGCTACTCGACGGACTTGCGGCTCTTGGAGAAATCTTAGTACGTCTCACTCTGCGGCTAGGCTCCTTTACCGGCGTCGAGGGCCACTCATCGATGTCCGCCTCGGCTTCTGTCTCTACGGGATCGGTGT□ CCGCACGGCTGATGGTGATTTGCGGATG
CG1906		taatacgactcactatagggCTTCCTGCGCATCGACGAGGTTATG	taatacgactcactatagggACGACCTGGTTGGCGATGCTCACTA	CTTCCTGCGCATCGACGAGGTTATGCGGGAACTGCCGGAATTCACCCGGGAGTCGGAGAAATGCGGCGGAACTACCGCCGTGTGCGCCTTTGTCGGCCTCACGCAAGTGTACATCGCCAATTGTGGTGATTCGAGGGCCGTCTTGTGCCGCCAGGGTGTTCCGGTGTTCGCCACGCAGGATCACAAACCCATTCTGCCGGAGGAGAAAGAGCGCATCTATAATGCGGGTGGCAGTGTTATGATAAAGCGCGTAAATGGTACCCTGGCAGTGTCGAGAGCCCTTGGGGATTACGACTTTAAGAACGTCAAGGAGAAGGGACAGTGCGAGCAGTTGGTATCGCCGGAACCGGAGATATTCTGTCAGAGCCGCCAGGATAGCGATGAGTTCCTGGTGCTCGCTTGCGATGGAATCTGGGATGTGATGTCCAACGAGGACGTGTGCAGCTTCATCCACTCGCGAATGCGGGTGACCAGTAACCTAGTGAGCATCGCCAACCAGGTCGT
CG2919	Asterless	taatacgactcactatagggTCGGTCACATTAAGCAGGAGTTGTA	taatacgactcactatagggCTTTGTCTGTGGCGATGTCAGAATA	CTTTGTCTGTGGCGATGTCAGAATAGAGTTTCTTAAGCCGCTCGACCTCGCTCTGGGAATCCCTCAACAGAATCTTGTGGCGTTCCAGCTCCAAGCGCAGGTTGTCTGCAGTTTCTCGAGTTCGCAACTGCCGATTTTCCTGCGAAAGCTGATCGTAATTTCTTTGAAGGCTCTCGTTCTGCTGCTTTAGTTGCTCCAGATCTTCTTCAATCTGCTGCCGTTTTTCCCTTAGTGCATCCCTCTCGGCCACAATGGAATTCAGCTGTGAGTGCACATCTGACTCCTGCTGTGATCTGTGTTTTTGCTCTTCCAACTCCTGCTGCATTGCCTTCACTTTGTTTTCCAACCGCATTTTGCCTTCGTTTAGGGTTTCCAGCTGAACCAGGGATTGGTTCTCCTGGTCGCGCAGCACGCGCAGTTCCTGGTTGCGTTCCTCCAGGAGCTTCTCTAGGCGTCGCACTTCTTCCCGCTTGTTCTTCAGGTTGCCCAGAGCTCGATACAACTCCTGCTTAATGTGACCGA
CG8687	Cyp6a14	taatacgactcactatagggATGCAGTTCCTTGGTCCATC	taatacgactcactatagggGAGACATCCTCCAGCACCAT	ATGCAGTTCCTTGGTCCATCGCCAAAGGGCAAATAGGCCATCGGATGGCGGTTCTTGACTTCCTCCGGGTCGAAGCGACTGGGATCGAACTTCTCCGGCTCGGGATATATCTCCGGATCGTGGTGGATATTATGCACCGGAATCAGGGCAAGAATGCCCTTGTCTAGCACAATATCTGAGTTGGGAATTTGGTAGTCTTTAGTAGTCTCTCGAATGAGATGGGGAAGCAGTGGGTGCTTTCGAAGAGTTTCTGAAAGAGGTTTTGGCTTAAAATCAGTTTGTTTAGCTAAGGCGGATCGGTACTTACCCGACAGCACTTGATCCAAATACGTCATTTGAGCGAGAACGTCGTAATTAAGCTCCCCGCCGTCCACATTGGCTAGCACACTTTCAATCTCCTCCCTCAACCGCTGCTGAATATCCGGTTGCAAGGCCAGCTCATACAGGCACAAGGACATGGTGCTGGAGGATGTCTC
CG15929	lin-52	taatacgactcactatagggGGACGAGTTCCTCTCCATGA	taatacgactcactatagggGTCAAAGATGCCCAGCAGTT	GGACGAGTTCCTCTCCATGAGCGACACGCCGATGTACACGCGCAGCACCAACTACACCAGCAATCTGACCGATGATGATATGGTTAAGATTAATGGTAAGTAGCTGCAAAGTCAATGAGATTTAAGAATAGTATATTAAAGAATAGCATATCCATTACCTACTTTTCTATTCCAGAACTAGCCCAGCTCCCTCCCGAGGATCTGATCGATAAAATAAAGTCAATGCATGATGAAATTTACCAGCTGGGACTGCGTGAGGCAATGGAGATGACTCGTGGGAAACTGCTGGGCATCTTTGAC
CG8231	l(1)G0022	taatacgactcactatagggGCCGATATCTATCTGTCCGAGGGAC	taatacgactcactatagggCGATCACCTTCTTCACACGCTGGTC	GCCGATATCTATCTGTCCGAGGGACTGCATCCTCGCATCATGACCGATGGATTCGAGAAGGCACGCGACAAGGCATTGGAGGTGCTCGACCAGGTCAAGGTGCCCGTGGAGATCAACAAGAAGAACCTGGTGGAGGTGGCCAACACAAGTCTGAAGACCAAGGTGCATCCCGCCCTTGCCGATCTGCTTACGGACGTCTGCGTGAATGCCGTGCTGACCATTGCCAGTGCCGATAAGACCAAGCCCGTGGACCTCCACATGGTGGAGCTGATGGAGATGCAGCACAAATCCGATACCGATACACAGCTGGTGCGTGGACTAGTCATGGACCATGGTGCTCGCCACCCGGACATGCCGAAGCGTTTGGAGAATGCCTACATCCTTACGGCCAATGTCTCGCTGGAGTACGAGAAGGCCGAGGTCAACTCTGGCTTCTTCTACAAGACCGCCGAGGAGCGTGAGGCCTTTGTGCGCGCCGAGCGCGAATTCATCGACCAGCGTGTGAAGAAGGTGATCG

CG10061	)-Sas4 / l(3)s221	taatacgactcactatagggCAGAGATTTCAATGGCTGATCC	taatacgactcactatagggTGGAGTTCATATCAATGTTTCCC	AAGGCCACTGAGGGAAACATTGATATGAACTCCAGCTGCATTCGAACCTTTATGGCGCGCAAAGATCAGCGTCGCCGAGTAGCTGATGACTCCGATCACATTGTAGTCACACAGCAGGTGCGTCAGATGCGATTGCAGCCACAGGTGGACCAGATGCTGGTGCAGGAACTGCAGGAGGGGGACGAGGAGGACACCGAACCGTCCAGCGATCAGACCCTCACAATGACACCCATCCAGGTGGGGAATACTAAAGTGCGAGTGCGTTTCTCCGACTCCAATGACACGCACGAGTACTCCGATGCCACCAGCCTCAACGACGGCTCCAATATTCAGCTCTTTGAACAGTTCAAGTCCGCCCTCTTCCAGGCATTGGAACAGAAGAAGAAGTCAAGTCCCAGCAAGGAGTCAGACGAACCCATAACCAAAGATCTTCAGGAGAAAGCCAATCTCGTTCGCACTCGCCTCGAAGAGCTGGAGACAGAGATAGCCACCTTCAAGGA
CG31484	CG31484	taatacgactcactatagggTTACAGCCAAAAACCAGAAACC	taatacgactcactatagggGATGAGTTCTGATCGCCAGC	TTACAGCCAAAAACCAGAAACCAAAGCCACGCAGAGGACAAAGTAAGTTCCACTAGTCCGTGTTCCAGCTGCTTTTTTGCTCCTGCGGCTGCCGCTGTTTCGAAAGCCACTGATATTGTTGTCAACGATGATGGCCCAAGGCATCAGCCACATGTGTGGGAGCCAGAGCTCGTCCCTCCTCAAAGTGCCCTCGCACATGGTTGGCAAAGCCAAACCTTTATTGCCACAGCTCCATGGAGCAACAGCGAAAATTTAATATCTGGCTGGGGCGTGGCTCCATTGGAGCTGGTCCGTTGTTGCTGGCGATCAGAACTCATC
CG8308	[alpha]Tub67C	taatacgactcactatagggAGCAAGAAGTGCAAGCTGGACTTTG	taatacgactcactatagggCATTTACATCCTTGGGCACCACATC	AGCAAGAAGTGCAAGCTGGACTTTGCCGTCTATCCATCGCCCAAGGTCTCCACCGCCGTGGTGGAGCCATATAACGCGCTGCTCACCACCCACTCCACCATGGACCACTCGGACTGTGTGTTCATGGTGGACAACGAGGCCATCTACGATATATGTAACAATAGCTTGGGTGTGGACAGGCCCGCCTATAGGAATCTGAATCGTTTGATCGCCCAAATAGTGAGCTCCACAACGGCTTCTCTGCGTTTCAGTGGCTCCATGAATGTGGATCTGAATGAGTTCCAGACGAATCTGGTACCCTTCCCCAGAATCCACTTTCCCCTGGTTGCCTATGCCCCACTGATGTCCGCCGAGAGATCGGCCCATGAACAGCATGCGATTACCACATTGACCAATGCCTGTTTCGAATCCTCCAACATGATGGTCAAGTGTGATCCTCGTGCGGGCAAATTCATGGCCTGTTGCATGCTTTACAGGGGTGATGTGGTGCCCAAGGATGTAAATG
CG3131		taatacgactcactatagggCATGTTTCAGGATGTGGGGCTGGAG	taatacgactcactatagggCTGTATGTGCGAGTCCAGGGGAATG	CTGTATGTGCGAGTCCAGGGGAATGTACTGTTGAATGGGGAACTCCTTCAGCTTGGTGATCAGATTCCGAGACATGGTCAGCAGCAGCAGGGAGTAGCAGAAGGACAGCGATGCGGCAGATCCCCTGGTGATGGCAATGCCTACGCCCATAATGTGCCGCAGATCGGTGTGCTCCGCCATGAACGAGTAGTGGATGAAGCGCTCAACGAACAGAACTATGGTGACCACGTAGAAGAGGAACAGATAGAAGATGTTCTGCCGGTTCTCCTCGAGGAACGTGATGTAGGCATCCCATTTGGCCAGCAACCAGTGACGCGGCTTGTCCTGCATCGGTTCGATGTTGAACGAAGTCATCCGGGCCACGTTCGTCGAGGTGTCCAGGAAGTTCTGCTTGGCTCCCTTGCAGTCCAGTCCGATGGCCACAAAGTCACCCTTGTACTCCTTCATCATCAGTTTGAAGTCCTGGTAGGTCAGATGGTTCTTGTGCTCCAGCCCCACATCCTGAAACATG
CG18377	Cyp49a1	taatacgactcactatagggCTCCACATCCAGCACCACCATACTG	taatacgactcactatagggGCAGATCGGGATGACCAATGAGACC	GCAGATCGGGATGACCAATGAGACCGCCCACTTTGGCCATCTTCCCATAGTTGACATGCAGCTCATTCATGACCTTGTCCAAGTCGCTGATTTTGTATGTTCCTGTCGGTCGATGGCAATGGTTCATTAGTTATCCTGGTCGGAGTTACAACGGATGCCAATAATGGCCACTCACCTATTAGTGGAGCGAAGCGCCAGGAGTTGCCAATCAGTGGCAAGGGGTATGGACCCGGTACTTCCGAATAAGGACGCACTGCAGCTGCCGGTGAAGAAGTGGTTTCTGCTGGTAGTGAGCTTGCTGTAGTCCTAGTTGTTGCTACTGATGTAACGGGCTGTTGGCTGTCAATTAGTGCAAATTGTATACAGTGAAAAAAACATATAAGCACCATATTATTACATTAGGTTAGTAGTGCGTTTTATTACTTAGATTCAAACTGAATCTAAGTAGATTTAGAAATGCAAAGAACAAGTCTTCTATTTGTAAAGTTAATTCAAAAATATTTAGTTAAACTTGGCAAAGTTATTCAACATTTTATTTCCATGTAAGCCAAGTCTTTGGTGTGGGAAAATCACGAACCTGACATTTAATTTCTTCGGATTCGACGACTCGCCCGTCCGCAGCCGTTGCATTGCAATTGATTTTTCAATTTCCGTGGCCACGCCCACTCCGCTGCTGGGCGGCGACACGGCACCACCAACGCCCCCGGAATGCGGCAGTATGGTGGTGCTGGATGTGGAG
CG10346	Grip71wd	taatacgactcactatagggGACTGTCTATGATGTGCAGGGAATG	taatacgactcactatagggTCAGCTGGTTTTGGGTGCTCAACAA	GACTGTCTATGATGTGCAGGGAATGCGAAAGATTTTTCATGCCAGCGAAGCCCACAGTGCTCCCTGCCGGGACATCAGTATGTGCACATCCCAACCGGCACTGCTGGTCAGCGTGGGCTACGATTGCAAGATAAATATCTTCGACATTCGACGAAATCGTGCTCAGGCCTCTACGGATCGCTTGACCTACTCGCATCCACTATCGACGGTGGCGCTGAGTGAATGTGGCACTTATCTTTGTGCTGGAAACCTTAAGGGCGAATTAATAGCCTATGATATGAGGAGTACAAAGGCTCCGTTGGCCGTGAGGAGTGTACATGATGCGGCTGTAACACGTGTAGCCTTTGTGCCCGTGCCAAGTGGGGATAACCAGCAGAACGGTAGCCTCAACAGCTCCGAAAATGCAACTGGTTTGGCGACCGAAGCTCCTCGTGTAGAGCGTGCGCCCAGCGATGAGTTGCGTAAATCGATTGCCGCTAATTTGTTGAGCACCCAAAACCAGCTGA
CG3980		taatacgactcactatagggAACTCGGCAAGGATATGCTACTTAC	taatacgactcactatagggTCATCGATTTTGGGACAAGCCAGAG	AACTCGGCAAGGATATGCTACTTACGAAGGCCCAGCTCGAGAACGAGCGAAAGATTCAGCAGCTGCAGATGCAGGCCATCAATGCGCTGTGGAAAAAGGTGTCCACCATGGAGGTGGATCCCAAGGGAATACCTGCTGCTGCGGAACAGGGCGAGGACTCCAATGGCGGAGGGTCTGGCCACCTGAGTCTCGATCAAAATTCAGCCGCCGTGGTAAATGATTTGGCGAAAAGATGCACCATGCTCACCGATCAGGTTCAAATGCTGCAAAGCTCGATTGGAACCATCGTCAACTGCCTCACAATGGTGTGCAATCTGCCGCAGGACGCTATTAAGAAGCAGGCCGAGATCATTGATTGCAGCTCCACTCAGACGGACCTCATTGCCGTACACACGCCGCAAATAGAGGACCTCACCAATTTTCCATTCACCAAAACAAGGCCCTCAACCTTGGCCCTAGAATCCAAGCACGAAGCTGCTCTGGCTTGTCCCAAAATCGATGA

CG8151	Tfb1	taatacgactcactata<br>gggTTAAAAGCG<br>GCTGTTCAGCAG<br>GGAG	taatacgactcactata<br>gggGTTCTGGAC<br>AGGGCCATTGAG<br>GTAA	TTAAAAGCGGCTGTTCAGCAGGGAGCTGGTGATCCTTTGCTAGACCTTAAAAAGTTTGAGGATGTTCCTTTGGAAGAGGGCTTTGGCAGCG<br>TAGCAGGGGACCGCAACGTCGTGAACAGCGGGAATATTGTGCACCAAAACATGATCAAGCGATTCAATCAGCATTCCATCATGGTGCTTAAG<br>ACCTGTGCTAACGTGACCTCAGCGCCGTCAACTATGACCAATGGTACCAATAATGCCAACGGGCCTGTTTCCCAATCCGCGTATACGAACG<br>GGATGAATGGAAAGGGCCAGGCCACGGCCACCGCGACGAAGAGTTCCTCCGATCAGGTGGACAAAGACGAGCCGCAGAGCAAAAAGCA<br>ACGACTGATGGAAAAGATTCACTATGTGGATCTCGGGGACCCTATATTGGAGGGAGATGATTCCGCCAACGGCGAGAAAGCCAAGTCTAAG<br>CACTTCGAACTGTCCAAAGTGGAGCGTTACCTCAATGGCCCTGTCCAGAAC
CG32543		taatacgactcactata<br>gggCCAAATCGA<br>GCTAATCGAGC	taatacgactcactata<br>gggCGGAGCAGA<br>ATGAGAAGGAG	CCAAATCGAGCTAATCGAGCAGGTCAGTGGTGTTCAAACAGAGCCAGATAGTCCGCCCACCATTAAGGTCCTGGTGTTAGAGGCAACTACG<br>CCCGTGTCCGCTGAGACCGCAACAGCAGATCCAGGCGAGAATCTGGCAAAGATGGCCACCGCGAGCAACACCACCACCATCACCACCG<br>ATGATCAAGCAATGCGGGCGGACGACGATGCGGAAAGGGCCGCTGAAATCGAGCAGACGGTTCTGGACGAGCTTTCACCAACCACCGAT<br>GAGTACCAGGAGTTTGGCGATGCCAAGGATGCAGTGGACGACTTTGATAGCATAGACATTGGCTATATACCTCCAGCACCCACCCCGGCGC<br>CCTCCATGGCGCCCCTGGCCGAACTGGACGTGGACACCGTGGACGATGAGCCGTGCGAACCGGCCGCTCCTCCTCCTTCTCATTCTGC<br>TCCG
CG11451	CG11451	taatacgactcactata<br>gggAGAAACGGA<br>ACGTAGGGTGCA<br>AACT	taatacgactcactata<br>gggAATGGGCTC<br>GGCGAATGACTG<br>AATA	AGAAACGGAACGTAGGGTGCAAACTAACATCACTGGTTCGTCAA□ CTTCCAGACCAAGACACACTCTTTTGATGTCCGAACCGATTGAA□<br>GAAGAACTAACTAACGAAGATAAAAGTACGAATGCCAGCACAAA□ TAGAACGTTAAGATCGAGGCGAACCATTTTGATGGCGGAACCGA□ TA<br>GAGGAAGAGGGCAATAAGTCTATCGCTGAAAAAATAGGTCAA□ TTTACTAAAGAATCTTCTGAAGAAGCGGTATTTCAAACTTCCAA□ ATCTC<br>GTCAAACTCTGCTGATGGCAGAGCCTATAGAGGAAGATG□ TTTTCAATCAGTCAAATGATCAGCCAAAGCCACTTCCTATATCT□ AAGGATAA<br>AATCACTAGGAAGACCTCTAGACGACACACGCTTAT□ TACTGCCGAACCCATTGAAGAGGATTTGAGTGTTCGTAAGCCGC□ TGGAAAATTC<br>AAAGGAGTCATCTTTGACCGCCAGAATTATTCAG□ TCATTCGCCGAGCCCATT
CG14098	CG14098	taatacgactcactata<br>gggGCCAACACA<br>CGGGACAAATAA<br>TCAT	taatacgactcactata<br>gggTAGAGGGGA<br>GTTCTCGTCAAC<br>AATA	GCCAACACACGGGACAAATAATCATCGACCGGGACGTGTTGTCC□ CGCGAGGAACACATATGTTTTCTAGCTACACCCAAGAAGGACTT□ C<br>TCCTCACCCCAGTTGGCCAAACAGGCGCGCTACTACACCAAAA□ AGATCTACGTTAACCAATGTACAGCACGGGTTGAACGACTTGCC□ G<br>ATCCTCACCCAATGCGAGTGAGTGACACATACAATTTCTTTAA□ GGACTACCTCTCGCCTCGTCATATAGCGGCTCTAGAGAACCAGA□ TGA<br>AACCCAAACCTCCGGTAGAAGCCATGTCAATGGAGAAGGCG□ CTGGAATACATGGAGGAGGAGATGCGTCAGCGCAAGGCAGCGAA□ GC<br>GTGTCCAAAAGCGACGATGCAAGGGTCTAAAAAAACGAATCA□ TTTTGCGCCAGCGCAAGCAGATGCAAAAGATAATTTGCGTACTG□ TTC<br>GAGGAAATGAAGGACTTTCTACTCAACGATCAGTTTATTGT□ TGACGAGAACTCCCCTCTA
CG8262	Ana2	taatacgactcactata<br>gggGCAAATGCT<br>GCCCAGGGTCA<br>ACTTT	taatacgactcactata<br>gggTGGTGGTCA<br>GAATATCGCACT<br>GGGT	TGGTGGTCAGAATATCGCACTGGGTGCCAGTGGTACTGCTGGCT□ TTTGAAATGGCTTCGGGTTTCGCTCGCTGCTGTTGCTCCAAAAT□ C<br>ATCTCGCAGAGATTCCGAAGCTGTTGCTGCTCATGGCGCACGG□ ACTCCACAAGGGATACCAGAGCCGCCAGATCACTCTTATTGGCC□ A<br>AGTTGCTGACGTCGATGTCGGAATAGACAACCTGGGGCAGTGA□ TTTTCGCGGAGGAGATGGGTTGAAGGGATCGGTCTGTGTGCCAG□<br>CACAGATGGTGAGGACATCTGCGTTGGAGGAGCTGGACAAGCGC□ CTAGATTGATATGCCTGTTGCTTGGGCTCGGCATTATAGCCACC□ A<br>GCATTGCAGTTCATATGATTGGTAGTCTGCTGCGCATCGCTTG□ CATAGTTGCGATCCCGGGGCATTACTTGGTATTCCTTCGAGGAG□ CAC<br>GGACTATTGCTGCTGGGAGCGGTGCGAGGACCCAGTTCTGT□ GGCCAAAATGGGCGAAAAGTTGACCCTGGGCAGCATTTGC
CG31302	CG31302	taatacgactcactata<br>gggAATTCGTACC<br>ACTTGGCAGC	taatacgactcactata<br>gggGGGATCCAAT<br>AAACTCCGGT	AATTCGTACCACTTGGCAGCACTTAAAATGGTCACATTGCGCTTCCTGACTCCTCGCAGG□ ATCGTCGACTATTGTCGTCGTCTGCTGAGCA<br>CGGACCTGGTCACGGCCCTTCGTCCGTTC□ TACCAGGCCATTCGGACGGATGGCCTGGCTCACAATCCGGCAGAGAAGCGGGTTAACGA<br>G□ GAGTCACTACGTCTGCTTTGCTCCCATCTGGAATCCCTGACTGGGCCGTCGGCAAGTTGT□ CTTCCGCCCGATGGCCATTTGGAGTTC<br>TATATTCCATCGGGCTCGGATGAGTCCGGTAGT□ CGGGTGGTTAGTCGACTGGTTAGGGTGCAGGTAGACCGGAGTTTATTGGATCCC
CG2854	CG2854	taatacgactcactata<br>gggGCAACATGG<br>AACGCTACCACA<br>ACTG	taatacgactcactata<br>gggCACGTCCAA<br>GTTCCGGCATAT<br>GAAC	GCAACATGGAACGCTACCACAACTGCCTCAGCATCCTCAAGGGC□ TTGTCCGAGGATCCGAAGGTCGCCGGCCAGCTGGGAGCCCTTC<br>G□ CAATCGTTTTGTGGTCTTCGCACCGTACACTGGTATTAGCTTGG□ AGGGCGATGTAATGCTCTTTTCGGGCGACTCCCCCTCGGGTTGG<br>□ CTGGCCTTCCTCTCCGAGATGCGTTTGCACGAGGAGCAACTGAG□ ACTGGTGCCCCTGTACGAGAAAGCCCTGTCTGCCGTGCTCCTT<br>G□ GCATACGGATTGAGAGGCGTAGGGTTCCGGAGCCGGGCTGTGAG□ GCGCGTGCCTATGCGGGCAGCTTGAGGCGCGTGATCCGTGC<br>GGT□ CAGTGAGCACCTGAGGCTGGAGGATAGTGTCCAGGAGTTGCCGG□ CATCGCTGCAGGACCATCATTTGGCCATTGTGCCGGATAG<br>TGAT□ ATACCGAAGGTTAGCCAAACCGGTCTCTTCTTGGCCCCGGCCTC□ CTGCTCCGGACCCGATCTCGTGGAGTTCATATGCCGGAAC<br>TTGG□ ACGTG

CG32782	CG32782	taatacgactcactatagggAACAGCAGCAACTGGGAC	taatacgactcactatagggGAATGTGGCGCCCACTC	AACAGCAGCAACTGGGACCACCGACCACATCGACGGCCTCCGTC□ GTGCCAACGCATCCGCATCAACTCGGATCCCTGGGAGTTGTTGG□ GATGGTCGGTGTGGGTGTTGGCGTGGGCGTTGGAGTAAATGTGG□ GTGTGGGACCACCACTGCCACCACCACCGCCGATGGCCATGCCA□ GCGGCCATTATCACTTATAGTAAGGCCACTCAAACGGAGGTGTC□ GCTGCATGAATTGCAGGAGCGCGAAGCGGAGCACGAATCGGGCA□ AGGTGAAGCTAGACGAGATGACACGGCTGTCCGATGAACAAAAG□ TCCCAAATTGTTGGCAACCAGAAGACGATTGACCAGCACAAGTG□ CCACATAGCCAAGTGTATTGATGTGGTCAAGAAGCTGTTGAAGG□ AGAAGAGCAGCATCGAGAAGAAGGAGGCGCGACAGAAGTGCATG□ CAGAATCGCCTCAGGCTCGGACAGTTTGTTACCCAACGAGTGGG□ CGCCACATTC
CG2013	UbcD6	taatacgactcactatagggTCAAGAGGATCCACCTACG	taatacgactcactatagggTGGATGTTAATATGGCTGACAC	TGGATGTTAATATGGCTGACACATCGTACGTGGGACTCCAGCGG□ TTCTGCAATATGTCCAAGCATATTCCACCATCTGCGTATACATT□ GGGATGAAATACTTTCGATACAAATCGAACCGTTGGCGGTTTGT□ TTGGGTACTCTTCCGTAAATTCTATGGTTAACTTAAATGTTCCG□ TCTTCGAAGGGTGTGTCGTGTGGACCAAAAATCACAGCATTCCA□ TATCATAATATTATTATCTGTTGGAGCACCCGAGACACCCGTAG□ GTGGATCCTCTTGA□
CG17291	PP2A-29B	taatacgactcactatagggGATCAGCAGCAGGAGCAGCAACAGA	taatacgactcactatagggGCCGCCGAGAAATGTTGTGCCAAAG	CGTTACATGGTGGCCGAGAAGTTTGTTGATCTGCAAAAGGCTGT□ GGGCCCAGAGATTACTAGGGTGGACTTGGTGCCTGCCTTCCAGT□ ACTTGCTCAAGGATGCCGAGGCCGAGGTTCGCGCTGCAGTGGCC□ ACCAAGGTGAAGGACTTCTGCGCCAATCTGGACAAGGTCAACCA□ GGTGCAAATCATCCTTAGTTCCATTTTGCCCTATGTCCGCGATC□ TTGTCTCGGACCCCAATCCTCATGTGAAGTCAGCTCTGGCCTCA□ GTGATCATGGGCTTGAGTCCCATGCTGGGCGCCTATCAGACTGT□ GGAGCAATTGCTCCCCCTGTTCCTTATTCAACTCAAGGATGAGT□ GCCCAGAAGTGCGCCTAAACATCATCTCAAACCTGGATTGCGTT□ AACGACGTCATCGGTATCCAGCAACTGTCACAGTCGCTTCTGCC□ CGCCATCGTCGAGCTGGCCGAGGACTCCAAGTGGCGTGTGCGTC□ TAGCCATCATCGAGTACATGCCTGCTCTGG
CG7109	Mts	taatacgactcactatagggGCAGGAGGTAAAATGCCCGGTGACA	taatacgactcactatagggCGGACCAGAGCAGATCGCACATGGGAC	GCAGGAGGTAAAATGCCCGGTGACAGTGTGCGGAGATGTCCACG□ GTCAGTTTCACGACCTCATGGAGCTCTTCCGGATAGGCGGCAAG□ TCTCCGGACACCAACTACCTGTTCATGGGCGACTACGTGGACCG□ TGGATACTACTCCGTGGAGACCGTGACCCTTCTGGTGGCCCTGA□ AGGTTCGCTATCGCGAGCGCATCACCATCCTGCGCGGTAACCAC□ GAGTCGCGCCAGATCACACAGGTGTACGGCTTCTACGACGAGTG□ CCTGCGCAAGTATGGCAATGCCAACGTTTGGAAGTACTTCACGG□ ATCTGTTCGACTACTTGCCACTGACGGCACTCGTCGACGGGCAG□ ATCTTCTGCCTGCACGGAGGCCTCAGTCCCTCGATCGACAGTCT□ GGATCACATTCGGGCCCTCGATCGCTTGCAGGAGGTTCCGCACG□ AGGGTCCCATGTGCGATCTGCTCTGGTCCG
CG3157	gTub23C	taatacgactcactatagggTGGTTAATGTGCGAAAGACTACTGT	taatacgactcactatagggTTCACTGAAAACTGCATGTAGTCCT	TGGTTAATGTGCGAAAGACTACTGTTCTAGATGTAATG□ CGGCGTCTGTTGCAGCCCAAGAACATGATGGTGTCCAC□ TGGTCCCGACAAGTCAAATCATCACTGCTACATCTCCA□ TTCTAAACATTATCCAGGGCGAGGTGGATCCCACTCAA□ GTGCACAAGTCACTGCAGCGCATCCGCGACCGCAAGAT□ GGCCCAGTTCATTCCCTGGGGCCCCACCTCTATCCAGG□ TAGCCCTTTCCCGCTCATCGCCCTACGTGCAGAGCAAC□ CATCGAGTGTCCGGACTGATGCTGGCCAACCACACCAG□ CATTTGCTCGCTTTTCGAGCGAGCTCTCAACCAGTATG□ ACAAGCTTCGCAAGCGCGGCGCCTTTCTTGACCAGTTT□ CGACGCGAGGACATCTTCAAGGACGACCTCAATGAGCT□ GGACGAATCTCGCGAAACTGTTGACTGCCTGGTGCAAG□ AGTATGAGGCAGCCACGCGTGAGGACTACATGCAGTTT□ TCAGTGAA
CG3068	aur	taatacgactcactatagggACGTGCGCATATATCTGATCTTGGA	taatacgactcactatagggATTAAGGACCAGCAGCTTGGAAATG	ACGTGCGCATATATCTGATCTTGGAGTATGCGCCACAA□ GGAACGCTTTTCAACGCCTTGCAGGCACAGCCGATGAA□ ACGTTTCGATGAACGCCAGTCGGCCACCTATATTCAGG□ CGTTGTGCTCGGCGCTGCTCTATCTGCATGAGCGGGAC□ ATCATACACAGGGACATCAAGCCGGAGAACTTGCTGCT□ CGGGCACAAGGGCGTCCTGAAGATCGCCGACTTCGGTT□ GGTCCGTGCACGAGCCGAACTCCATGCGCATGACACTG□ TGCGGCACTGTCGACTACTTGCCACCCGAAATGGTTCA□ GGGCAAGCCGCACACGAAAAACGTGGATCTATGGAGCC□ TGGGTGTGCTCTGCTTTGAGCTGCTAGTGGGCCACGCT□ CCCTTCTACTCGAAGAACTATGACGAGACCTACAAGAA□ GATTCTCAAGGTGGACTACAAACTGCCGGAGCACATTT□ CCAAGGCAGCATCCCATCTCATTTCCAAGCTGCTGGTC□ CTTAAT
