# Supplementary material for: A Genome-Wide RNAi Screen to Dissect Centriole Duplication and Centrosome Maturation in Drosophila
Source: PLoS Biol. 2008 Sep 16;6(9):e224. doi: 10.1371/journal.pbio.0060224 (PMC2535660; doi:10.1371/journal.pbio.0060224)
Supplement: Supplementary file 9 [file pbio.0060224.st004.pdf]

Supplemetary Table 4

CG	FBgn	Name	cDNA/Genomic	Forward Primer NT	Reverce Primer NT	Reverce Primer CT	Sequenced
CG8233	FBgn0033897		LD22513	GGGG ACA AGT TTG TAC AAA AAA GCA GGC Ttg ATGAAGGAATCGGGAAG	GGG GAC CAC TTT GTA CAA GAA AGC TGG GTg CTACATTTTATTATG	GGG GAC CAC TTT GTA CAA GAA AGC TGG GTg CATTTTATTATGGGTTAAAG	X
CG1135	FBgn0035489		GH01794	GGGG ACA AGT TTG TAC AAA AAA GCA GGC TtgATGGAGGCATCAAGAATAAC	GGG GAC CAC TTT GTA CAA GAA AGC TGG GTgCTAGTTGAGGGGATTCGATGTCTTG	GGG GAC CAC TTT GTA CAA GAA AGC TGG GTgGTTGAGGGGATTCGATGTCTTG	
CG17295	FBgn0032034		SD16838	GGGG ACA AGT TTG TAC AAA AAA GCA GGC TtgATGCCTCACAAACGTAG	GGG GAC CAC TTT GTA CAA GAA AGC TGG GTgCTAGTTGTATGTTCTCCTATATG	GGG GAC CAC TTT GTA CAA GAA AGC TGG GTgGTTGTATGTTCTCCTATATG	
CG7186	FBgn0026371	Sak	RE70136	(Bettencourt-Dias, 2005)			
CG9045	FBgn0002914	Myb	LD22943	GGGG ACA AGT TTG TAC AAA AAA GCA GGC TtgATGGCAAGTGCGAGCACTGAAAAC	GGG GAC CAC TTT GTA CAA GAA AGC TGG GTgTTAGAGCGAACCAAAGCGGTCGAAC	GGG GAC CAC TTT GTA CAA GAA AGC TGG GTgGAGCGAACCAAAGCGGTCGAAC	
CG4786	FBgn0037012		RE54525	GGGG ACA AGT TTG TAC AAA AAA GCA GGC TtgATGGAAAAGTTAATCACAATTTG	GGG GAC CAC TTT GTA CAA GAA AGC TGG GTgTCACAACTTTTGCTCCGTCTTC	GGG GAC CAC TTT GTA CAA GAA AGC TGG GTgCAACTTTTGCTCCGTCTTC	
CG6235	FBgn0004889	Tws	-	-			
CG15524	FBgn0039731	Sas-6	-	(Peel, 2007)			
CG13162	FBgn0033718	Ana3	-	-	-	-	
CG1483	FBgn0002645	Map205	LD12965	GGGG ACA AGT TTG TAC AAA AAA GCA GGC TtgATGGAACACCACGAGGACAAC	GGG GAC CAC TTT GTA CAA GAA AGC TGG GTgTTAAAAATCCAAAAGGGGCACTTCAGCATTATG	GGG GAC CAC TTT GTA CAA GAA AGC TGG GTgAAAATCCAAAAGGGGCACTTCAGCATTATG	X
CG6631	FBgn0039206	Ana1	LD07765	GGGG ACA AGT TTG TAC AAA AAA GCA GGC TtgATGAACGATGCAATGGAAG	GGG GAC CAC TTT GTA CAA GAA AGC TGG GTgTCATTTTCGGGGCGACTTTCGATG	GGG GAC CAC TTT GTA CAA GAA AGC TGG GTgTTTTCGGGGCGACTTTCGATG	X
CG8472	FBgn0000253	Cam	LD02334	GGGG ACA AGT TTG TAC AAA AAA GCA GGC TtgATGGCCGATCAGCTGACAGAG	GGG GAC CAC TTT GTA CAA GAA AGC TGG GTgTCACTTCGATGTCATCATAGTCACGAATTC	GGG GAC CAC TTT GTA CAA GAA AGC TGG GTgCTTCGATGTCATCATAGTCACGAATTC	
CG12306	FBgn0003124	Polo	LD11851	GGGG ACA AGT TTG TAC AAA AAA GCA GGC TtgATGGCCGCGAAGCCCGAG	GGG GAC CAC TTT GTA CAA GAA AGC TGG GTgTTATGTGAACATCTTCTCCAGCATTTTC	GGG GAC CAC TTT GTA CAA GAA AGC TGG GTgTGTGAACATCTTCTCCAGCATTTTC	
CG4832	FBgn0013765	Cnn	we have	(Lucas, 2007)			
CG31389	FBgn0051389		use genomic	GGGG ACA AGT TTG TAC AAA AAA GCA GGC TtgATGGGAAAATGGGGGAAAAATG	GGG GAC CAC TTT GTA CAA GAA AGC TGG GTgTCAGCAATTCAGCCCTGCTCCCAATTG	GGG GAC CAC TTT GTA CAA GAA AGC TGG GTgGCAATTCAGCCCTGCTCCCAATTG	
CG13459	FBgn0036472	cp309/D-plp	we have	(Martinez-Campos,2004)			
CG17081	FBgn0036480		LD35990	GGGG ACA AGT TTG TAC AAA AAA GCA GGC TtgATGAATATCAACGATG	GGG GAC CAC TTT GTA CAA GAA AGC TGG GTgTTAAAGAGTCTTCGATGGCAC	GGG GAC CAC TTT GTA CAA GAA AGC TGG GTgAAGAGTCTTCGATGGCAC	X
CG13387	FBgn0020497	emb	LD45706	GGGG ACA AGT TTG TAC AAA AAA GCA GGC TtgATGGCGACAATGTTGACATC	GGG GAC CAC TTT GTA CAA GAA AGC TGG GTgCTATTCGTCCTGCATATCCTC	GGG GAC CAC TTT GTA CAA GAA AGC TGG GTgTTCGTCCTGCATATCCTC	
CG11175	FBgn0034530		LD20239	GGGG ACA AGT TTG TAC AAA AAA GCA GGC TtgATGAATTATCAGAAGGCCAGTG	GGG GAC CAC TTT GTA CAA GAA AGC TGG GTgTCAAAGAGCCACATCGCGGTACTTC	GGG GAC CAC TTT GTA CAA GAA AGC TGG GTgAAGAGCCACATCGCGGTACTTC	
CG14617	FBgn0031191		GH03511	GGGG ACA AGT TTG TAC AAA AAA GCA GGC TtgATGGATGCGACGTGGGCAATG	GGG GAC CAC TTT GTA CAA GAA AGC TGG GTgCTAGGGCTTCTTGCGATTGCCCAG	GGG GAC CAC TTT GTA CAA GAA AGC TGG GTgGGGCTTCTTGCGATTGCCCAG	X
CG17286	FBgn0027500	D-Spd2	-	(Dix, 2007)			
CG2919	FBgn0037348	Asterless	GH02902	GGGG ACA AGT TTG TAC AAA AAA GCA GGC TtgATGAACACGCCAGGTATAAG	GGG GAC CAC TTT GTA CAA GAA AGC TGG GTgGAAAGCCCAAAGGCAATGGTCACAGCTAA	GGG GAC CAC TTT GTA CAA GAA AGC TGG GTgGCTGTGACCATTGCCTTTGGGCTTTC	X
CG10061	FBgn0011020	D-Sas4 / l(3)s2214	we have	(Basto, 2006)			
CG10346	FBgn0032705	Grip71wd	RE05579	GGGG ACA AGT TTG TAC AAA AAA GCA GGC TtgATGCATGTTATTTCCACGTCTAAAAAAAC	GGG GAC CAC TTT GTA CAA GAA AGC TGG GTgTCATTTCTGCGTCAACCGGCGGGGGATTC	GGG GAC CAC TTT GTA CAA GAA AGC TGG GTgTTTCTGCGTCAACCGGCGGGGGATTC	X
CG3980	FBgn0031575		RE44293	GGGG ACA AGT TTG TAC AAA AAA GCA GGC TtgATGAGTGGTAAGAAGCCAGTTC New!!! Ordered 15.05	GGG GAC CAC TTT GTA CAA GAA AGC TGG GTgTCATGGATCTTTATCAAGATTTTC	GGG GAC CAC TTT GTA CAA GAA AGC TGG GTgTGGATCTTTATCAAGATTTTC	
CG8262	FBgn0027513	Ana2	LD22033	GGGG ACA AGT TTG TAC AAA AAA GCA GGC TtgATGTTTGTTCCCGAAAC	GGG GAC CAC TTT GTA CAA GAA AGC TGG GTgTCACAACAGCTTCGGCTGGTTC	GGG GAC CAC TTT GTA CAA GAA AGC TGG GTgCAACAGCTTCGGCTGGTTC	X
CG17291	FBgn0005776	PP2A-29B	-	-			
CG7109	FBgn0004177	Mts	-	-			
CG3157	FBgn0004176	gTub23C	LD40196	Gift Monics bettencourt-Dias			
CG3068	FBgn0000147	aur	LD39409	(Baros, 2005)			
